# Increased projected changes in quasi-resonant amplification and persistent summer weather extremes in the latest multimodel climate projections

**DOI:** 10.1038/s41598-024-72787-0

**Published:** 2024-09-23

**Authors:** Sullyandro O. Guimarães, Michael E. Mann, Stefan Rahmstorf, Stefan Petri, Byron A. Steinman, Daniel J. Brouillette, Shannon Christiansen, Xueke Li

**Affiliations:** 1 Potsdam Institute for Climate Impact Research (PIK), Member of the Leibniz Association, Potsdam, Germany; 2 University of Potsdam, Potsdam, Germany; 3 Department of Earth & Environmental Science, University of Pennsylvania, Philadelphia, PA USA; 4 Department of Earth and Environmental Sciences, University of Minnesota Duluth, Duluth, MN USA; 5 Purdue University, West Lafayette, IN USA

**Keywords:** Atmospheric dynamics, Climate-change impacts, Fluid dynamics

## Abstract

High-amplitude quasi-stationary atmospheric Rossby waves with zonal wave numbers 6–8 associated with the phenomenon of quasi-resonant amplification (QRA) have been linked to persistent summer extreme weather events in the Northern Hemisphere. QRA is not well-resolved in current generation climate models, therefore, necessitating an alternative approach to assessing their behavior. Using a previously-developed fingerprint-based semi-empirical approach, we project future occurrence of QRA events based on a QRA index derived from the zonally averaged surface temperature field, comparing results from CMIP 5 and 6 (Coupled Model Intercomparison Project). There is a general agreement among models, with most simulations projecting substantial increase in QRA index. Larger increases are found among CMIP6-SSP5-8.5 (42 models, 46 realizations), with 85% of models displaying a positive trend, as compared with 60% of CMIP5-RCP8.5 (33 models, 75 realizations), with a reduced spread among CMIP6-SSP5-8.5 models. CMIP6-SSP3-7.0 (23 models, 26 realizations) simulations display qualitatively similar behavior to CMIP6-SSP5-8.5, indicating a substantial increase in QRA events under business-as-usual emissions scenarios, and the results hold regardless of the increase in climate sensitivity in CMIP6. Projected aerosol reductions in CMIP6-SSP3-7.0-lowNTCF (5 models, 16 realizations) lead to halting effect in QRA index and Arctic Amplification during the 1st half of the twenty-first century. Our analysis suggests that anthropogenic warming will likely lead to an even more substantial increase in QRA events (and associated summer weather extremes) than indicated by past analyses.

## Introduction

The prospective increase in the frequency and/or severity of various types of extreme weather events, such as heat waves, severe storms, floods, and drought, represents one of the primary societal threats of human-caused climate change. A number of studies (e.g. ^1^ and ^2^) have suggested that human-caused warming may result in extremes that have no prior analog observed. The most recent assessment of the Intergovernmental Panel on Climate Change (IPCC)^3^ emphasizes the increased incidence of simultaneous extreme events in multiple regions and locations (“compound extreme events”), placing increased stress on civil infrastructure for disaster relief and mitigation.

A better understanding of the underlying physical mechanisms can form the basis for improved projections of extreme weather events under anthropogenic warming^4^, aiding the assessment and potential mitigation of associated extreme weather risk. Importantly, an array of extreme, persistent summer weather events in recent decades are thought to have either directly or indirectly resulted from the atmospheric phenomenon of quasi-resonant amplification (QRA) of planetary waves. These events include European heat waves in 2003, 2006, 2015 and 2018, the Russian heat wave and Pakistan flood in 2010, the Texas heat wave and drought in 2011, record Balkan floods in 2014, Alberta wildfire in 2016, and the 2021 Pacific Northwest heatwave^5–10^

Atmospheric models are valuable tools for investigating individual teleconnections and their underlying causes. Studies examining the change in frequency of certain high-impact circulation patterns, such as amplified and persistent quasi-stationary waves in the boreal summer jet stream, have been conducted using reanalysis data and climate models, indicating the influence of resonant waves on heat and other extremes^11–18^.

A theoretical framework for diagnosing the impact of climate change on QRA was provided by Petoukhov *et al.* ^19^ and further developed by Kornhuber *et al.* ^20,21^. The basic concept is that planetary-scale waves with zonal wave numbers 6 or higher can exhibit unusually high-amplitude when quasi-stationary free waves with the same (or close to the same) wave number are trapped within midlatitude waveguides. These waves are amplified by a quasi-resonance mechanism. The occurrence of QRA is closely associated with a prominent double-jet configuration, enhanced atmospheric blocking, and weakened upward wave propagation^12^. Favorable conditions for occurrence of QRA is expected to increase, likely linked to Arctic amplification^22^. This is due various processes, including snow- and ice-albedo feedbacks associated with anthropogenic greenhouse warming.

However, it is still challenging for current climate models to accurately capture high wavenumber planetary wave dynamics, which are crucial for detecting QRA-favorable conditions. The uncertainty in the atmospheric midlatitude circulation simulated by CMIP5 and CMIP6 models related to jet stream, Arctic amplification, and associated impacts on extremes was noted also in other studies^23–28^, that directly link to the QRA framework diagnoses based on daily variations of temperature and wind fields^19–21^. Despite CMIP6 models displaying some reductions in bias compared to earlier (CMIP3 and CMIP5) model intercomparisons, mostly due to increased model resolution^29,30^, the biases relevant to planetary wave dynamics remain substantial, including e.g. blocking frequencies in regions of the Northern Hemisphere^18,31^.

In contrast, the models reasonably simulate changes in global surface temperature patterns. For these reasons, Mann *et al.* ^22,^^32^ proposed a semi-empirical approach, namely using a temperature-based fingerprint, to assess possible QRA changes with guidance from climate models. This approach leverages the robust relationship between the meridional variation in surface temperature and upper-level zonal winds within regions where QRA phenomena have been identified^19^.

From the linearized quasigeostrophic barotropic potential vorticity equation (see Eq. 1 in ^32^), we see that wave dynamics underlying QRA are dependent on the second derivative of the zonal mean zonal wind (*ū*) with latitude (*d*^*2*^*ū/dφ*^*2*^). Moderate model biases in *ū(φ)* propagate to substantial (i.e. several hundred percent) biases^22^ after two subsequent numerical differentiations, making it difficult for even state-of-the-art climate models to accurately resolve this term.

The current models are thus unable to accurately resolve the real-world wave dynamics underlying QRA. Mann *et al.* ^32^ make use of the fact that *ū(φ)*, through the thermal wind relationship, is closely related to the meridional temperature profile *T(φ),* developing a temperature-based fingerprint for QRA, based on the anomalous temperature profile *T’(φ)* associated with QRA, that can be obtained from modern reanalysis data.

We first analyze the CMIP5 and CMIP6 multimodel historical ensembles (see Materials and Methods) to assess the relative faithfulness with which the two relevant quantities, zonal mean *T(φ)* and zonal mean *d*^*2*^*ū(φ)/dφ*^*2*^, are reproduced in the climate models relative to ERA5 reanalysis, testing whether this indirect approach to assessing QRA likely to be more reliable than a direct analysis of QRA events in the model simulations (Table 1). We observe a small ensemble mean error of roughly 0.2% for *T(φ)* for both CMIP phases, or, alternatively, a still modest roughly 21% (23%) ensemble mean error for d*T(φ)*/d*φ* in CMIP6 (CMIP5), respectively. For comparison, we obtain an extremely large mean error for ensemble members in *d*^*2*^*ū(φ)/dφ*^*2*^, 341% in CMIP5, and 285% in CMIP6. The models’ ensemble spread for *T(φ),* d*T(φ)*/d*φ, and d*^*2*^*ū(φ)/dφ*^*2*^ is shown in Supplementary Figures S31-S40.

**Table 1:** Temperature *T(φ)* and zonal wind *ū(φ)* RMSE for CMIP5 (1979–2005) and CMIP6 (1979–2014) Historical multimodel ensemble over 25N-75N (2.5°) JJA seasonal means compared to ERA5 (1979–2014).

RMSE related to ERA5	Mean error for ensemble members		Error for ensemble mean	
	CMIP5	CMIP6	CMIP5	CMIP6
*T(φ)*	0.4% (150)	0.5% (66)	0.2% (150)	0.2% (66)
*dT(φ)/dφ*	− 33.8% (150)	− 30.6% (66)	− 23.3% (150)	− 20.6% (66)
*ū(φ)*	28.0% (74)	17.6% (66)	19.0% (74)	10.7% (66)
*dū(φ)/dφ*	192.4% (74)	172.8% (66)	93.4% (74)	120.8% (66)
*d*^*2*^*ū(φ)/dφ*^*2*^	− 341.3% (74)	− 285.3% (66)	− 160.0% (74)	− 205.2% (66)

In brackets is the number of models.The RMSE presented is the RMSE divided by the respective reference variable, and multiplied by 100 to obtain the percentage RMSE.

We conclude that while direct evaluation of QRA behavior is not achievable using the currently available multimodel CMIP ensembles, our indirect method, which uses the anomalous latitudinal temperature profile *T’(φ)* as a proxy for QRA behavior in the models, produces relevant findings. Naturally, one presumption behind our methodology is that the fingerprint generated by previous observations will continue to hold true in the future, connected to the assumption that planetary waves stimulated by orographic or thermal forces will maintain a similar structure. For the analysis, two distinct time intervals were chosen, the first and second half of the twenty-first century, under the experiments CMIP6-SSP5-8.5, CMIP6-SSP3-7.0, CMIP6-SSP3-7.0-lowNTCF, and CMIP5-RCP8.5.

## Results

**Fig. 1:**
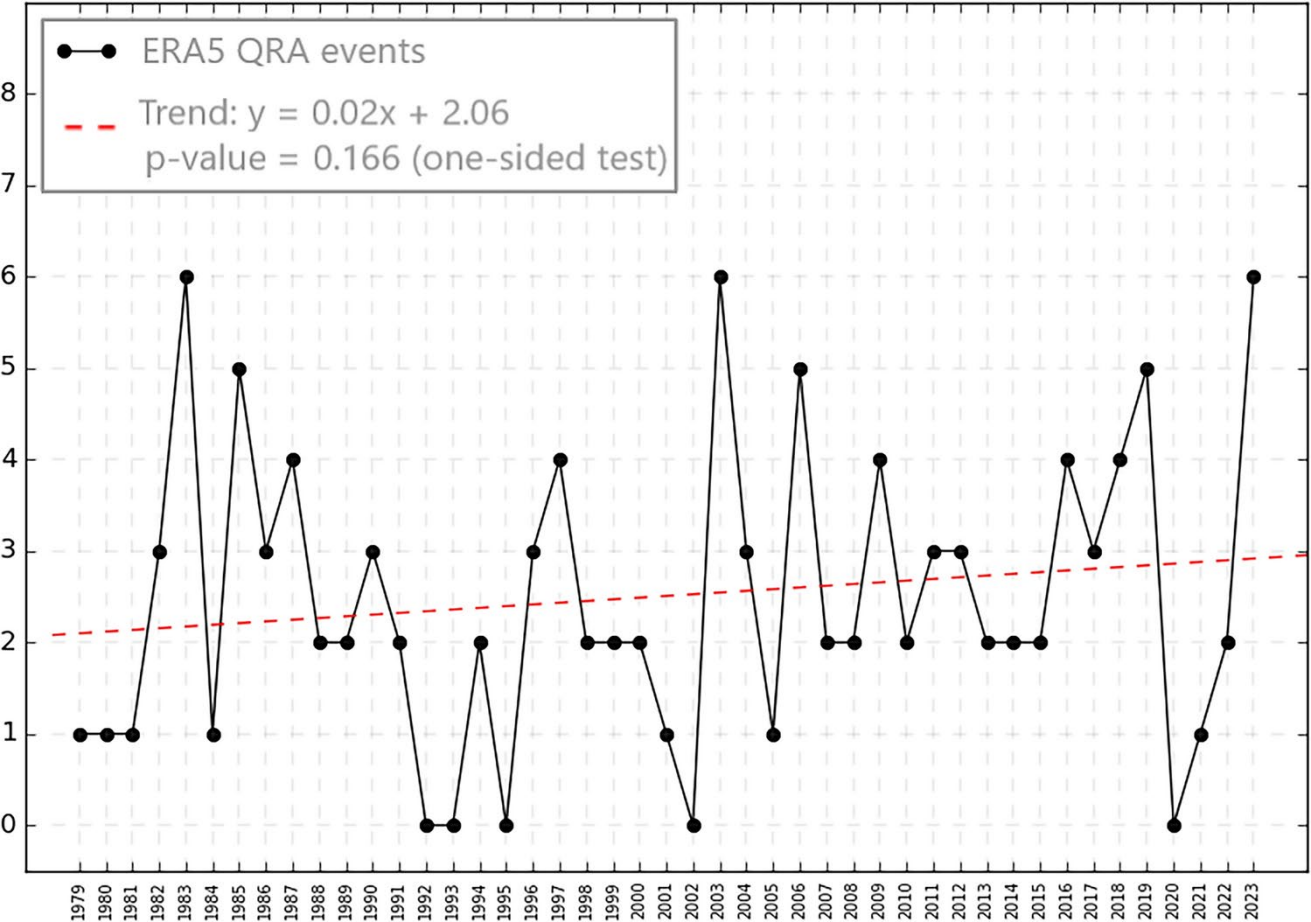
Number of QRA events for JJA wave numbers from 6 to 8 for the ERA5 reanalysis. The linear trend over the period 1979–2023 is shown (the indicated *p* value is based a one-sided test given the hypothesis that greenhouse warming increases QRA occurrence).

Real-world QRA events as far back as the late 1940s can be diagnosed from reanalysis data^5^^,^^19^^,^^20^^,^^22^. QRA-favorable conditions adopted here consist of cases in which planetary waves with numbers 6–8 stay stationary for a long duration (10 ≤ duration ≤ 15 days), identified in confined zonal mean profile to the latitude range (25°N to 75°N). The annual average number of events has increased (Fig. 1) from an average of roughly 2 events per year in the late 1970s to roughly 3 per year recently, constituting an increase by roughly 50% over the past four decades. The trend is not statistically significant (*p* = 0.166 for a one-sided test) owing to the substantial year-to-year variability and short nature of the dataset. However, a recent extension of the analysis using ERA5 data back to 1950^33^ provides evidence for a statistically-significant (*p* < 0.01) trend over the past half century. Mann *etal.*^22^ find that the signal of human-caused climate change in the QRA fingerprint has only recently emerged from the noise of natural variability.

**Fig. 2:**
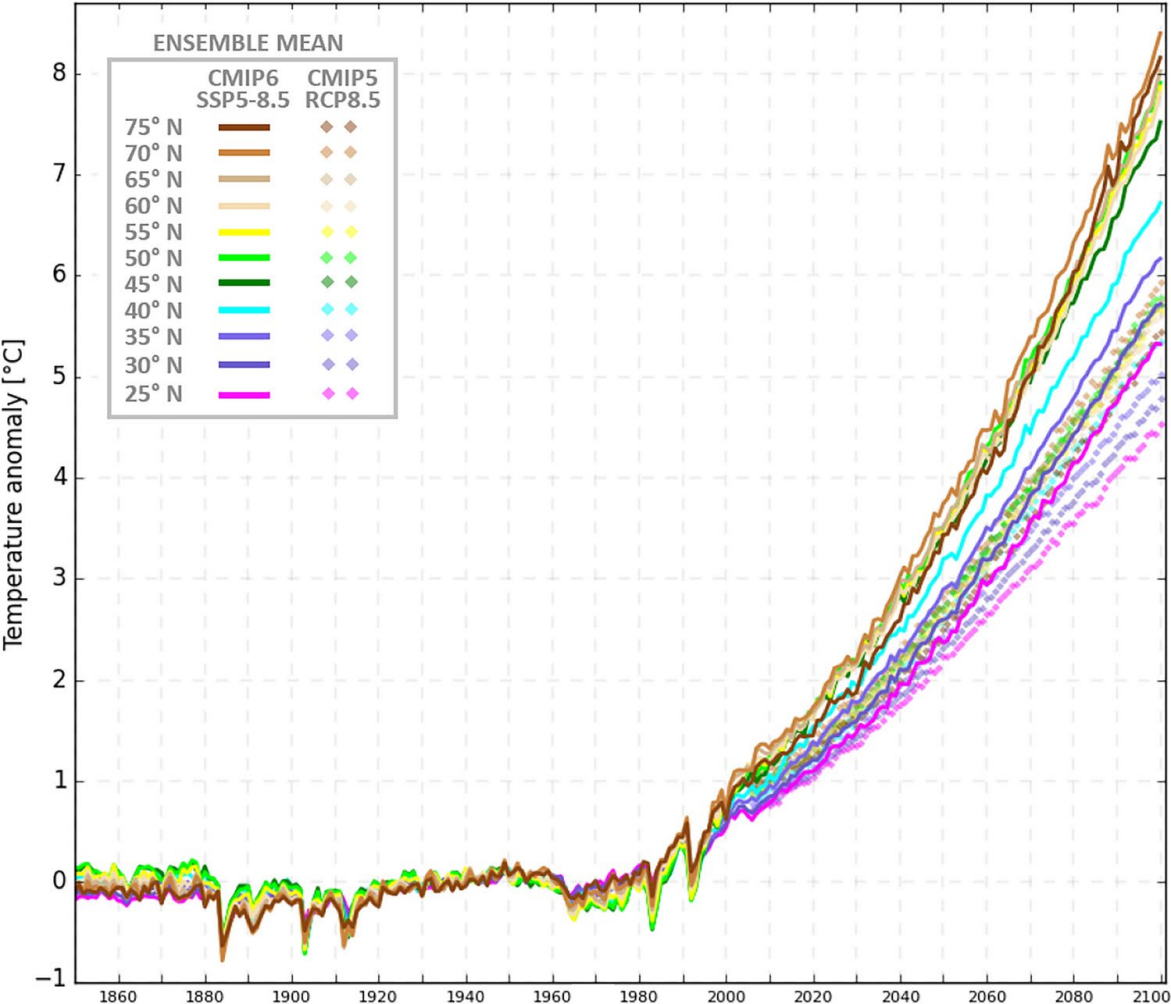
Temperature anomaly JJA seasonal means for CMIP5-RCP8.5 and CMIP6 Historical and SSP5-8.5. This anomaly follows ^22^.

**Fig. 3:**
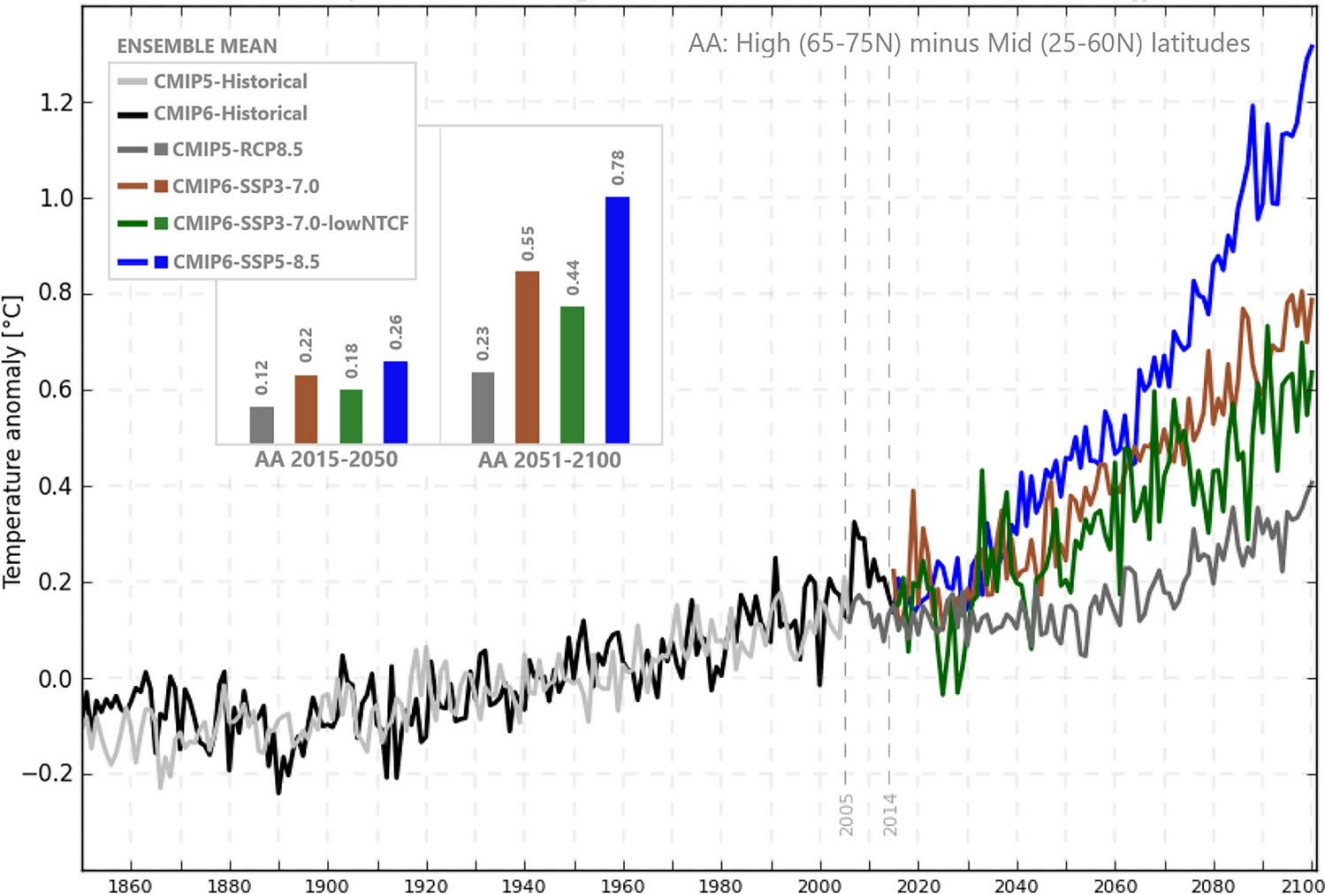
Arctic amplification (AA) from Temperature anomaly JJA seasonal means for CMIP5 and CMIP6. This anomaly follows ^22^. The values represented as bars were calculated by the mean of the AA over the periods 2015–2050 and 2051–2100.

**Fig. 4:**
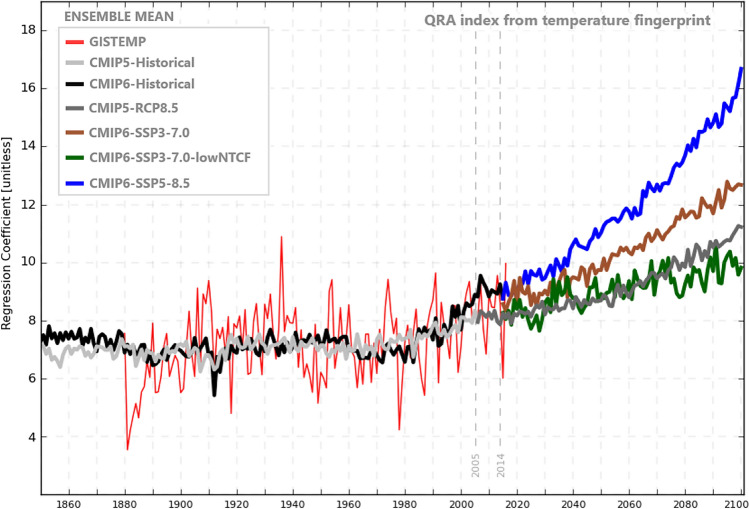
QRA index from Temperature JJA seasonal means for CMIP5 and CMIP6.

**Fig. 5:**
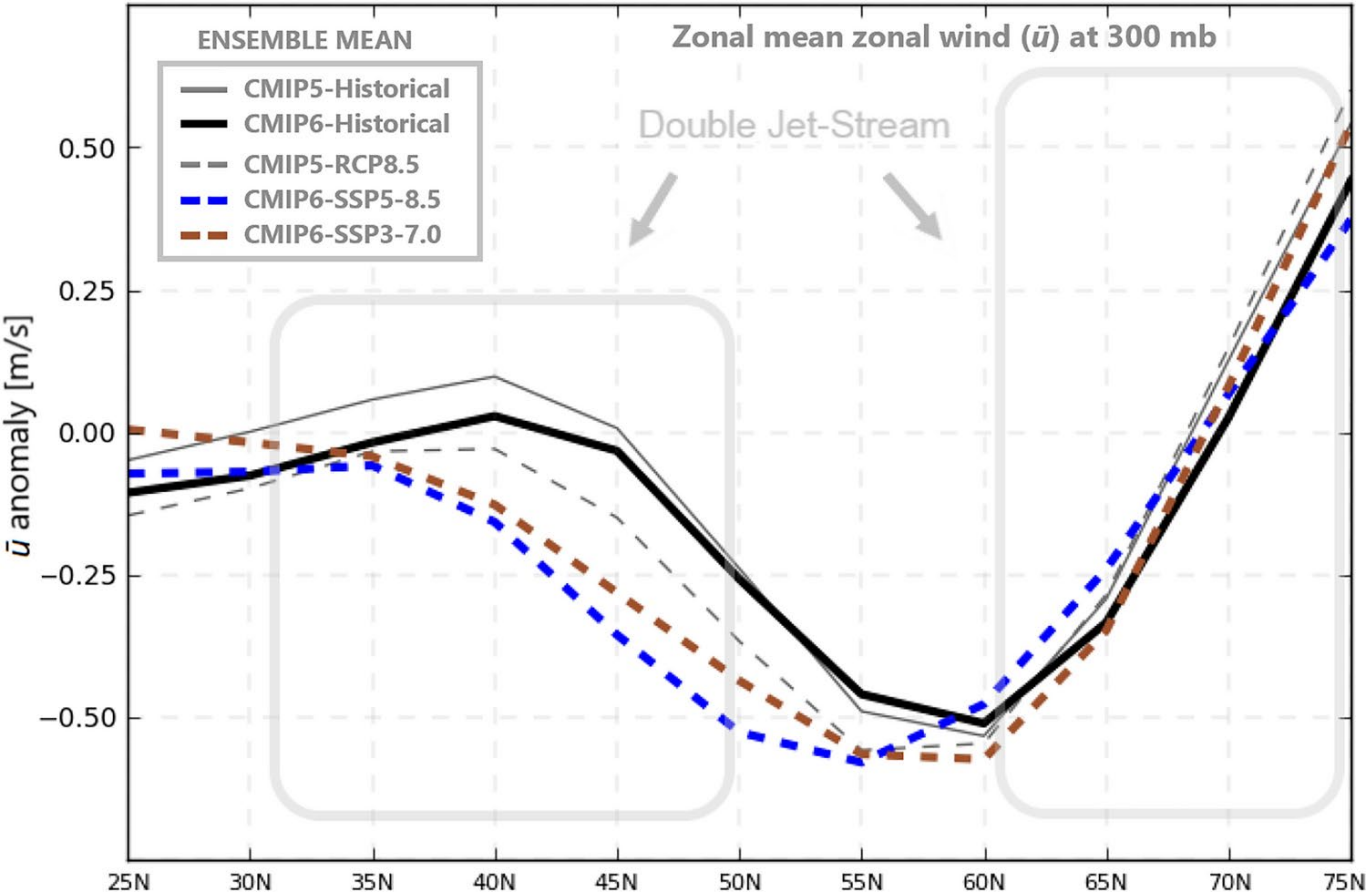
Multimodel projection of QRA index from Temperature JJA seasonal means onto zonal mean zonal wind (*ū*) anomalies for CMIP5 and CMIP6.

Figure 2 compares the multimodel mean latitudinal variation for JJA seasons in warming amplitude between CMIP5 and CMIP6 for the high-end emissions scenario (RCP8.5/SSP5-8.5 respectively). We see that there is overall greater warming predicted by CMIP6 models (which has been widely noted - ^34)^ but, importantly, greater latitudinal spread of warming, with high latitudes warming by over 2 ℃ more in CMIP6 than in CMIP5. This is also evident in Fig. 3, which includes results from a mid-range (SSP3-7.0) emissions scenario from CMIP6. Even that lower scenario shows greater warming and latitudinal spread in CMIP6 than in the CMIP5 high-range scenario. Mann *et al*.^22,32^ showed that, while the latitudinal temperature fingerprint associated with QRA for JJA seasons has more structure than a simple Arctic amplification index, it does project substantially onto such an index. Accordingly, we find considerably greater increases in the multimodel mean QRA index in the CMIP6 high-end scenario than in the CMIP5 high-end scenario (Fig. 4). Over the twenty-first century we see a roughly 40% increase in CMIP5 but more than doubling (109%) in CMIP6. Consistent with past work, the QRA index projects onto a “double jet” pattern, where upper-level westerly flow is greatly reduced in the mid-latitudes but not subtropical or sub-polar latitudes (Fig. 5).

It is important to note that the enhanced warming in CMIP6 projections is largely the result of a relatively small number of outlier models with very high climate sensitivity^34^^,^^35^. It is therefore instructive to examine the variation of results across the ensemble (Supplementary Figures S6-S9 – Temperature anomaly for JJA seasonal means for historical and future simulations).

**Fig. 6:**
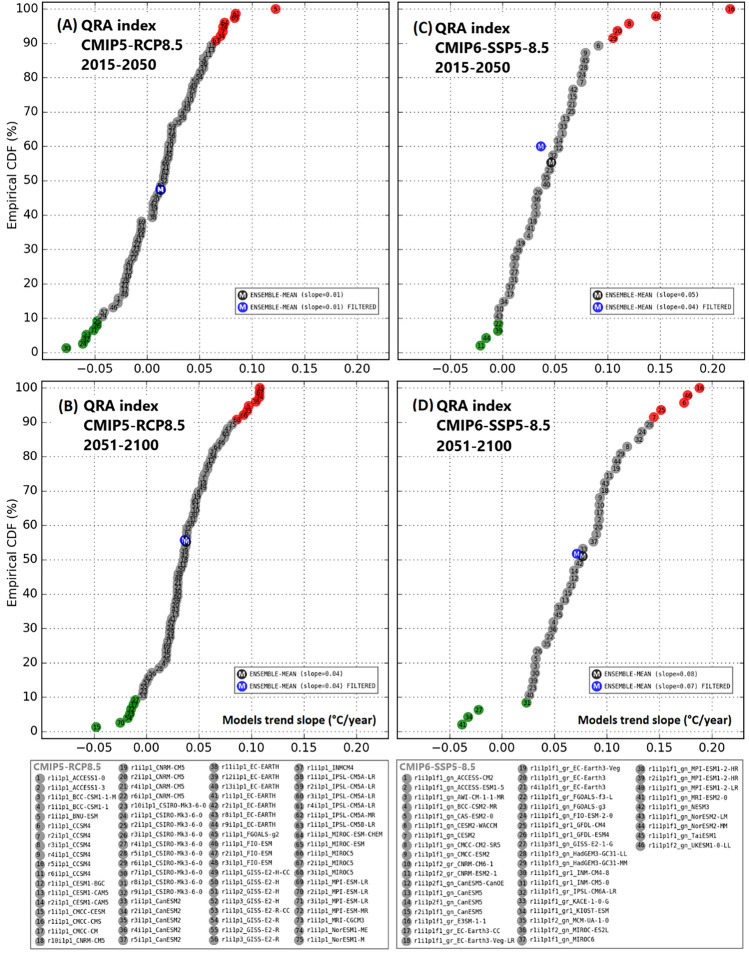
Average QRA index from Temperature JJA seasonal means for CMIP5-RCP8.5 (**A**, **B**) and CMIP6-SSP5-8.5 (**C**, **D**). The list of CMIP5 models is shown below frame (**B**), respectively CMIP6 models below frame (**D**). Ensemble-Mean Filtered (blue marker) refers to the mean over all models excluding models in ECS > 4.7 ℃, upper 10th percentile (red markers), and lower 10th percentile (green markers).

In Fig. 6, we examine the distribution of trends in both CMIP5 and CMIP6, focusing on two distinct time intervals corresponding to the QRA index for JJA seasonal mean on the first (2015–2050) and 2nd (2051–2100) half of the twenty-first century during which aerosol reductions play a substantial and modest role, respectively. As noted by^32^, substantial projected anthropogenic aerosol reductions in summer lead to near zero or even negative QRA trend during the 1st half of the twenty-first century in the CMIP5 projections, as well enhanced mid-latitude warming and reduced polar amplification, mitigating increases in QRA activity. Very few models in CMIP6 however are seen to show such behavior. In CMIP5, only one realization among the entire ensemble exhibited a trend exceeding 0.1 events/year. For CMIP6, 5 realizations show such trends, and the largest trend among all realizations (~ 0.2 events/year) is roughly twice as large. For the latter half of the twenty-first century, where greenhouse warming dominates, CMIP6 once again shows far greater trends in QRA index among most models, with a small number of realizations in particular (5) showing considerably greater increase in QRA index than was observed in any of the CMIP5 realizations.

To verify the robustness of the QRA projections, given the greater climate sensitivity of some CMIP6 models, we performed a filtering case where we excluded from the multimodel mean all models with Effective Climate Sensitivity (ECS) above 4.7 ℃ and QRA trends within the upper and lower 10th percentile of results (represented by a blue marker in Fig. 6). The QRA trend after that filtering resulted in a difference (in relation to the case without filter) of 24.4% in CMIP6-SSP5-8.5 (seven models with ECS > 4.7 ℃ removed) for the first time-slice analyzed (2015–2050, Fig. 6C), and for the second (2051–2100, Fig. 6D) the difference was 8.1%, confirming the consistency of the multimodel mean against outliers for the higher temperature change period. The other scenarios vary from 8% (CMIP5-RCP8.5 2015-2050, Fig. 6A) difference in QRA trend, to 5.4% (CMIP5-RCP8.5 2051-2100, Fig. 6B).

Additionally, treating the results in two different cases of filtering for the multimodel mean, first case excluding only models with ECS > 4.7 ℃, and the second case only the models with QRA trend (Fig. 6) upper 10th percentile, and lower 10th percentile, we found that for the first case CMIP6-SSP5-8.5 has the differences of 24.4% (2015–2050, Fig. 6-C) and 15.4% (2051–2100, Fig. 6D), and in the second case 14% (Fig. 6C) and 4% (Fig. 6D), respectively. For CMIP5-RCP8.5 multimodel mean, no differences were found for the first case in relation to non-filtered multimodel mean, and 8% (2015–2050, Fig. 6A) and 5.4% (2051–2100, Fig. 6B) for the second case. CMIP6-SSP3-7.0 has fewer models compared to the other scenarios, for the time-slice 2015–2050 the differences were 5.1% for both cases, and 14.6% and 7% in 2051–2100, for the first and second cases. This indicates that independent of the few models with high ECS, the multimodel mean is still robust.

**Fig. 7:**
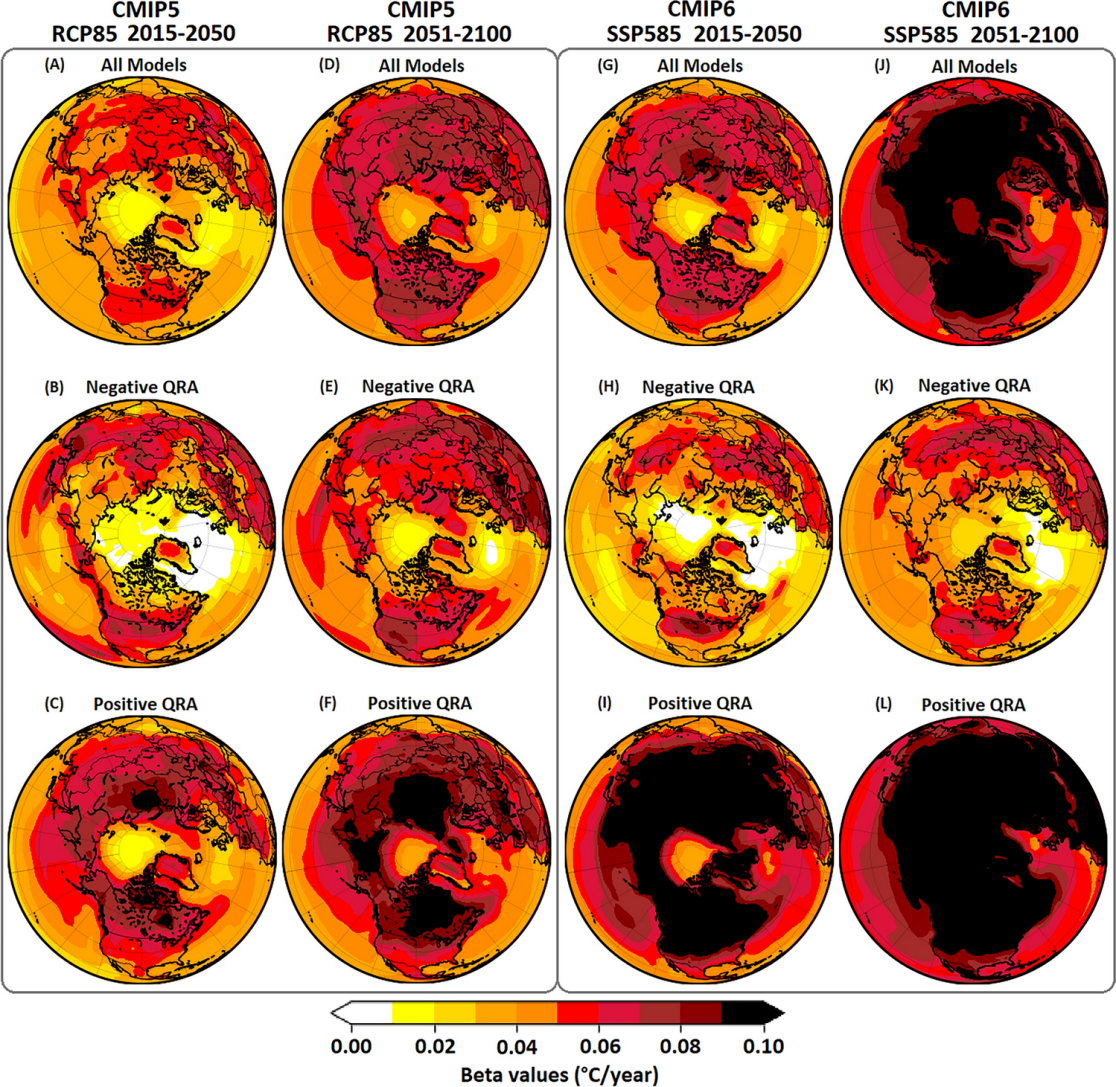
Mean surface temperature trend patterns (JJA seasonal means) for CMIP5-RCP8.5 and CMIP6-SSP5-8.5. (**A**, **D**, **G**, **J**) multimodel ensemble, (**B**, **E**, **H**, **K**) most negative QRA-trending ensemble members, and (**C**, **F**, **I**, **L**) most positive QRA-trending ensemble members (“most” is defined as upper 10th percentile of multimodel ensemble).

These filtered results are consistent with the spatial patterns of warming which show considerably greater polar amplification of warming in CMIP6 during both sub-intervals of the twenty-first century, with the largest QRA-trending models showing the greatest polar amplification (Fig. 7).

## Discussion

We compare results of high emission scenarios from CMIP5 and CMIP6 climate projections, using a previously developed fingerprint-based semi-empirical approach to project future occurrence of QRA events based on a QRA index derived from the zonally averaged surface temperature field. Models are in general agreement, with most simulations predicting a significant increase in QRA index, in accordance with past work demonstrating increased QRA occurrence with greenhouse warming^13^^,^^14^^,^^18^^,^^31^. The new generation of models introduces a greater spread in the results; however removal of outliers (models with high sensitivity to forcing) reveals the multimodel mean behavior to be robust.

The new CMIP6-SSP5-8.5 ensemble shows considerably greater projected Arctic amplification in the future than its RCP8.5 counterpart. Amplification is seen even in the first half of the 21st Century where the RCP8.5 projections show no clear pattern of polar amplification of summer warming. Similar behavior appears also in CMIP6-SSP3-7.0 and CMIP6-SSP3-7.0-lowNTCF (Fig. 3). Mann *et al*.^32^ found a similar pattern when comparing RCP8.5 to 1PCTCO2 simulations, which they attributed to the aerosol decline in RCP8.5 in the early twenty-first century as discussed above.

It is useful to compare the CMIP6-SSP3-7.0-lowNTCF scenario with low aerosol forcing (though consisting of a modest number of realizations: five models, 16 realizations) with the otherwise similar radiative forcing scenario CMIP6-SSP3-7.0. The high aerosol simulations driven by CMIP6-SSP3-7.0 show a increase in both Arctic Amplification and QRA index during the twenty-first century (Figs. 3,4). In contrast, when aerosols are rapidly reduced in the first half of twenty-first century as in the CMIP6-SSP3-7.0-lowNTCF scenario, the model responses highlight the role played by aerosol loading reduction, as noted previously^32^^,^^36^ which competes with the effects of greenhouse warming, reducing Arctic amplification, and mitigating potential increases in QRA-related persistent extreme weather events.

In both aerosol scenarios, the second half of the twenty-first century has the remarkable dominance role of the greenhouse’s radiative forcing, reaching 7.0 W/m^2^, while estimative of total aerosol forcing contribution ranges from −2.0 to −0.4 W/m^2 ^^37^^,^^38^^,^^39^^,^^40^.

The improvement in resolution and components in CMIP6 aiming to represent all relevant physical processes response to CO_2_, leads on the other hand to increased ECS. CMIP5 ECS values diagnosed from CO_2_ quadrupling experiments ranged from 2.1 to 4.7 ℃^41^^,^^42^, and studies including CMIP6^43^ noticed values of ECS exceeding 4.7 ℃, as registered for CanESM5.0.3 (^44^, ECS = 5.64 °C), CESM2 (^45^, ECS = 5.15 ℃), CNRM-CM6-1 (^46^, ECS = 4.90 ℃), E3SMv1 (^47^, ECS = 5.31 ℃), HadGEM3-GC3.1 (ECS = 5.55 ℃) and UKESM1 (^48^, ECS = 5.36 ℃). Additionally, Zhao^49^ classified GFDL-CM4 (ECS = 3.89 ℃) as having a relatively high ECS model. High ECS values in these models mentioned (also included in our analysis) are at least partly attributed to larger cloud feedbacks than their predecessors (^50^ – reference for the ECS values cited above).

The spread of models within the ensemble among our results in CMIP6 warming projections (Supplementary Figures S6-S9) is largely due to a relatively small number of outlier models with very high climate sensitivity.

We removed from the multimodel mean all models with high ECS (above 4.7 ℃), to confirm that the QRA projections are not strongly influenced by model sensitivity. The results from that show a 24.4% maximum influence of the remarkable models in the ensemble QRA trend for the first half of the century (Fig. 6), while the differences for the high emissions simulations (15.4% for 2051–2100) reinforce that the conclusions regarding QRA index are not dependent upon a small number of models with very high climate sensitivity.

Therefore, there is agreement among models under the considered scenarios, and the majority of simulations project a significant rise in the QRA index. In comparison to CMIP5 RCP8.5 (33 models, 75 realizations) that displays increases in 60% of the simulations in the period of 2015–2050 (85% in 2051–2100), 85% of CMIP6 SSP5-8.5 (42 models, 46 realizations) models show a positive trend already in the first half of the projections (91% in the second half; Fig. 6). There is also a smaller dispersion across SSP5-8.5 results. Under business-as-usual emissions scenarios, the CMIP6 SSP3-7.0 simulations (23 models, 26 realizations) show behavior that is qualitatively comparable to SSP5-8.5, which indicates a significant rise in QRA events.

## Conclusions

The real-world QRA-favorable events diagnosed from reanalysis data indicate a trend from an average of 7 events/year in the late 1970s to around 9 per year in recent years, amounting to an increase by roughly 30% over the past 4 decades. Several studies have identified a number of persistent, high-impact extreme events in recent decades that are associated with QRA, reinforcing the proposition advanced by Mann *et al*.^22^ that the signal of an increase in QRA-induced persistent summer weather extremes due to human-caused climate change has recently emerged from the noise of natural variability.

According to our analysis, mid-to-high future emission scenarios indicate a future increase in QRA-driven extreme weather events, assuming that the historically defined fingerprint remains valid in future decades. There remain large uncertainties, as indicated by the large intermodel spread in QRA index. However, the qualitative agreement is larger in the newer CMIP6 model generation; except for a few outliers, there is common agreement that QRA events will increase, both in the first and also second half of the 21st Century. Excluding models with high Effective Climate Sensitivity (above 4.7 ℃) produced similar results, with small differences in the multimodel mean QRA trend.

An important source of uncertainty is the role of aerosol forcing, with the reduction of aerosol loads acting to reduce the number of QRA events. We therefore analyzed two distinct time intervals, namely the first and second half of the twenty-first century. We find that aerosol reductions play a substantial role in CMIP5 and CMIP6 models. Substantial projected anthropogenic aerosol reductions lead to a near zero or even negative QRA trend during the 1st half of the century in the CMIP 5 and 6 projections. For the latter half of the twenty-first century, where greenhouse warming dominates, CMIP6 shows far greater trends in the QRA index among most models than CMIP5 projections. These findings align with warming patterns indicating greater polar amplification during both 21st-century sub-intervals, with the largest QRA-trending models showing the most significant polar amplification.

Our findings suggest that, compared to an earlier analysis based on CMIP5-generation climate model simulations, human-caused greenhouse warming will likely result in an even more substantial rise in QRA events and associated persistent summer weather extremes.

## Materials and methods

### QRA fingerprint

We used the QRA detection scheme developed by Kornhuber *et al*.^20^ and applied it to temperature profiles as in Mann *et al*.^22,^^32^ using the JJA 1979–2015 ERA-Interim reanalysis (atmosphere horizontal resolution of 2.5° × 2.5°, ^51^), including in the present study the ERA5 reanalysis (atmosphere horizontal resolution of 0.25° × 0.25°, ^52^) from 1979–2023.

For the detection scheme, the near-surface atmospheric temperature profiles were calculated (1000 hPa pressure level) over the North Hemisphere area (0°N to 90°N), with ERA-Interim and ERA5 in 2.5° spacing (37 steps). The events treated here were obtained under the condition of waves with number 6–8, focused on long duration cases (10 ≤ duration ≤ 15 days) as the QRA-favorable time intervals and confined zonal mean profiles to the latitude range (25°N to 75°N) of interest. To match the QRA fingerprint obtained from the reanalysis with the grid of the climate models, all the zonal mean profiles were interpolated to a 5° latitude and centered on zero. This yields an 11-element zero-centered row vector that defines the QRA fingerprint, that represents the main aspects of the midlatitude temperatures, including the Arctic amplification given in high latitudes (65°N-75°N).

The use of 2.5° for the reanalysis and models was made upon the need to compute the first and second derivatives involved in our QRA investigation. Finer resolution produced unnecessary noise in out derivatives, and later on the RMSE analysis.

Mann *et al*.^32^ performed a comparison of two independent periods for the QRA fingerprint, first half (1979–1997) and second half (1998–2015), with the full ERA-Interim analyzed (1979–2015), that yields profiles very similar. Under this consideration, we kept the same QRA fingerprint from ERA-Interim (1979–2015) while calculating the model-based QRA fingerprint for the present analysis.

In CMIP6, CMIP5, and observational datasets, the projection of other fields onto the QRA fingerprint was estimated by computing the linear inner product of those fields and the QRA fingerprint mentioned above.

ERA5 was used to identify recent QRA events and to rescale models’ QRA fingerprints. We constructed a time series of observed annual QRA events using the collection of all QRA events from wavenumbers 6 to 8 in the reanalysis dataset from 1979 to 2023 (Fig. 1), once ERA-Interim (1979–2015) was discontinued (Supplementary Figure S1). Therefore, the approach of the mean and variance matching with this series to scale the observational and model-based QRA fingerprint series in dimensions of the equivalent annual number of QRA events was applied.

### CMIP5 and CMIP6 simulations

#### Historical simulations

CMIP5 and CMIP6 Historical scenario experiment designed for the recent past (1850 to 2005/CMIP5 and 2014/CMIP6), with imposed changing conditions (consistent with observations), including both anthropogenic and natural forcing. We used the CMIP5 multimodel ensemble simulations of 38 different models (N = 150 realizations). And for CMIP6, 60 different models (N = 66 realizations). Each model’s characteristics are detailed in Supplementary Tables S1-S4. We used a simple area-weighted average to create zonal means at a 5° interval^22^.

#### Future projections (RCP8.5, SSP5-8.5, and SSP3-7.0)

The impact scenarios studied consist of: the most heavy emission pathway scenarios RCP8.5 from CMIP5; the CMIP6 equivalent heavy emission scenario SSP5-8.5; the SSP3-7.0 designed for aerosol impact studies, a medium–high forcing scenario with high emissions of near-term climate forcers (NTCF) such as methane and aerosol; and the SSP3-7.0-lowNTCF, following a low NFCT pathway of SSP3-7.0 design.

From CMIP5 RCP8.5 (2006–2100) we used 33 models (N = 75 realizations) for the analyses in the main article. For CMIP6 SSP5-8.5 the number of models is 42 (N = 46 realizations); for SSP3-7.0 23 models (N = 26 realizations), and for SSP3-7.0-lowNTCF 5 models (N = 16 realizations); limited to the common time period of overlap for all models and realizations (2015–2100).

We used a simple area weighted average to create zonal means at a 5° interval, as performed for the Historical experiment.

Information of models characteristics cited above for CMIP5 and CMIP6 (Supplementary Tables S1-S4) were obtained from the models’ data file header, and complemented with information from IPCC (^53^ – Table AII.5, and ^42^ – Table 9.A.1).

### Observational data

Using each of the three alternative datasets GISTEMP (1880–2015; ^54^), HadCRUT4 (1894–2015; ^55^), and Cowtan and Way (1894–2015; ^56^), we examined zonal mean boreal summer (JJA) average temperatures (surface air temperature over land and sea surface temperature over oceans). Due to the large spatial gaps in the Arctic region, which is crucial to this study, as well as the amplified high latitude warming in the Northern Hemisphere in recent decades, these three different datasets make alternative assumptions about how to account for historical data gaps. Continuous data availability for each latitude band limited the analysis’s time frame. Examples include the HadCRUT4 and Cowtan and Way datasets, which from 1894 to the present day contain at least one grid cell temperature value in each latitude band across the relevant range (25°N to 75°N). As a result, the grid cell coverage is relatively low in the early record segments and rises as we get closer to the present. We created zonal means at a 5° interval using a simple area-weighted average, as with the models’ output^22^^,^^32^.

## Supplementary Information

Supplementary Information.

## Data Availability

The data and programming codes required to reproduce this analysis are publicly available at: 10.5281/zenodo.10364064.
