# Supplementary material for: Increased projected changes in quasi-resonant amplification and persistent summer weather extremes in the latest multimodel climate projections
Source: Sci Rep. 2024 Sep 23;14:21991. doi: 10.1038/s41598-024-72787-0 (PMC11420229; doi:10.1038/s41598-024-72787-0)
Supplement: Supplementary file 1 — Supplementary Information. [file 41598_2024_72787_MOESM1_ESM.docx]

Supplementary Material for the article

**Increased projected changes in quasi-resonant amplification and persistent summer weather extremes in the latest multimodel climate projections**

**Sullyandro O. Guimarães^1,2*^,** **Michael E. Mann^3^, Stefan Rahmstorf^1,2^, Stefan Petri^1^, Byron A. Steinman^4^, Daniel J. Brouillette^5^, Shannon Christiansen^3^, Xueke Li^3^**

*^1^Potsdam Institute for Climate Impact Research (PIK), Member of the Leibniz Association, Potsdam, Germany*

*^2^University of Potsdam, Potsdam, Germany*

*^3^Department of Earth & Environmental Science, University of Pennsylvania, Philadelphia PA, USA*

*^4^University of Minnesota Duluth, Duluth, MN, USA*

*^5^Purdue University, West Lafayette, Indiana, USA*

*^*^Corresponding author, sullyandro@pik-potsdam.de*

**This document includes supplementary figures, tables, and information**

December 10, 2023

**Summary of Tables**

**Table S1**: CMIP6 multimodel ensemble. Additional information was obtained from [2] and [3].

**Table S2**: CMIP5 multimodel ensemble. Additional information was obtained from [2] and [3].

**Table S3**: CMIP5 multimodel ensemble (continuation). Additional information was obtained from [2] and [3].

**Table S4**: CMIP5 multimodel ensemble (continuation). Additional information was obtained from [2] and [3].

**Table S5**: Temperature *T(φ)* and zonal wind *ū(φ)* RMSE for CMIP5 (1979-2005) and CMIP6 (1979-2014) Historical multimodel ensemble over 25N-75N (2.5 degrees) JJA seasonal means compared to ERA-Interim (1979-2014).

**Table S6**: Temperature *T(φ)* and zonal wind *ū(φ)* RMSE for CMIP5 (1979-2005) and CMIP6 (1979-2014) Historical multimodel ensemble over 25N-75N (2.5 degrees) JJA seasonal means compared to ERA5 (1979-2014).

**Summary of Figures**

**Figure S1**: Number of QRA events for JJA wave numbers from 6 to 8 for the ERA5 and ERA-Interim reanalysis.

**Figure S2**: Temperature JJA seasonal means for CMIP5 Historical and RCP85 simulations.

**Figure S3**: Temperature JJA seasonal means for CMIP6 Historical and SSP585 simulations.

**Figure S4**: Temperature JJA seasonal means for CMIP6 Historical and SSP370 simulations.

**Figure S5**: Temperature JJA seasonal means for CMIP6 Historical and SSP370-lowNTCF simulations.

**Figure S6**: Temperature anomaly JJA seasonal means for CMIP5 Historical and RCP85 simulations. This anomaly follows [4].

**Figure S7**: Temperature anomaly JJA seasonal means for CMIP6 Historical and SSP585 simulations. This anomaly follows [4].

**Figure S8**: Temperature anomaly JJA seasonal means for CMIP6 Historical and SSP370 simulations. This anomaly follows [4].

**Figure S9**: Temperature anomaly JJA seasonal means for CMIP6 Historical and SSP370-lowNTCF simulations. This anomaly follows [4].

**Figure S10**: Temperature anomaly JJA seasonal means for CMIP5 Historical and RCP85. This anomaly follows [4].

**Figure S11**: Temperature anomaly JJA seasonal means for CMIP6 Historical and SSP370. This anomaly follows [4].

**Figure S12**: Temperature anomaly JJA seasonal means for CMIP6 Historical and SSP585. This anomaly follows [4].

**Figure S13**: Temperature anomaly JJA seasonal means for CMIP6 Historical and SSP370-lowNTCF. This anomaly follows [4].

**Figure S14**: Temperature anomaly JJA seasonal means for CMIP6 Historical, SSP370, and SSP370-lowNTCF. This anomaly follows [4].

**Figure S15**: Arctic amplification from Temperature anomaly JJA seasonal means for CMIP5 Historical and RCP85. This anomaly follows [4].

**Figure S16**: Arctic amplification from Temperature anomaly JJA seasonal means for CMIP6 Historical and SSP585. This anomaly follows [4].

**Figure S17**: Arctic amplification from Temperature anomaly JJA seasonal means for CMIP6 Historical and SSP370. This anomaly follows [4].

**Figure S18**: Arctic amplification from Temperature anomaly JJA seasonal means for CMIP6 Historical and SSP370-lowNTCF. This anomaly follows [4].

**Figure S19**: QRA index from Temperature JJA seasonal means for CMIP5 Historical and RCP85.

**Figure S20**: QRA index from Temperature JJA seasonal means for CMIP6 Historical and SSP585.

**Figure S21**: QRA index from Temperature JJA seasonal means for CMIP6 Historical and SSP370.

**Figure S22**: QRA index from Temperature JJA seasonal means for CMIP6 Historical and SSP370-lowNTCF.

**Figure S23**: Average QRA index from Temperature JJA seasonal means for CMIP6 SSP370.

**Figure S24**: Average QRA index from Temperature JJA seasonal means for CMIP6 SSP370-lowNTCF.

**Figure S25**: Mean surface temperature trend patterns (JJA seasonal means) for CMIP6 SSP370. (A,D) multimodel ensemble, (B,E) most negative QRA-trending ensemble members, and (C,F) most positive QRA-trending ensemble members (“most” is defined as upper 10th percentile of multimodel ensemble).

**Figure S26**: Projection of QRA index from Temperature JJA seasonal means onto zonal wind anomalies for CMIP5 Historical.

**Figure S27**: Projection of QRA index from Temperature JJA seasonal means onto zonal wind anomalies for CMIP5 RCP85.

**Figure S28**: Projection of QRA index from Temperature JJA seasonal means onto zonal wind anomalies for CMIP6 Historical.

**Figure S29**: Projection of QRA index from Temperature JJA seasonal means onto zonal wind anomalies for CMIP6 SSP585.

**Figure S30**: Projection of QRA index from Temperature JJA seasonal means onto zonal wind anomalies for CMIP6 SSP370.

**Figure S31**: Temperature *T(φ)* for CMIP5 (1979-2005) Historical multimodel ensemble over 25N-75N (2.5 degrees) JJA seasonal means compared to observations.

**Figure S32**: Temperature *T(φ)* for CMIP6 (1979-2014) Historical multimodel ensemble over 25N-75N (2.5 degrees) JJA seasonal means compared to observations.

**Figure S33**: Temperature *dT(φ)/dφ* for CMIP5 (1979-2005) Historical multimodel ensemble over 25N-75N (2.5 degrees) JJA seasonal means compared to observations.

**Figure S34**: Temperature *dT(φ)/dφ* for CMIP6 (1979-2014) Historical multimodel ensemble over 25N-75N (2.5 degrees) JJA seasonal means compared to observations.

**Figure S35**: Zonal wind *ū(φ)* for CMIP5 (1979-2005) Historical multimodel ensemble over 25N-75N (2.5 degrees) JJA seasonal means compared to observations.

**Figure S36**: Zonal wind *ū(φ)* for CMIP6 (1979-2014) Historical multimodel ensemble over 25N-75N (2.5 degrees) JJA seasonal means compared to observations.

**Figure S37**: Zonal wind *dū(φ)/dφ* for CMIP5 (1979-2005) Historical multimodel ensemble over 25N-75N (2.5 degrees) JJA seasonal means compared to observations.

**Figure S38**: Zonal wind *dū(φ)/dφ* for CMIP6 (1979-2014) Historical multimodel ensemble over 25N-75N (2.5 degrees) JJA seasonal means compared to observations.

**Figure S39**: Zonal wind *d^2^ū(φ)/dφ^2^* for CMIP5 (1979-2005) Historical multimodel ensemble over 25N-75N (2.5 degrees) JJA seasonal means compared to observations.

**Figure S40**: Zonal wind *d^2^ū(φ)/dφ^2^* for CMIP6 (1979-2014) Historical multimodel ensemble over 25N-75N (2.5 degrees) JJA seasonal means compared to observations.

**Figure S41**: Temperature *T(φ)* RMSE for CMIP5 (1979-2005) Historical multimodel ensemble over 25N-75N (2.5 degrees) JJA seasonal means compared to ERA5 and ERA-Interim.

**Figure S42**: Temperature *T(φ)* RMSE for CMIP6 (1979-2014) Historical multimodel ensemble over 25N-75N (2.5 degrees) JJA seasonal means compared to ERA5 and ERA-Interim.

**Figure S43**: Temperature *dT(φ)/dφ* RMSE for CMIP5 (1979-2005) Historical multimodel ensemble over 25N-75N (2.5 degrees) JJA seasonal means compared to ERA5 and ERA-Interim.

**Figure S44**: Temperature *dT(φ)/dφ* RMSE for CMIP6 (1979-2014) Historical multimodel ensemble over 25N-75N (2.5 degrees) JJA seasonal means compared to ERA5 and ERA-Interim.

**Figure S45**: Zonal wind *ū(φ)* RMSE for CMIP5 (1979-2005) Historical multimodel ensemble over 25N-75N (2.5 degrees) JJA seasonal means compared to ERA5 and ERA-Interim.

**Figure S46**: Zonal wind *ū(φ)* RMSE for CMIP6 (1979-2014) Historical multimodel ensemble over 25N-75N (2.5 degrees) JJA seasonal means compared to ERA5 and ERA-Interim.

**Figure S47**: Zonal wind *dū(φ)/dφ* RMSE for CMIP5 (1979-2005) Historical multimodel ensemble over 25N-75N (2.5 degrees) JJA seasonal means compared to ERA5 and ERA-Interim.

**Figure S48**: Zonal wind *dū(φ)/dφ* RMSE for CMIP6 (1979-2014) Historical multimodel ensemble over 25N-75N (2.5 degrees) JJA seasonal means compared to ERA5 and ERA-Interim.

**Figure S49**: Zonal wind *d^2^ū(φ)/dφ^2^* RMSE for CMIP5 (1979-2005) Historical multimodel ensemble over 25N-75N (2.5 degrees) JJA seasonal means compared to ERA5 and ERA-Interim.

**Figure S50**: Zonal wind *d^2^ū(φ)/dφ^2^* RMSE for CMIP6 (1979-2014) Historical multimodel ensemble over 25N-75N (2.5 degrees) JJA seasonal means compared to ERA5 and ERA-Interim.

**
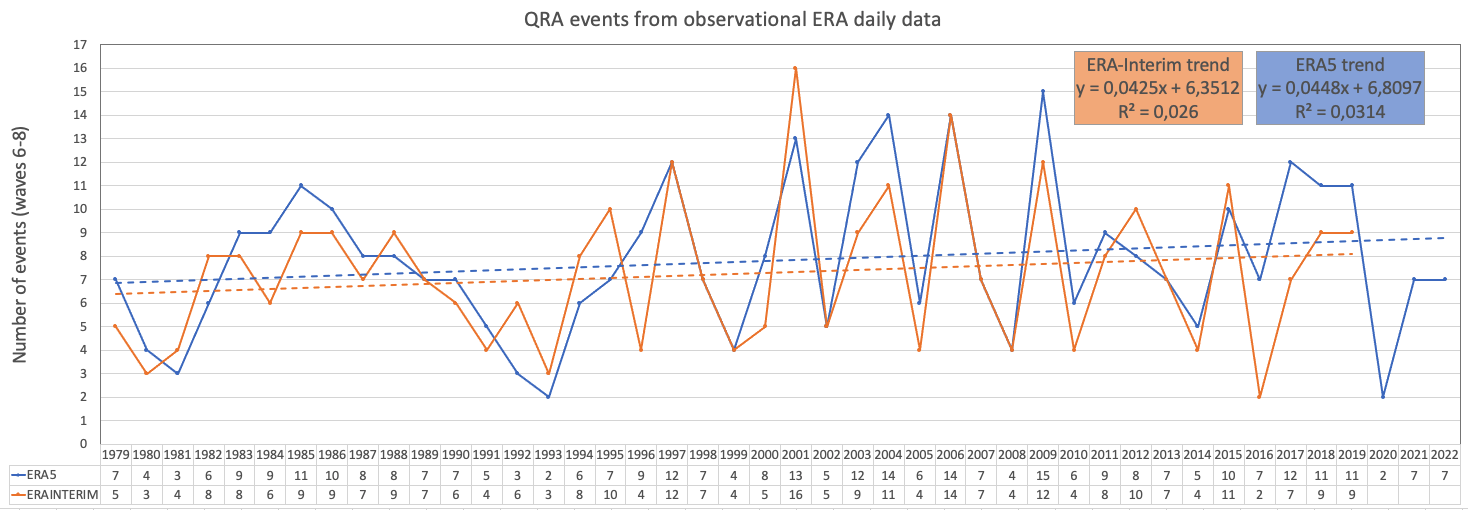
**

**Figure S1:** Number of QRA events for JJA wave numbers from 6 to 8 for the ERA5 and ERA-Interim reanalysis.

**Mann-Kendall Trend Test**

The Mann-Kendall Trend Test is a useful tool for identifying trends in time series data. Since it is non-parametric, no underlying assumption on the normality of the data is made ([1]).

If the ***p-value*** of the test is lower than some significance level (common choices are 0.10, 0.05, and 0.01), then there is statistically significant evidence that a trend is present in the time series data.

In our analysis, the trends were considered significant at significance level of 0.05.

Result for the ERA5 QRA events series (1979-2022) present in **Figure 1** and **Figure S1**:

***trend***='no trend', ***h***=False, ***p***=0.32732960607421324, ***z***=0.9795068491984874, ***Tau***=0.10253699788583509, ***s***=97.0, ***var_s***=9605.666666666666, ***slope***=0.031754032258064516, and ***intercept***=6.317288306451613.

Here is how to interpret the output of the test:

- ***trend***: This tells the trend. Possible output includes increasing, decreasing, or no trend.
- ***h***: True if trend is present. False if no trend is present.
- ***p***: The p-value of the test.
- ***z***: The normalized test statistic.
- ***Tau***: Kendall Tau.
- ***s***: Mann-Kendal’s score
- ***var_s***: Variance S
- ***slope***: Theil-Sen estimator/slope
- ***intercept***: Intercept of Kendall-Theil Robust Line

| **N** | **M** | **Model (realizations)** | **Variant label** | **Nominal resolution (km)** | **Atm levels** | **Components** | **Institution** | **Experiments in this study** |
| --- | --- | --- | --- | --- | --- | --- | --- | --- |
| 1 | 1 | ACCESS-CM2 (1) | r1i1p1f1 | 250 | 85 | AOGCM AER | CSIRO-ARCCSS | Historical SSP585 SSP370 |
| 2 | 2 | ACCESS-ESM1-5 (1) | r1i1p1f1 | 250 | 38 | AOGCM AER BGC | CSIRO | Historical SSP585 SSP370 |
| 3 | 3 | AWI-CM-1-1-MR (1) | r1i1p1f1 | 100 | 95 | AOGCM | AWI | Historical SSP585 |
| 4 | 4 | AWI-ESM-1-1-LR (1) | r1i1p1f1 | 250 | 47 | AOGCM | AWI | Historical |
| 5 | 5 | BCC-CSM2-MR (1) | r1i1p1f1 | 100 | 46 | AOGCM | BCC | Historical SSP585 SSP370 |
| 6 | 6 | BCC-ESM1 (1) | r1i1p1f1 | 250 | 26 | AOGCM AER CHEM | BCC | Historical |
| 7 | 7 | CAMS-CSM1-0 (1) | r1i1p1f1 | 100 | 31 | AOGCM | CAMS | Historical |
| 8 | 8 | CanESM5-CanOE (1) | r1i1p2f1 | 500 | 49 | AOGCM AER BGC | CCCma | Historical SSP585 |
| 9 | 9 | CanESM5 (3) | r1i1p1f1 | 500 | 49 | AOGCM AER BGC | CCCma | Historical SSP585 SSP370 |
| 10 |  |  | r1i1p2f1 |  |  |  |  | Historical SSP585 SSP370 |
| 11 |  |  | r2i1p1f1 |  |  |  |  | Historical SSP585 SSP370 |
| 12 | 10 | CAS-ESM2-0 (1) | r1i1p1f1 | 100 | 35 | AOGCM | CAS | Historical SSP585 |
| 13 | 11 | CESM2-FV2 (1) | r1i1p1f1 | 250 | 60 | AOGCM BGC CHEM AER | NCAR | Historical |
| 14 | 12 | CESM2 (1) | r1i1p1f1 | 100 | 60 | AOGCM BGC CHEM AER | NCAR | Historical SSP585 |
| 15 | 13 | CESM2-WACCM-FV2 (1) | r1i1p1f1 | 100 | 70 | AOGCM BGC CHEM AER | NCAR | Historical |
| 16 | 14 | CESM2-WACCM (1) | r1i1p1f1 | 100 | 70 | AOGCM BGC CHEM AER | NCAR | Historical SSP585 SSP370 |
| 17 | 15 | CMCC-CM2-HR4 (1) | r1i1p1f1 | 100 | 26 | AOGCM | CMCC | Historical |
| 18 | 16 | CMCC-CM2-SR5 (1) | r1i1p1f1 | 100 | 30 | AOGCM AER | CMCC | Historical SSP585 |
| 19 | 17 | CMCC-ESM2 (1) | r1i1p1f1 | 100 | 30 | AOGCM AER BGC | CMCC | Historical SSP585 |
| 20 | 18 | CNRM-CM6-1 (1) | r1i1p1f2 | 250 | 91 | AOGCM CHEM | CNRM-CERFACS | Historical SSP585 SSP370 |
| 21 | 19 | CNRM-ESM2-1 (1) | r1i1p1f2 | 250 | 91 | AOGCM BGC AER CHEM | CNRM-CERFACS | Historical SSP585 SSP370 |
| 22 | 20 | E3SM-1-0 (1) | r1i1p1f1 | 100 | 72 | AOGCM AER CHEM | E3SM-Project | Historical |
| 23 | 21 | E3SM-1-1-ECA (1) | r1i1p1f1 | 100 | 72 | AOGCM BGC AER CHEM | E3SM-Project | Historical |
| 24 | 22 | E3SM-1-1 (1) | r1i1p1f1 | 100 | 72 | AOGCM BGC AER CHEM | E3SM-Project | Historical SSP585 |
| 25 | 23 | EC-Earth3-AerChem (1) | r1i1p1f1 | 100 | 91 | AOGCM AER CHEM | EC-Earth-Consortium | Historical |
| 26 | 24 | EC-Earth3-CC (1) | r1i1p1f1 | 100 | 91 | AOGCM BGC | EC-Earth-Consortium | Historical SSP585 |
| 27 | 25 | EC-Earth3 (2) | r1i1p1f1 | 100 | 91 | AOGCM | EC-Earth-Consortium | Historical SSP585 SSP370 |
| 28 |  |  | r4i1p1f1 |  |  |  |  | Historical SSP585 SSP370 |
| 29 | 26 | EC-Earth3-Veg (1) | r1i1p1f1 | 100 | 91 | AOGCM | EC-Earth-Consortium | Historical SSP585 SSP370 |
| 30 | 27 | EC-Earth3-Veg-LR (1) | r1i1p1f1 | 250 | 62 | AOGCM | EC-Earth-Consortium | Historical SSP585 |
| 31 | 28 | FGOALS-f3-L (1) | r1i1p1f1 | 100 | 32 | AOGCM | CAS | Historical SSP585 SSP370 |
| 32 | 29 | FGOALS-g3 (1) | r1i1p1f1 | 250 | 26 | AOGCM | CAS | Historical SSP585 SSP370 |
| 33 | 30 | FIO-ESM-2-0 (1) | r1i1p1f1 | 100 | 26 | AOGCM BGC | FIO-QLNM | Historical SSP585 |
| 34 | 31 | GFDL-CM4 (1) | r1i1p1f1 | 100 | 33 | AOGCM AER CHEM BGC | NOAA-GFDL | Historical SSP585 |
| 35 | 32 | GFDL-ESM4 (1) | r1i1p1f1 | 100 | 49 | AOGCM AER CHEM BGC | NOAA-GFDL | Historical SSP585 SSP370 |
| 36 | 33 | GISS-E2-1-G-CC (1) | r1i1p1f1 | 250 | 40 | AOGCM AER CHEM BGC | NASA-GISS | Historical |
| 37 | 34 | GISS-E2-1-G (2) | r1i1p1f1 | 250 | 40 | AOGCM AER CHEM BGC | NASA-GISS | Historical |
| 38 |  |  | r1i1p3f1 |  |  | AOGCM AER CHEM BGC |  | Historical SSP585 |
| 39 | 35 | GISS-E2-1-H (1) | r1i1p1f1 | 250 | 40 | AOGCM AER CHEM BGC | NASA-GISS | Historical |
| 40 | 36 | HadGEM3-GC31-LL (1) | r1i1p1f3 | 250 | 85 | AOGCM AER | MOHC | Historical SSP585 |
| 41 | 37 | HadGEM3-GC31-MM (1) | r1i1p1f3 | 100 | 85 | AOGCM AER | MOHC | Historical SSP585 |
| 42 | 38 | IITM-ESM (1) | r1i1p1f1 | 250 | 64 | AOGCM BGC | CCCR-IITM | Historical |
| 43 | 39 | INM-CM4-8 (1) | r1i1p1f1 | 100 | 21 | AOGCM AER | INM | Historical SSP585 SSP370 |
| 44 | 40 | INM-CM5-0 (1) | r1i1p1f1 | 100 | 73 | AOGCM AER | INM | Historical SSP585 SSP370 |
| 45 | 41 | IPSL-CM5A2-INCA (1) | r1i1p1f1 | 500 | 39 | AOGCM BGC AER CHEM | IPSL | Historical |
| 46 | 42 | IPSL-CM6A-LR (1) | r1i1p1f1 | 250 | 75 | AOGCM BGC | IPSL | Historical SSP585 SSP370 |
| 47 | 43 | IPSL-CM6A-LR-INCA (1) | r1i1p1f1 | 250 | 75 | AOGCM BGC AER | IPSL | Historical |
| 48 | 44 | KACE-1-0-G (1) | r1i1p1f1 | 250 | 85 | AOGCM AER | NIMS-KMA | Historical SSP585 |
| 49 | 45 | KIOST-ESM (1) | r1i1p1f1 | 250 | 32 | AOGCM AER BGC | KIOST | Historical SSP585 |
| 50 | 46 | MCM-UA-1-0 (2) | r1i1p1f1 | 250 | 14 | AOGCM AER | UA | Historical |
| 51 | 47 |  | r1i1p1f2 |  |  |  |  | Historical SSP585 SSP370 |
| 52 | 48 | MIROC6 (1) | r1i1p1f1 | 250 | 81 | AOGCM AER BGC | MIROC | Historical SSP585 SSP370 |
| 53 | 49 | MIROC-ES2L (1) | r1i1p1f2 | 500 | 40 | AOGCM AER BGC | MIROC | Historical SSP585 SSP370 |
| 54 | 50 | MPI-ESM-1-2-HAM (1) | r1i1p1f1 | 250 | 47 | AOGCM AER CHEM BGC | HAMMOZ-Consortium | Historical |
| 55 | 51 | MPI-ESM1-2-HR (2) | r1i1p1f1 | 100 | 95 | AOGCM BGC | MPI-M | Historical SSP585 SSP370 |
| 56 |  |  | r2i1p1f1 |  |  |  |  | Historical SSP585 SSP370 |
| 57 | 52 | MPI-ESM1-2-LR (1) | r1i1p1f1 | 250 | 47 | AOGCM BGC | MPI-M | Historical SSP585 SSP370 |
| 58 | 53 | MRI-ESM2-0 (1) | r1i1p1f1 | 100 | 80 | AOGCM AER CHEM BGC | MRI | Historical SSP585 SSP370 |
| 59 | 54 | NESM3 (1) | r1i1p1f1 | 250 | 47 | AOGCM | NUIST | Historical SSP585 |
| 60 | 55 | NorCPM1 (1) | r1i1p1f1 | 250 | 26 | AOGCM AER BGC | NCC | Historical |
| 61 | 56 | NorESM2-LM (2) | r1i1p1f1 | 250 | 32 | AOGCM AER BGC CHEM | NCC | Historical SSP585 SSP370 |
| 62 |  |  | r2i1p1f1 |  |  | AOGCM AER BGC CHEM |  | Historical |
| 63 | 57 | NorESM2-MM (1) | r1i1p1f1 | 100 | 32 | AOGCM AER BGC CHEM | NCC | Historical SSP585 SSP370 |
| 64 | 58 | SAM0-UNICON (1) | r1i1p1f1 | 100 | 30 | AOGCM BGC AER | SNU | Historical |
| 65 | 59 | TaiESM1 (1) | r1i1p1f1 | 100 | 30 | AOGCM AER BGC | AS-RCEC | Historical SSP585 |
| 66 | 60 | UKESM1-0-LL (1) | r1i1p1f2 | 250 | 85 | AOGCM AER BGC CHEM | MOHC | Historical SSP585 SSP370 |

**Table S1**: CMIP6 multimodel ensemble. Additional information was obtained from [2] and [3].

| **N** | **M** | **Model (realizations)** | **Variant label** | **Nominal resolution (km)** | **Atm levels** | **Components** | **Institution** | **Experiments in this study** |
| --- | --- | --- | --- | --- | --- | --- | --- | --- |
| 1 | 1 | ACCESS1-0 (2) | r1i1p1 | 250 | 38 | AOGCM AER CHEM BGC | CSIRO-BOM | Historical RCP85 |
| 2 |  |  | r2i1p1 |  |  |  |  | Historical |
| 3 | 2 | ACCESS1.3 (3) | r1i1p1 | 250 | 38 | AOGCM AER | CSIRO-BOM | Historical RCP85 |
| 4 |  |  | r2i1p1 |  |  |  |  | Historical |
| 5 |  |  | r3i1p1 |  |  |  |  | Historical |
| 6 | 3 | bcc-csm1-1 (3) | r1i1p1 | 500 | 26 | AOGCM BGC | BCC | Historical RCP85 |
| 7 |  |  | r2i1p1 |  |  |  |  | Historical |
| 8 |  |  | r3i1p1 |  |  |  |  | Historical |
| 9 | 4 | bcc-csm1-1-m (3) | r1i1p1 | 100 | 26 | AOGCM BGC | BCC | Historical RCP85 |
| 10 |  |  | r2i1p1 |  |  |  |  | Historical |
| 11 |  |  | r3i1p1 |  |  |  |  | Historical |
| 12 | 5 | BNU-ESM (1) | r1i1p1 | 500 | 26 | AOGCM AER BGC | BNU | Historical RCP85 |
| 13 | 6 | CanESM2 (5) | r1i1p1 | 500 | 35 | AOGCM AER BGC | CCCma | Historical RCP85 |
| 14 |  |  | r2i1p1 |  |  |  |  | Historical RCP85 |
| 15 |  |  | r3i1p1 |  |  |  |  | Historical RCP85 |
| 16 |  |  | r4i1p1 |  |  |  |  | Historical RCP85 |
| 17 |  |  | r5i1p1 |  |  |  |  | Historical RCP85 |
| 18 | 7 | CCSM4 (8) | r1i1p1 | 100 | 27 | AOGCM AER CHEM BGC | NCAR | Historical RCP85 |
| 19 |  |  | r1i2p1 |  |  |  |  | Historical |
| 20 |  |  | r1i2p2 |  |  |  |  | Historical |
| 21 |  |  | r2i1p1 |  |  |  |  | Historical RCP85 |
| 22 |  |  | r3i1p1 |  |  |  |  | Historical RCP85 |
| 23 |  |  | r4i1p1 |  |  |  |  | Historical RCP85 |
| 24 |  |  | r5i1p1 |  |  |  |  | Historical RCP85 |
| 25 |  |  | r6i1p1 |  |  |  |  | Historical RCP85 |
| 26 | 8 | CESM1-BGC (1) | r1i1p1 | 100 | 27 | AOGCM AER BGC | NSF-DOE-NCAR | Historical RCP85 |
| 27 | 9 | CESM1-CAM5.1-FV2 (4) | r1i1p1 | 250 | 30 | AOGCM AER | NSF-DOE-NCAR | Historical |
| 28 |  |  | r2i1p1 |  |  |  |  | Historical |
| 29 |  |  | r3i1p1 |  |  |  |  | Historical |
| 30 |  |  | r4i1p1 |  |  |  |  | Historical |
| 31 | 10 | CESM1-CAM5 (2) | r1i1p1 | 100 | 27 | AOGCM AER | NSF-DOE-NCAR | Historical RCP85 |
| 32 |  |  | r2i1p1 |  |  |  |  | Historical RCP85 |
| 33 | 11 | CESM1-FASTCHEM (3) | r1i1p1 | 100 | 27 | AOGCM AER CHEM | NSF-DOE-NCAR | Historical |
| 34 |  |  | r2i1p1 |  |  |  |  | Historical |
| 35 |  |  | r3i1p1 |  |  |  |  | Historical |
| 36 | 12 | CESM1-WACCM (1) | r1i1p1 | 250 | 66 | AOGCM AER | NSF-DOE-NCAR | Historical |
| 37 | 13 | CMCC-CESM (1) | r1i1p1 | 500 | 39 | AOGCM AER BGC | CMCC | Historical RCP85 |
| 38 | 14 | CMCC-CM (1) | r1i1p1 | 100 | 31 | AOGCM AER | CMCC | Historical RCP85 |
| 39 | 15 | CMCC-CMS (1) | r1i1p1 | 250 | 95 | AOGCM AER | CMCC | Historical RCP85 |
| 40 | 16 | CNRM-CM5-2 (1) | r1i1p1 | 250 | 31 | AOGCM AER CHEM BGC | CNRM-CERFACS | Historical |
| 41 | 17 | CNRM-CM5 (10) | r1i1p1 | 250 | 31 | AOGCM CHEM BGC | CNRM-CERFACS | Historical RCP85 |
| 42 |  |  | r2i1p1 |  |  |  |  | Historical RCP85 |
| 43 |  |  | r3i1p1 |  |  |  |  | Historical |
| 44 |  |  | r4i1p1 |  |  |  |  | Historical RCP85 |
| 45 |  |  | r5i1p1 |  |  |  |  | Historical |
| 46 |  |  | r6i1p1 |  |  |  |  | Historical RCP85 |
| 47 |  |  | r7i1p1 |  |  |  |  | Historical |
| 48 |  |  | r8i1p1 |  |  |  |  | Historical |
| 49 |  |  | r9i1p1 |  |  |  |  | Historical |
| 50 |  |  | r10i1p1 |  |  |  |  | Historical RCP85 |
| 51 | 18 | CSIRO-Mk3-6-0 (10) | r1i1p1 | 250 | 18 | AOGCM AER | CSIRO-QCCCE | Historical RCP85 |
| 52 |  |  | r2i1p1 |  |  |  |  | Historical RCP85 |
| 53 |  |  | r3i1p1 |  |  |  |  | Historical RCP85 |
| 54 |  |  | r4i1p1 |  |  |  |  | Historical RCP85 |
| 55 |  |  | r5i1p1 |  |  |  |  | Historical RCP85 |
| 56 |  |  | r6i1p1 |  |  |  |  | Historical RCP85 |
| 57 |  |  | r7i1p1 |  |  |  |  | Historical RCP85 |
| 58 |  |  | r8i1p1 |  |  |  |  | Historical RCP85 |
| 59 |  |  | r9i1p1 |  |  |  |  | Historical RCP85 |
| 60 |  |  | r10i1p1 |  |  |  |  | Historical RCP85 |

**Table S2**: CMIP5 multimodel ensemble. Additional information was obtained from [2] and [3].

| **N** | **M** | **Model (realizations)** | **Variant label** | **Nominal resolution (km)** | **Atm levels** | **Components** | **Institution** | **Experiments in this study** |
| --- | --- | --- | --- | --- | --- | --- | --- | --- |
| 61 | 19 | EC-EARTH (9) | r1i1p1 | 100 | 62 | AOGCM | ICHEC | Historical RCP85 |
| 62 |  |  | r2i1p1 |  |  |  |  | Historical RCP85 |
| 63 |  |  | r7i1p1 |  |  |  |  | Historical RCP85 |
| 64 |  |  | r8i1p1 |  |  |  |  | Historical |
| 65 |  |  | r9i1p1 |  |  |  |  | Historical RCP85 |
| 66 |  |  | r11i1p1 |  |  |  |  | Historical RCP85 |
| 67 |  |  | r12i1p1 |  |  |  |  | Historical |
| 68 |  |  | r13i1p1 |  |  |  |  | Historical RCP85 |
| 69 |  |  | r14i1p1 |  |  |  |  | Historical RCP85 |
| 70 | 20 | FGOALS_g2 (4) | r1i1p1 | 500 | 26 | AOGCM AER | LASG-CESS | Historical RCP85 |
| 71 |  |  | r3i1p1 |  |  |  |  | Historical |
| 72 |  |  | r4i1p1 |  |  |  |  | Historical |
| 73 |  |  | r5i1p1 |  |  |  |  | Historical |
| 74 | 21 | FIO-ESM (3) | r1i1p1 | 500 | 26 | AOGCM BGC | FIO | Historical RCP85 |
| 75 |  |  | r2i1p1 |  |  |  |  | Historical RCP85 |
| 76 |  |  | r3i1p1 |  |  |  |  | Historical RCP85 |
| 77 | 22 | GISS-E2-H-CC (1) | r1i1p1 | 250 | 40 | AOGCM BGC | NASA-GISS | Historical RCP85 |
| 78 | 23 | GISS-E2-H (17) | r1i1p1 | 250 | 40 | AOGCM AER CHEM | NASA-GISS | Historical RCP85 |
| 79 |  |  | r1i1p2 |  |  |  |  | Historical RCP85 |
| 80 |  |  | r1i1p3 |  |  |  |  | Historical RCP85 |
| 81 |  |  | r2i1p1 |  |  |  |  | Historical |
| 82 |  |  | r2i1p2 |  |  |  |  | Historical |
| 83 |  |  | r2i1p3 |  |  |  |  | Historical |
| 84 |  |  | r3i1p1 |  |  |  |  | Historical |
| 85 |  |  | r3i1p2 |  |  |  |  | Historical |
| 86 |  |  | r3i1p3 |  |  |  |  | Historical |
| 87 |  |  | r4i1p1 |  |  |  |  | Historical |
| 88 |  |  | r4i1p2 |  |  |  |  | Historical |
| 89 |  |  | r4i1p3 |  |  |  |  | Historical |
| 90 |  |  | r5i1p1 |  |  |  |  | Historical |
| 91 |  |  | r5i1p2 |  |  |  |  | Historical |
| 92 |  |  | r5i1p3 |  |  |  |  | Historical |
| 93 |  |  | r6i1p1 |  |  |  |  | Historical |
| 94 |  |  | r6i1p3 |  |  |  |  | Historical |
| 95 | 24 | GISS-E2-R-CC (1) | r1i1p1 | 250 | 40 | AOGCM BGC | NASA-GISS | Historical RCP85 |
| 96 | 25 | GISS-E2-R (18) | r1i1p1 | 250 | 40 | AOGCM AER CHEM | NASA-GISS | Historical RCP85 |
| 97 |  |  | r1i1p2 |  |  |  |  | Historical RCP85 |
| 98 |  |  | r1i1p3 |  |  |  |  | Historical RCP85 |
| 99 |  |  | r2i1p1 |  |  |  |  | Historical |
| 100 |  |  | r2i1p2 |  |  |  |  | Historical |
| 101 |  |  | r2i1p3 |  |  |  |  | Historical |
| 102 |  |  | r3i1p1 |  |  |  |  | Historical |
| 103 |  |  | r3i1p2 |  |  |  |  | Historical |
| 104 |  |  | r3i1p3 |  |  |  |  | Historical |
| 105 |  |  | r4i1p1 |  |  |  |  | Historical |
| 106 |  |  | r4i1p2 |  |  |  |  | Historical |
| 107 |  |  | r4i1p3 |  |  |  |  | Historical |
| 108 |  |  | r5i1p1 |  |  |  |  | Historical |
| 109 |  |  | r5i1p2 |  |  |  |  | Historical |
| 110 |  |  | r5i1p3 |  |  |  |  | Historical |
| 111 |  |  | r6i1p1 |  |  |  |  | Historical |
| 112 |  |  | r6i1p2 |  |  |  |  | Historical |
| 113 |  |  | r6i1p3 |  |  |  |  | Historical |
| 114 | 26 | inmcm4 (1) | r1i1p1 | 250 | 21 | AOGCM BGC | INM | Historical RCP85 |
| 115 | 27 | IPSL-CM5A-LR (6) | r1i1p1 | 500 | 39 | AOGCM AER BGC | IPSL | Historical RCP85 |
| 116 |  |  | r2i1p1 |  |  |  |  | Historical RCP85 |
| 117 |  |  | r3i1p1 |  |  |  |  | Historical RCP85 |
| 118 |  |  | r4i1p1 |  |  |  |  | Historical RCP85 |
| 119 |  |  | r5i1p1 |  |  |  |  | Historical |
| 120 |  |  | r6i1p1 |  |  |  |  | Historical |

**Table S3**: CMIP5 multimodel ensemble (continuation). Additional information was obtained from [2] and [3].

| **N** | **M** | **Model (realizations)** | **Variant label** | **Nominal resolution (km)** | **Atm levels** | **Components** | **Institution** | **Experiments in this study** |
| --- | --- | --- | --- | --- | --- | --- | --- | --- |
| 121 | 28 | IPSL-CM5A-MR (3) | r1i1p1 | 250 | 39 | AOGCM AER BGC | IPSL | Historical RCP85 |
| 122 |  |  | r2i1p1 |  |  |  |  | Historical |
| 123 |  |  | r3i1p1 |  |  |  |  | Historical |
| 124 | 29 | IPSL-CM5B-LR (1) | r1i1p1 | 500 | 39 | AOGCM AER BGC | IPSL | Historical RCP85 |
| 125 | 30 | MIROC5 (5) | r1i1p1 | 250 | 40 | AOGCM AER | MIROC | Historical RCP85 |
| 126 |  |  | r2i1p1 |  |  |  |  | Historical RCP85 |
| 127 |  |  | r3i1p1 |  |  |  |  | Historical RCP85 |
| 128 |  |  | r4i1p1 |  |  |  |  | Historical |
| 129 |  |  | r5i1p1 |  |  |  |  | Historical |
| 130 | 31 | MIROC-ESM-CHEM (1) | r1i1p1 | 500 | 80 | AOGCM AER CHEM BGC | MIROC | Historical RCP85 |
| 131 | 32 | MIROC-ESM (3) | r1i1p1 | 500 | 38 | AOGCM AER BGC | MIROC | Historical RCP85 |
| 132 |  |  | r2i1p1 |  |  |  |  | Historical |
| 133 |  |  | r3i1p1 |  |  |  |  | Historical |
| 134 | 33 | MPI-ESM-LR (3) | r1i1p1 | 250 | 47 | AOGCM BGC | MPI-M | Historical RCP85 |
| 135 |  |  | r2i1p1 |  |  |  |  | Historical RCP85 |
| 136 |  |  | r3i1p1 |  |  |  |  | Historical RCP85 |
| 137 | 34 | MPI-ESM-MR (3) | r1i1p1 | 250 | 95 | AOGCM BGC | MPI-M | Historical RCP85 |
| 138 |  |  | r2i1p1 |  |  |  |  | Historical |
| 139 |  |  | r3i1p1 |  |  |  |  | Historical |
| 140 | 35 | MPI-ESM-P (2) | r1i1p1 | 250 | 47 | AOGCM BGC | MPI-M | Historical |
| 141 |  |  | r2i1p1 |  |  |  |  | Historical |
| 142 | 36 | MRI-CGCM3 (5) | r1i1p1 | 100 | 48 | AOGCM AER | MRI | Historical RCP85 |
| 143 |  |  | r2i1p1 |  |  |  |  | Historical |
| 144 |  |  | r3i1p1 |  |  |  |  | Historical |
| 145 |  |  | r4i1p2 |  |  |  |  | Historical |
| 146 |  |  | r5i1p2 |  |  |  |  | Historical |
| 147 | 37 | NorESM1-ME (1) | r1i1p1 | 250 | 26 | AOGCM AER CHEM BGC | NCC | Historical RCP85 |
| 148 | 38 | NorESM1-M (3) | r1i1p1 | 250 | 26 | AOGCM AER CHEM | NCC | Historical RCP85 |
| 149 |  |  | r2i1p1 |  |  |  |  | Historical |
| 150 |  |  | r3i1p1 |  |  |  |  | Historical |

**Table S4**: CMIP5 multimodel ensemble (continuation). Additional information was obtained from [2] and [3].

**
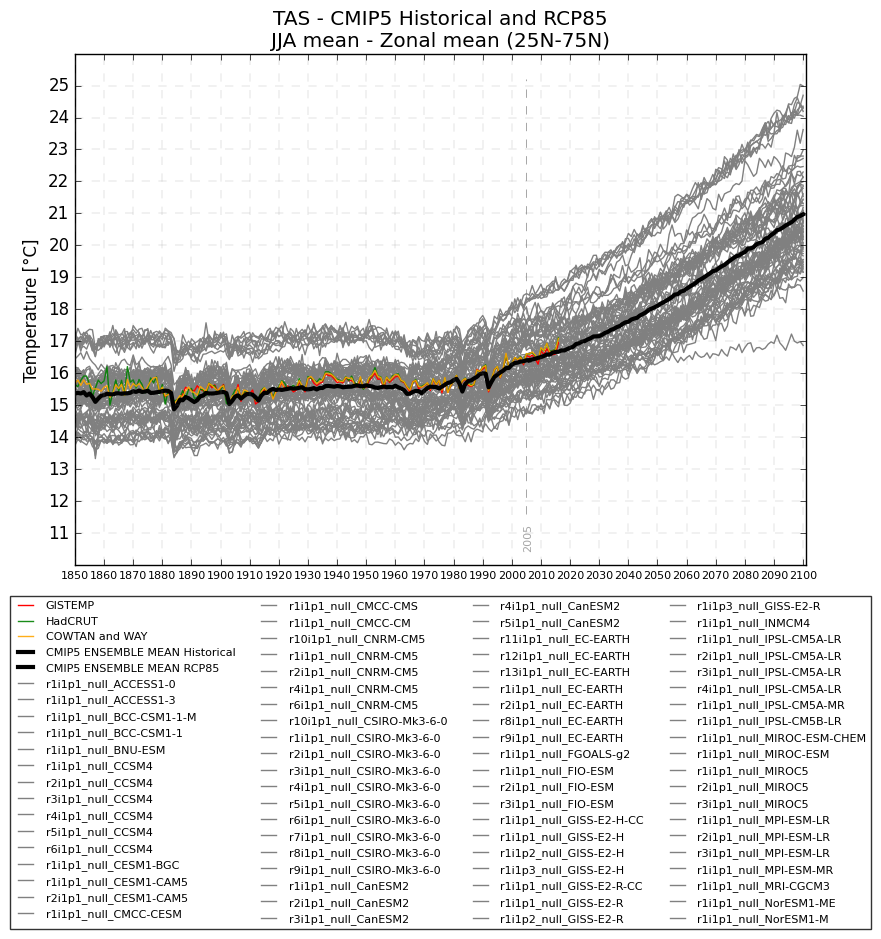
**

**Figure S2:** Temperature JJA seasonal means for CMIP5 Historical and RCP85 simulations.

**
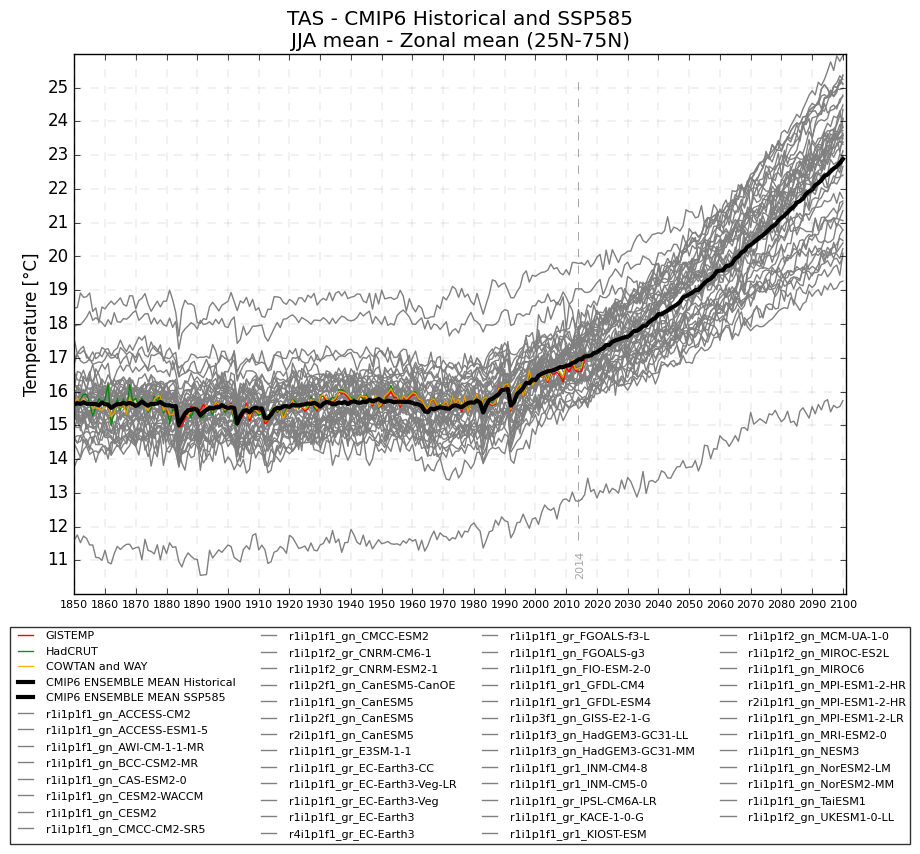
**

**Figure S3:** Temperature JJA seasonal means for CMIP6 Historical and SSP585 simulations.

**
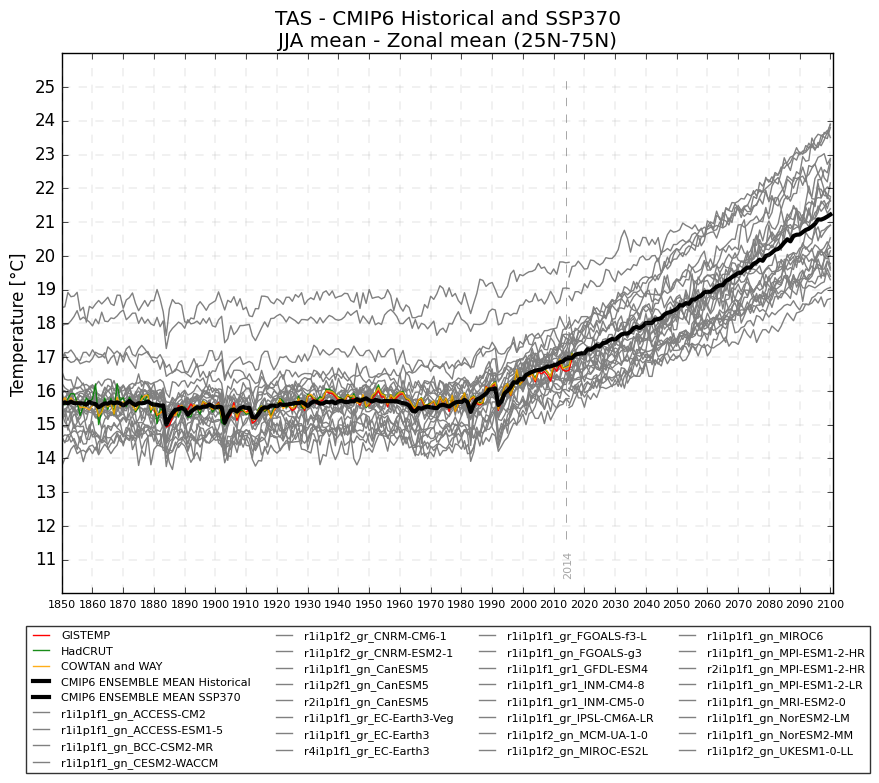
**

**Figure S4:** Temperature JJA seasonal means for CMIP6 Historical and SSP370 simulations.

**
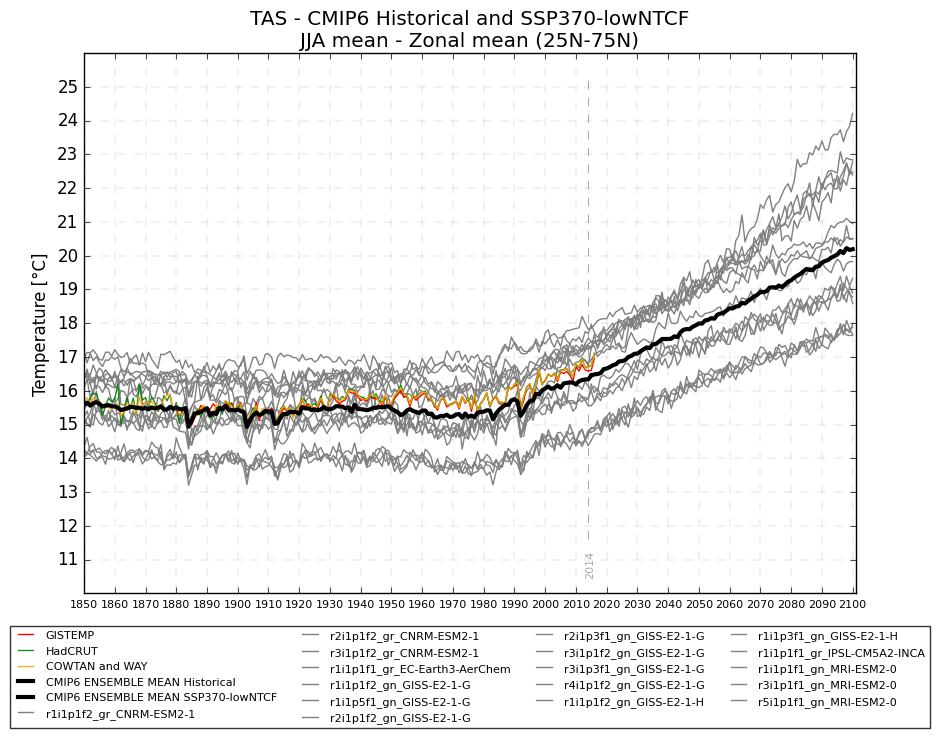
**

**Figure S5:** Temperature JJA seasonal means for CMIP6 Historical and SSP370-lowNTCF simulations.

**
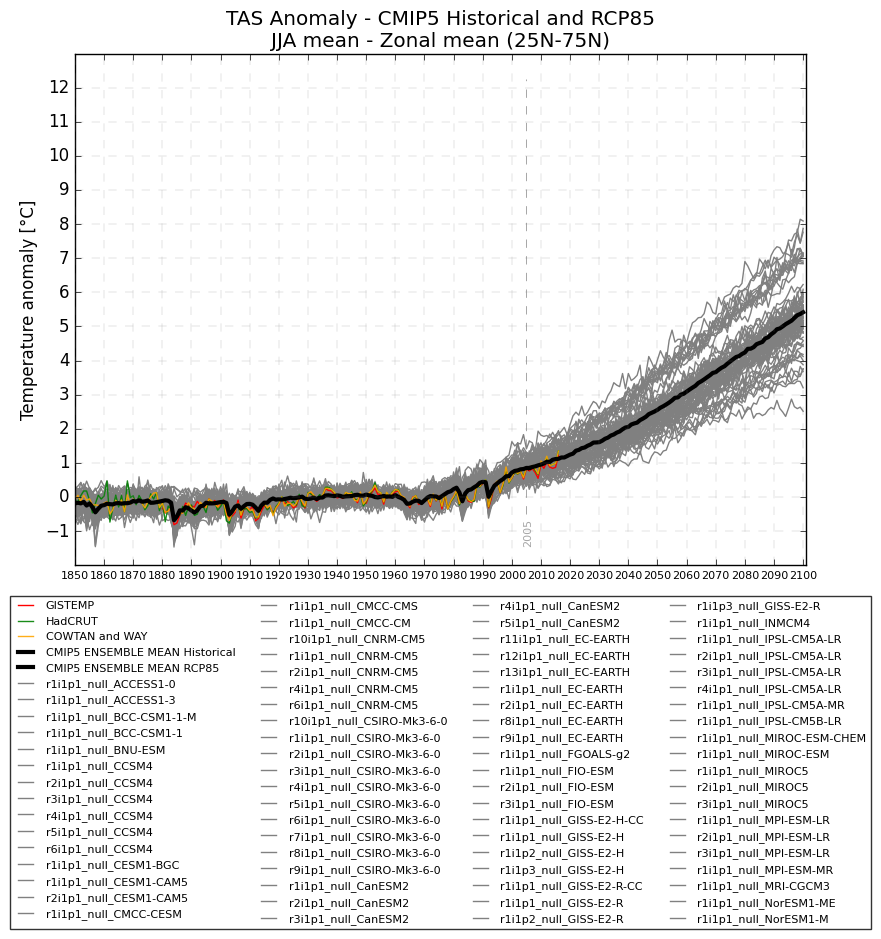
**

**Figure S6:** Temperature anomaly JJA seasonal means for CMIP5 Historical and RCP85 simulations. This anomaly follows [4].

**
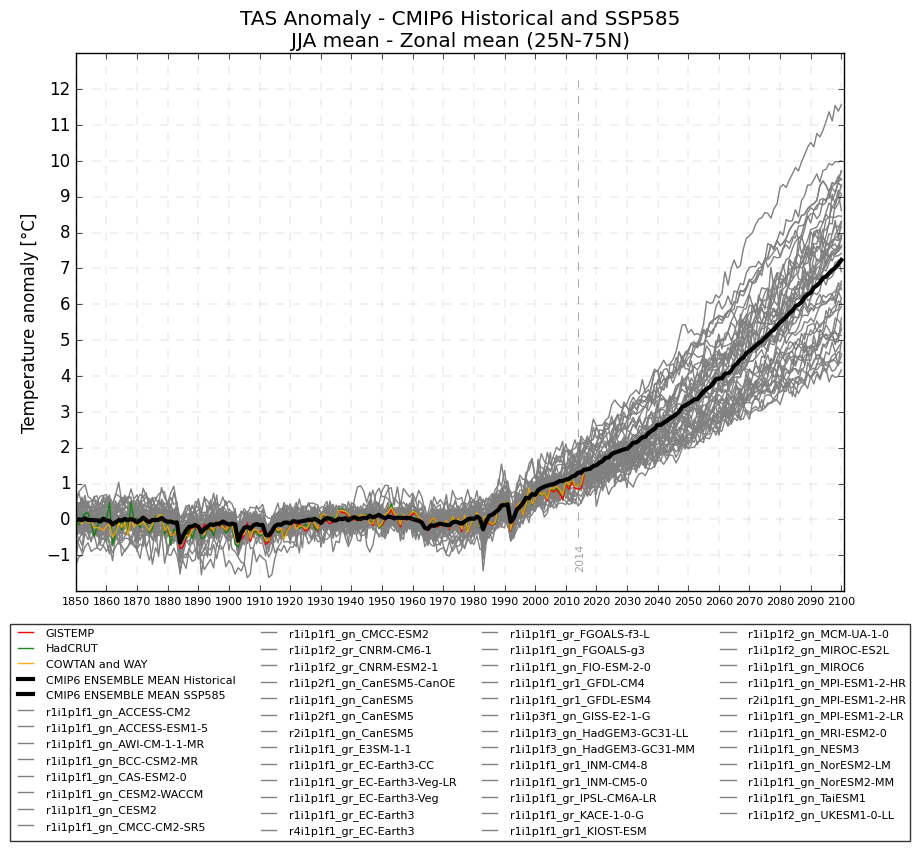
**

**Figure S7:** Temperature anomaly JJA seasonal means for CMIP6 Historical and SSP585 simulations. This anomaly follows [4].

**
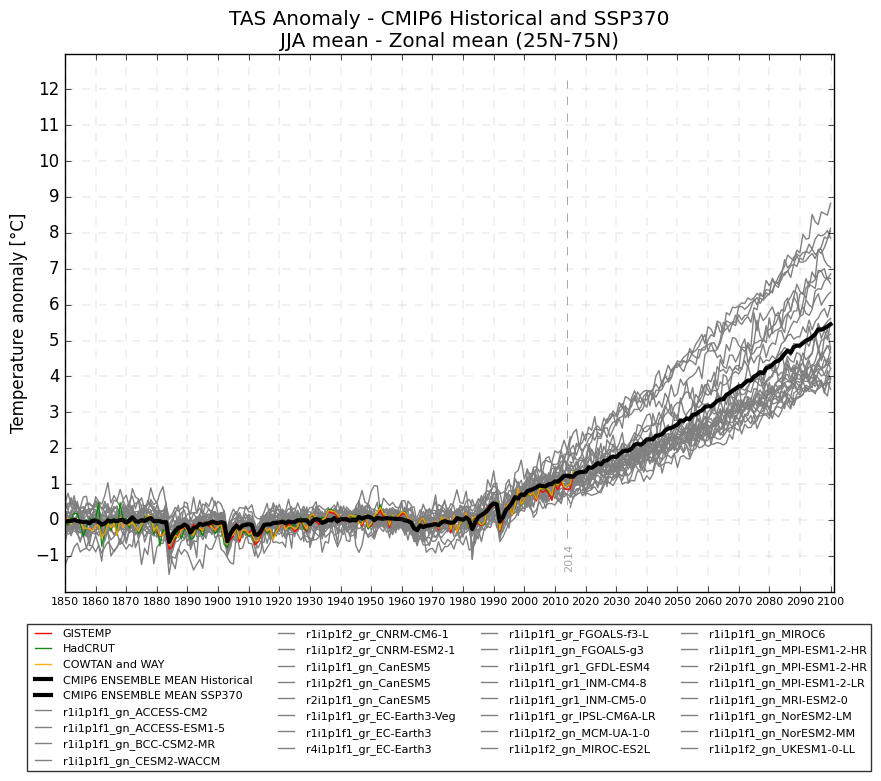
**

**Figure S8:** Temperature anomaly JJA seasonal means for CMIP6 Historical and SSP370 simulations. This anomaly follows [4].

**
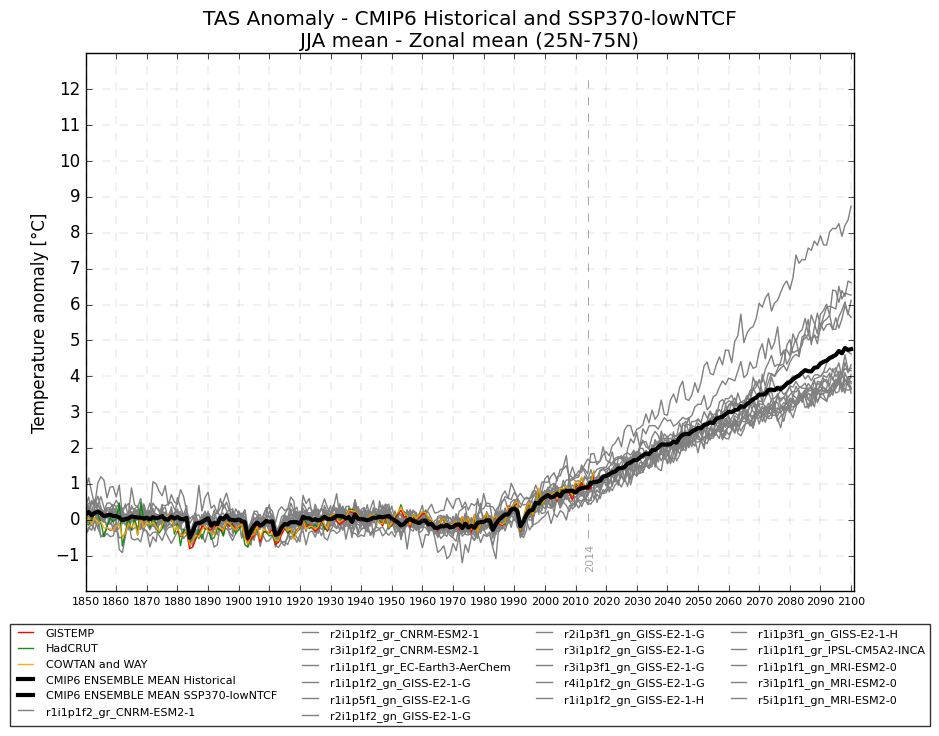
**

**Figure S9:** Temperature anomaly JJA seasonal means for CMIP6 Historical and SSP370-lowNTCF simulations. This anomaly follows [4].

**
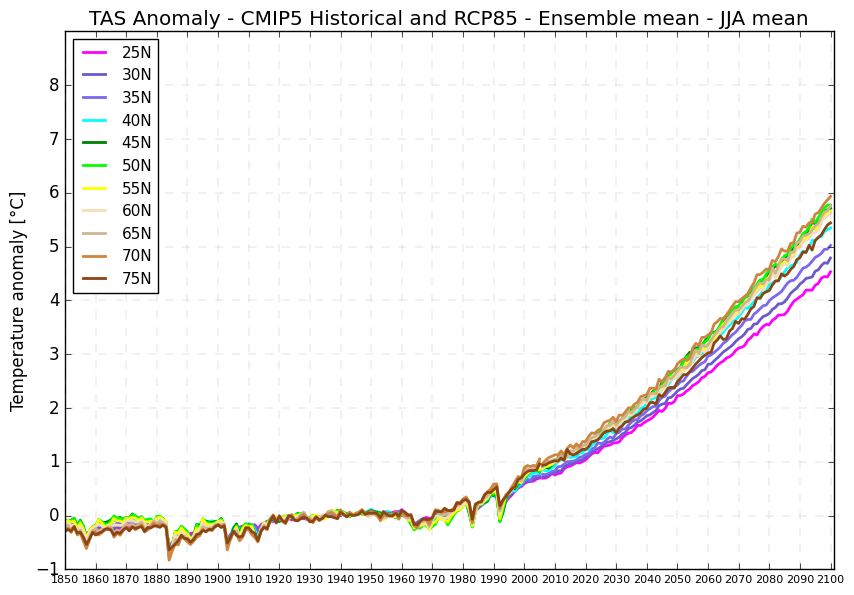
**

**Figure S10:** Temperature anomaly JJA seasonal means for CMIP5 Historical and RCP85. This anomaly follows [4].

**
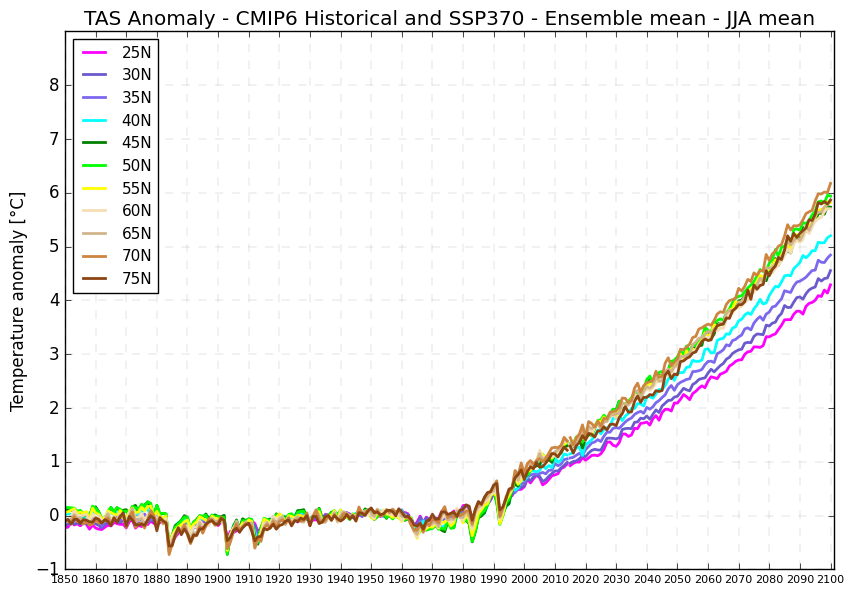
**

**Figure S11:** Temperature anomaly JJA seasonal means for CMIP6 Historical and SSP370. This anomaly follows [4].

**
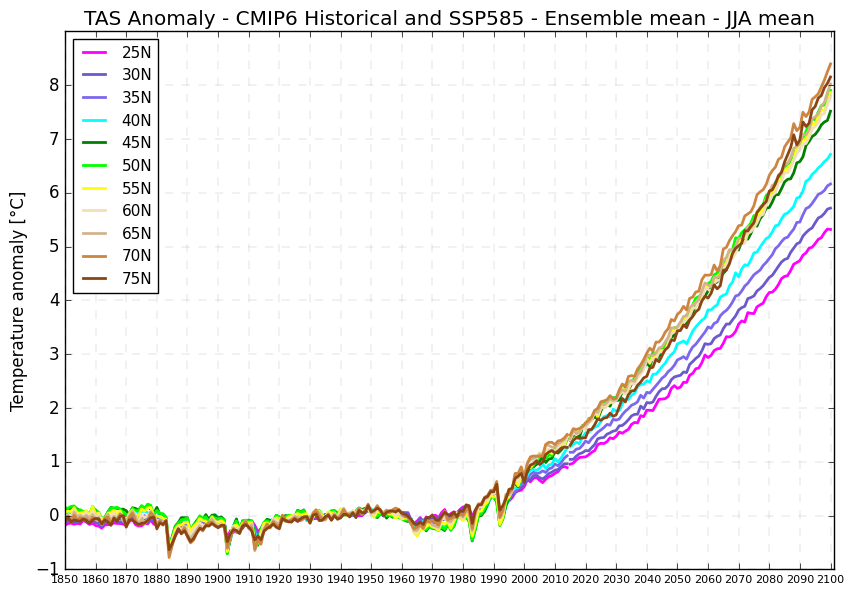
**

**Figure S12:** Temperature anomaly JJA seasonal means for CMIP6 Historical and SSP585. This anomaly follows [4].

**
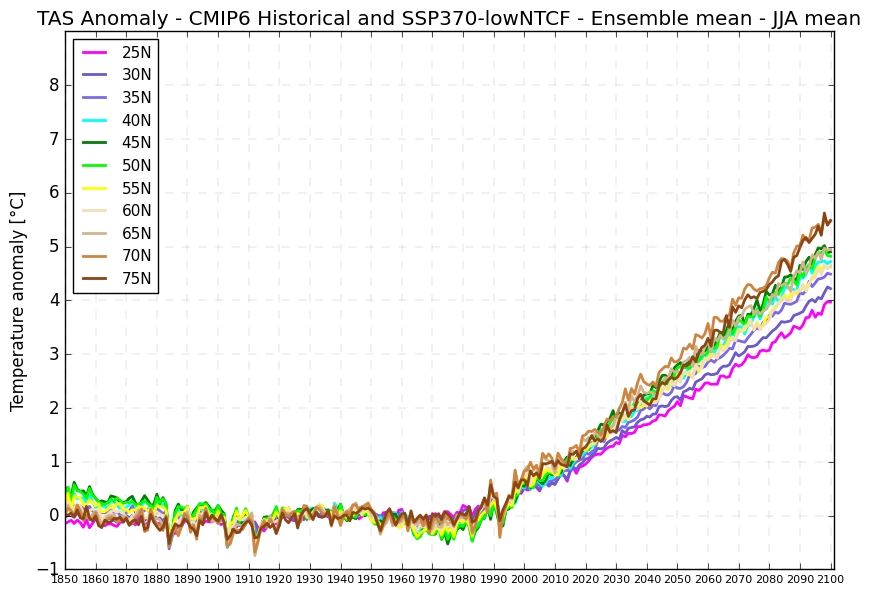
**

**Figure S13:** Temperature anomaly JJA seasonal means for CMIP6 Historical and SSP370-lowNTCF. This anomaly follows [4].

**
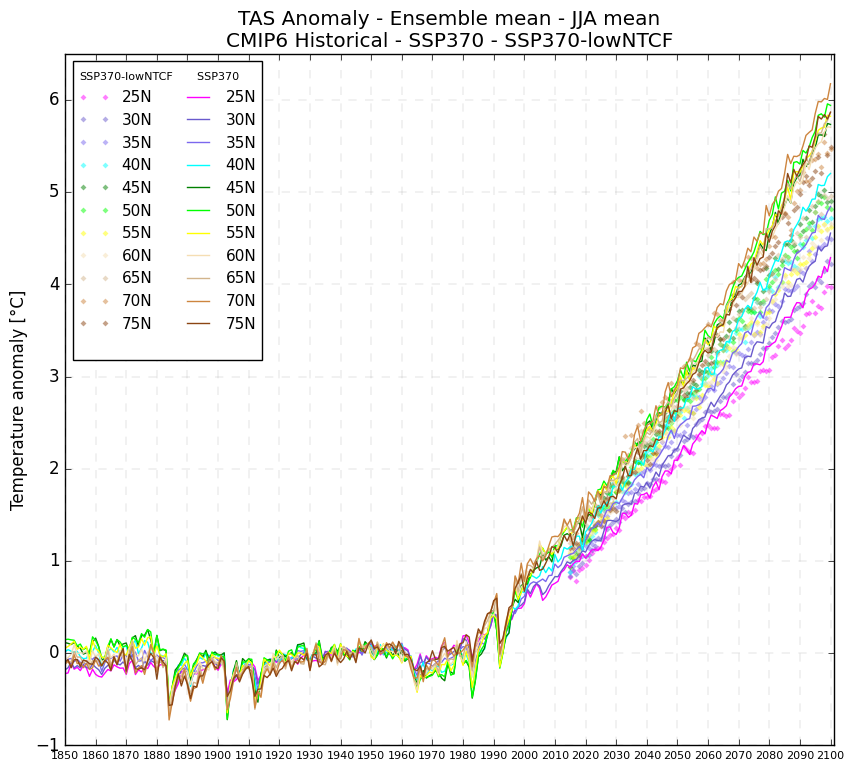
**

**Figure S14:** Temperature anomaly JJA seasonal means for CMIP6 Historical, SSP370, and SSP370-lowNTCF. This anomaly follows [4].

**
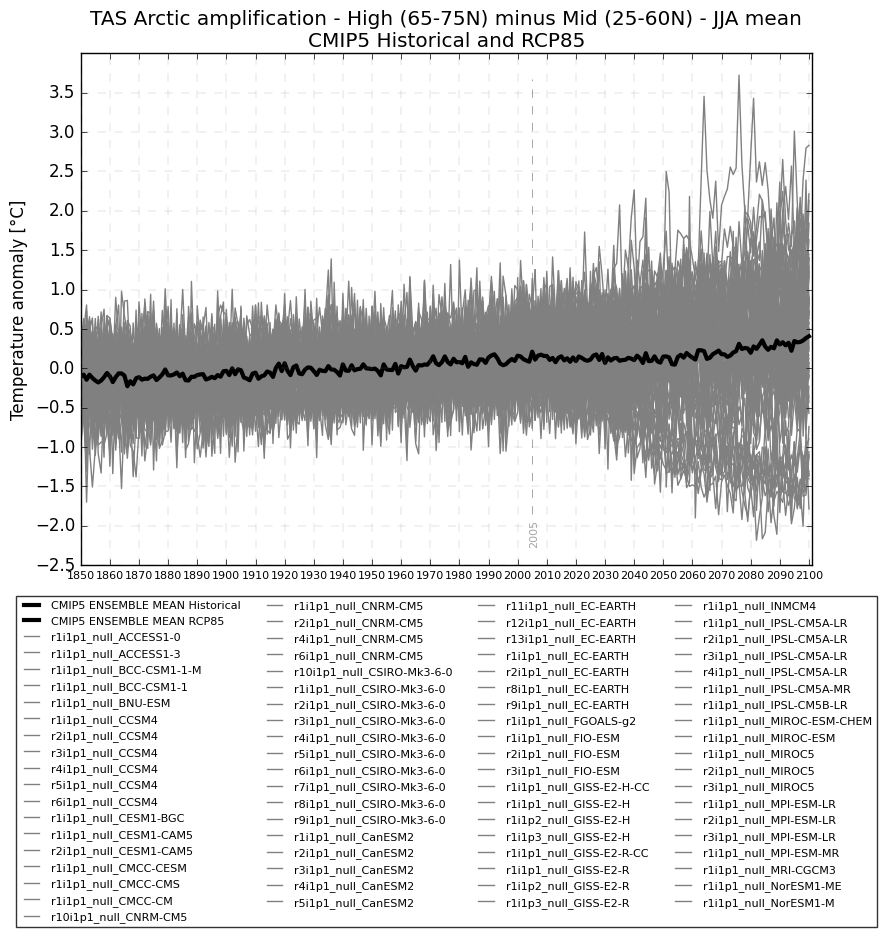
**

**Figure S15:** Arctic amplification from Temperature anomaly JJA seasonal means for CMIP5 Historical and RCP85. This anomaly follows [4].

**
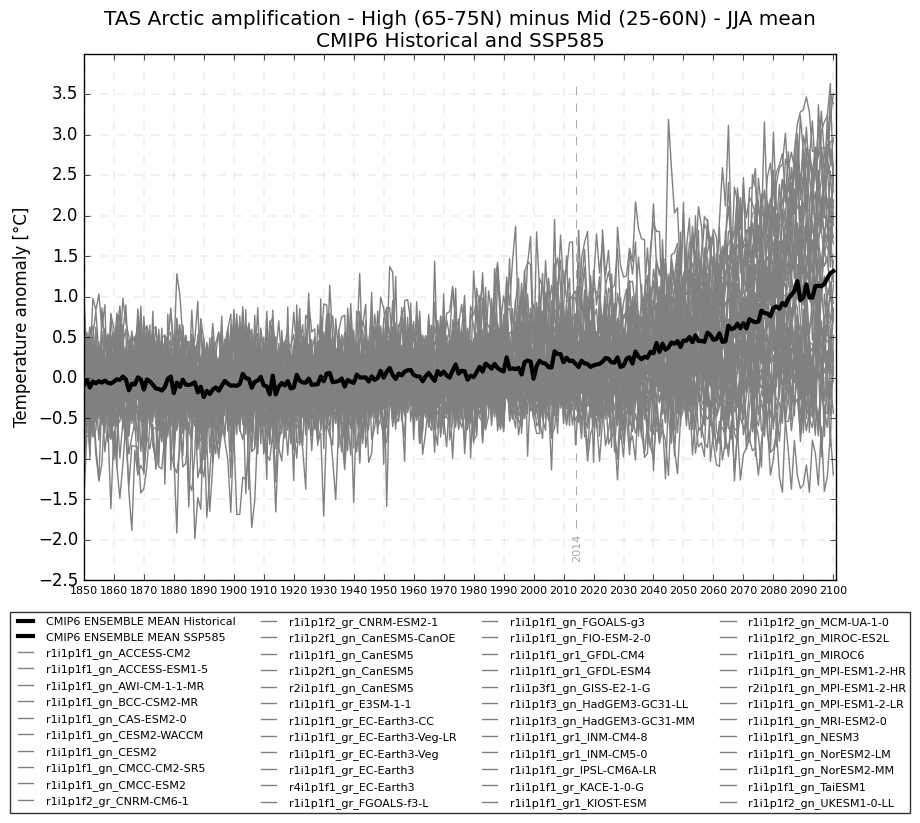
**

**Figure S16:** Arctic amplification from Temperature anomaly JJA seasonal means for CMIP6 Historical and SSP585. This anomaly follows [4].

**
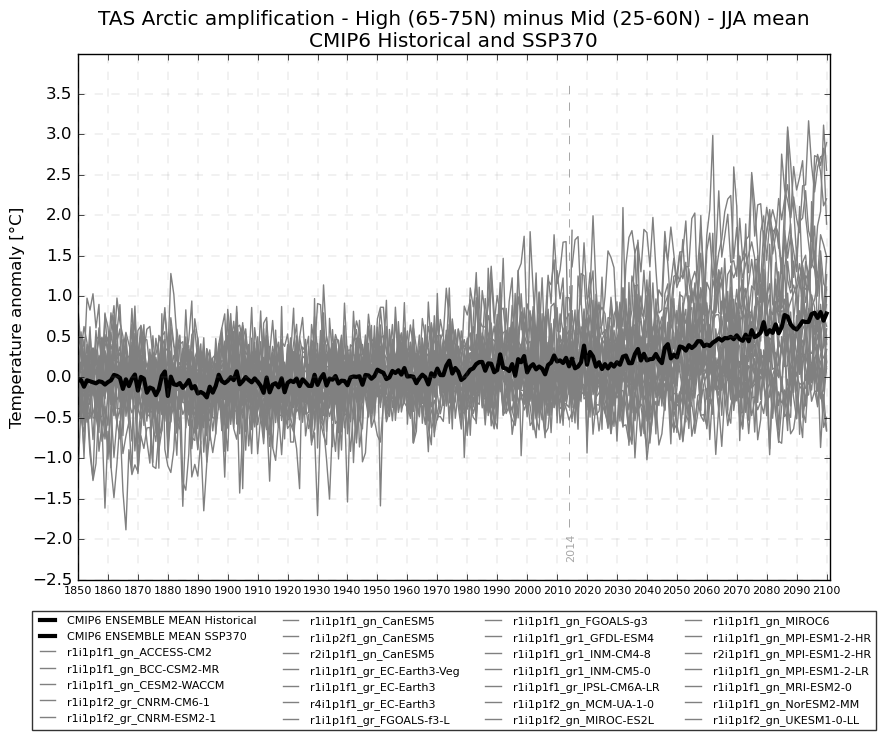
**

**Figure S17:** Arctic amplification from Temperature anomaly JJA seasonal means for CMIP6 Historical and SSP370. This anomaly follows [4].

**
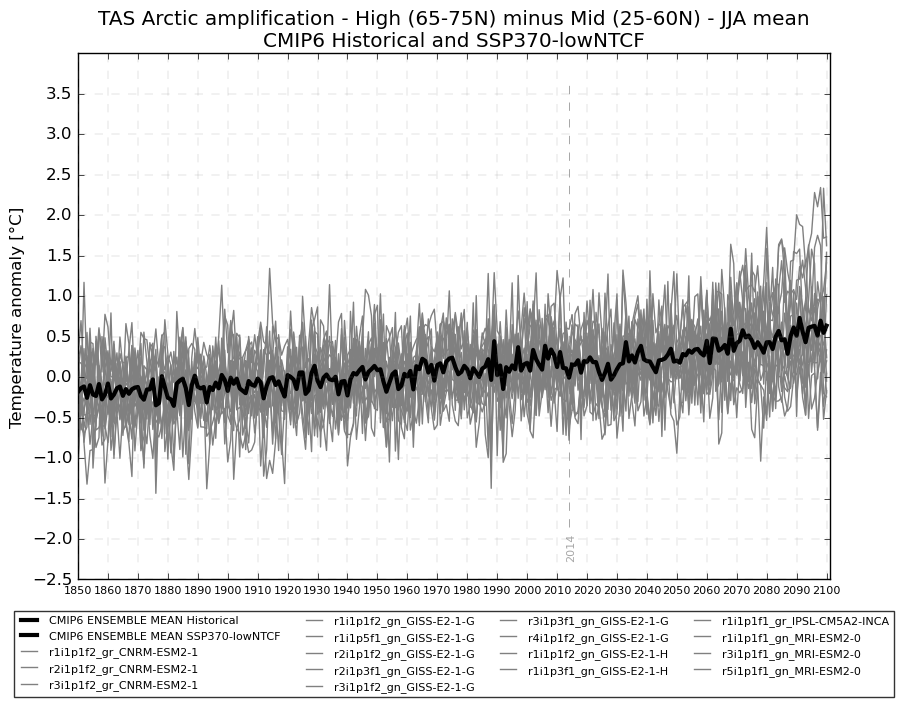
**

**Figure S18:** Arctic amplification from Temperature anomaly JJA seasonal means for CMIP6 Historical and SSP370-lowNTCF. This anomaly follows [4].

**
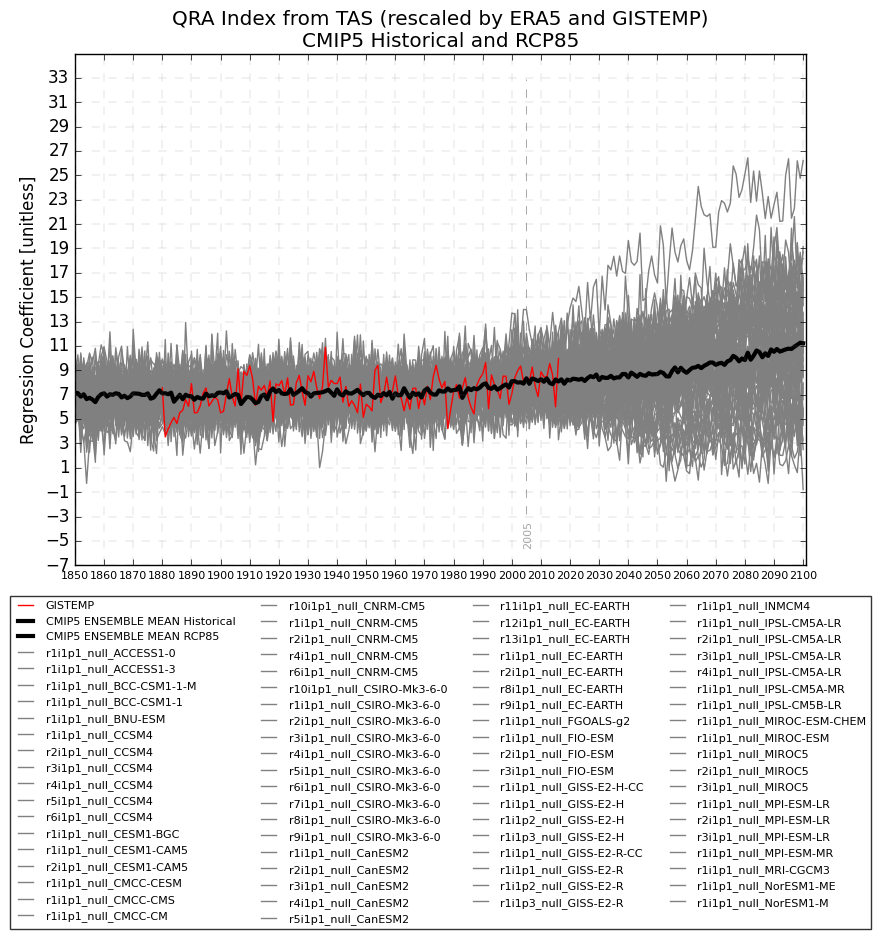
**

**Figure S19:** QRA index from Temperature JJA seasonal means for CMIP5 Historical and RCP85.

**
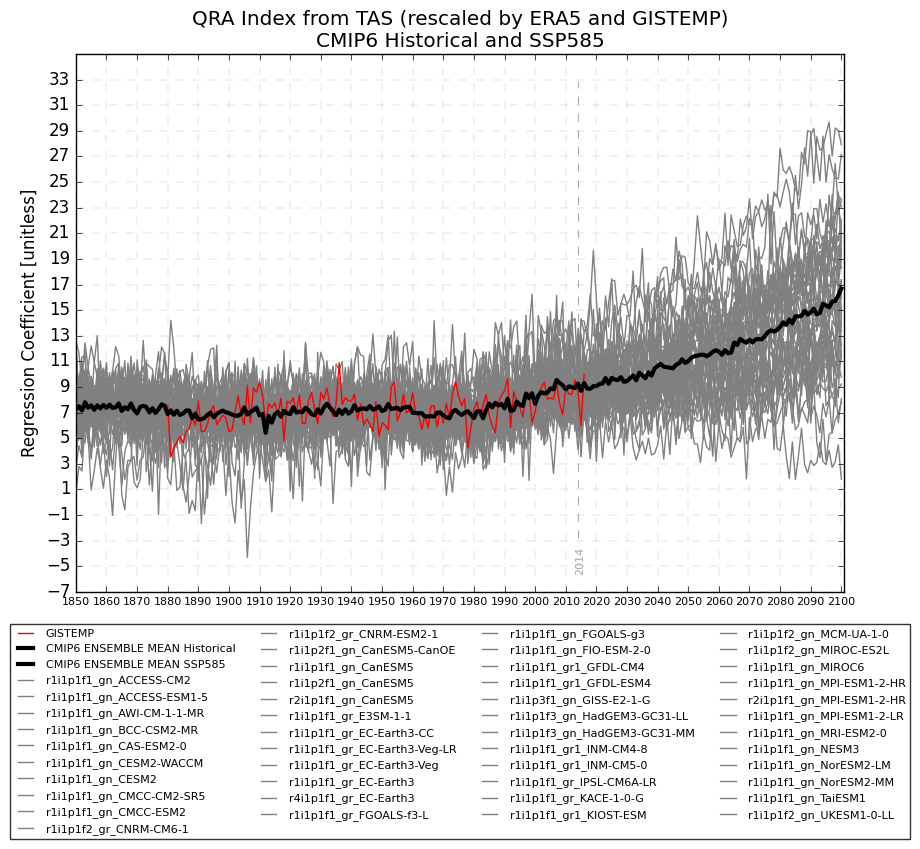
**

**Figure S20:** QRA index from Temperature JJA seasonal means for CMIP6 Historical and SSP585.

**
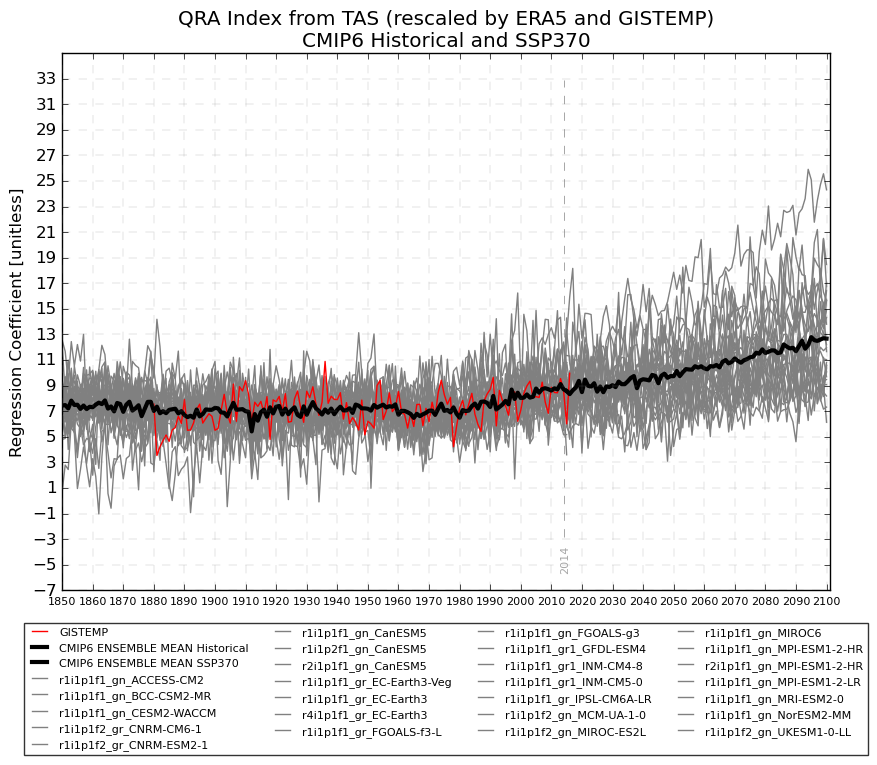
**

**Figure S21:** QRA index from Temperature JJA seasonal means for CMIP6 Historical and SSP370.

**
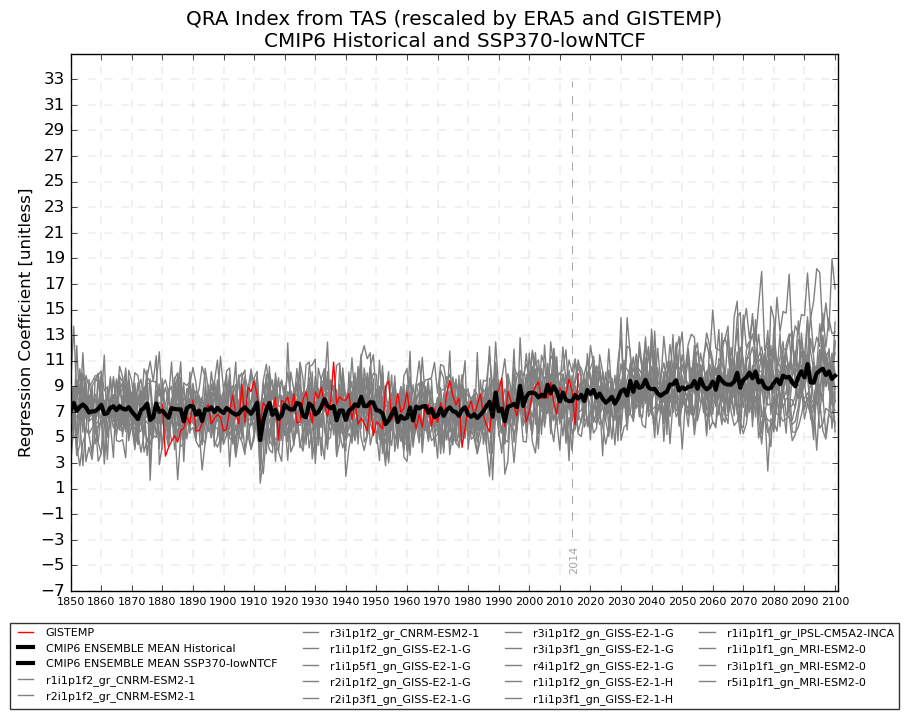
**

**Figure S22:** QRA index from Temperature JJA seasonal means for CMIP6 Historical and SSP370-lowNTCF.

**
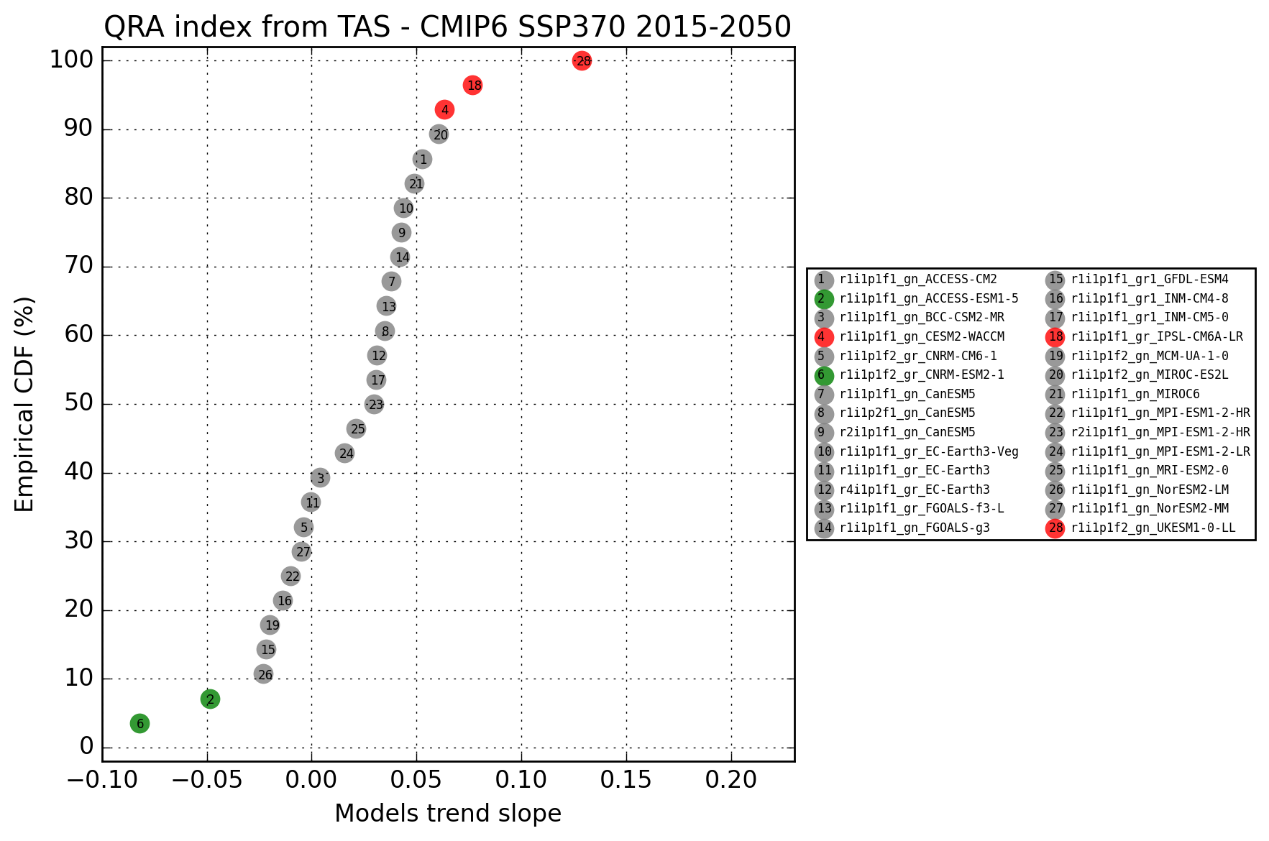
**

**
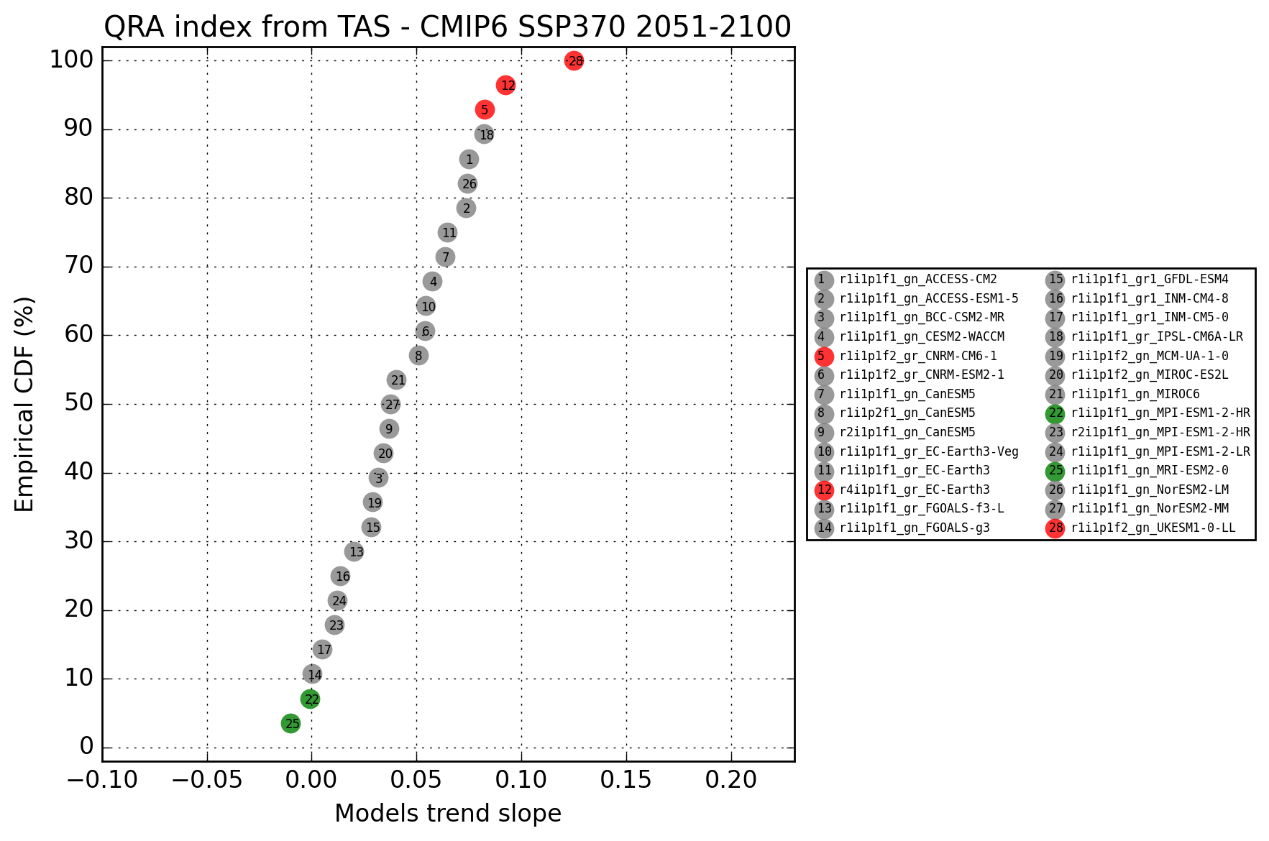
**

**Figure S23:** Average QRA index from Temperature JJA seasonal means for CMIP6 SSP370.

**
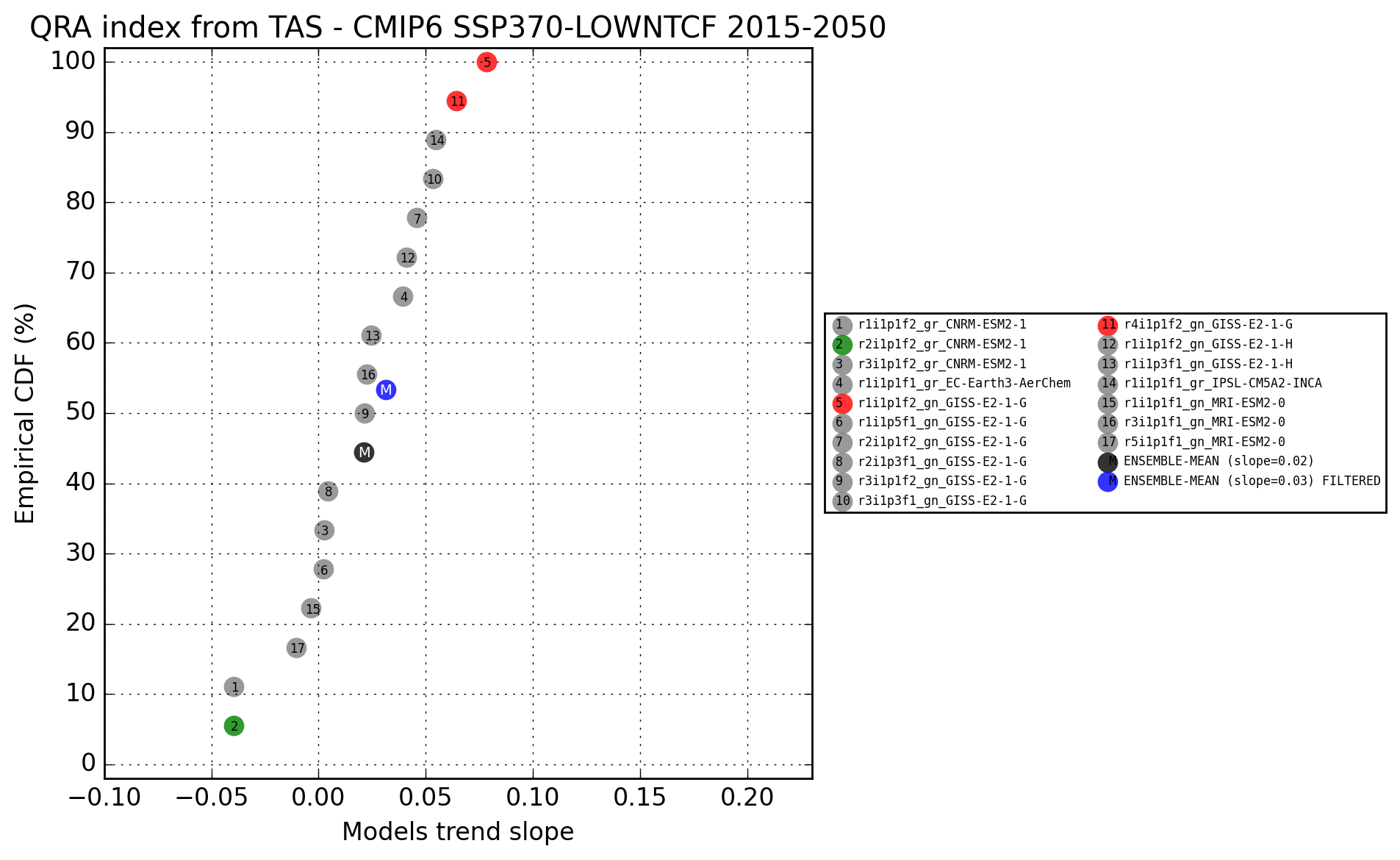

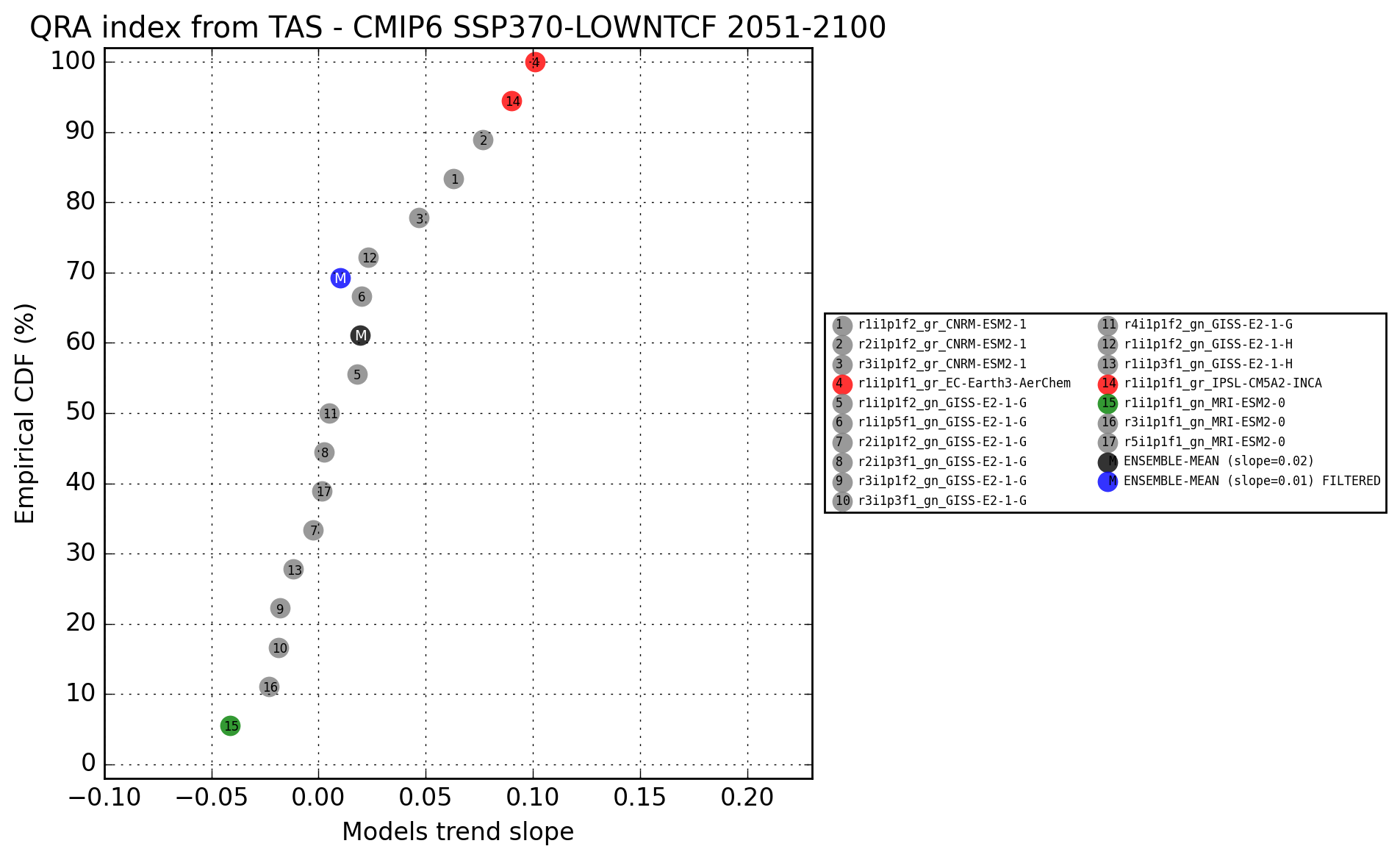
**

**Figure S24:** Average QRA index from Temperature JJA seasonal means for CMIP6 SSP370-lowNTCF.

| **(A)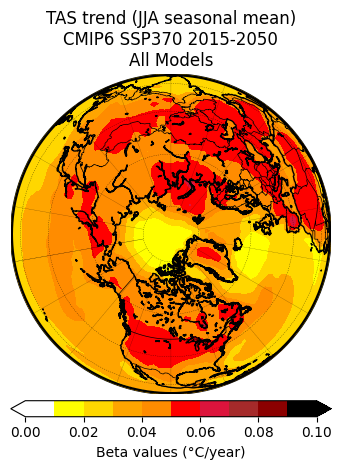** | **(D)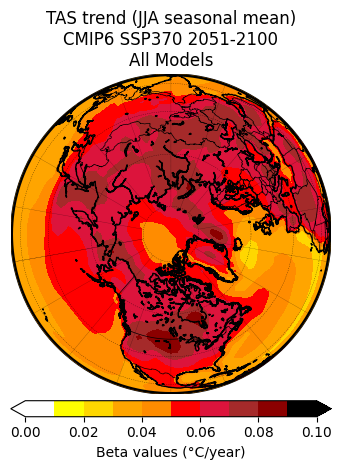** |
| --- | --- |
| **(B)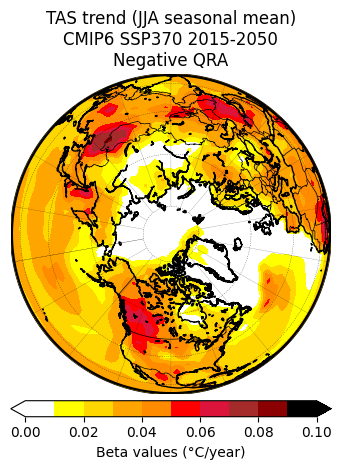** | **(E)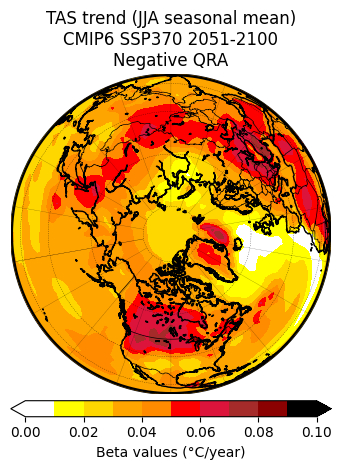** |
| **(C)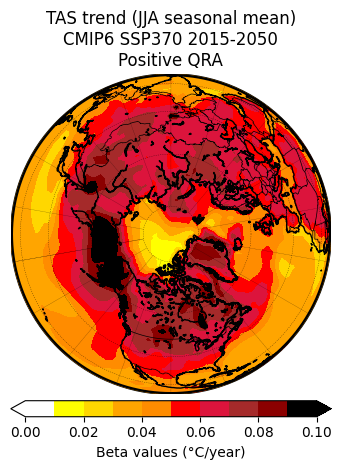** | **(F)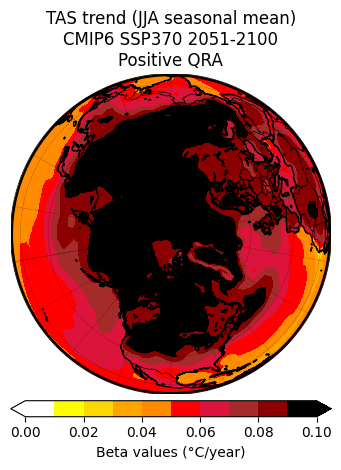** |
| **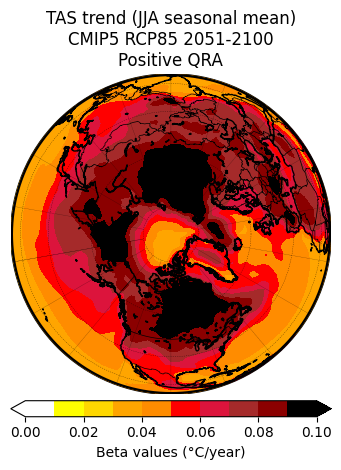** | |

**Figure S25:** Mean surface temperature trend patterns (JJA seasonal means) for CMIP6 SSP370. (A,D) multimodel ensemble, (B,E) most negative QRA-trending ensemble members, and (C,F) most positive QRA-trending ensemble members (“most” is defined as upper 10th percentile of multimodel ensemble).

**
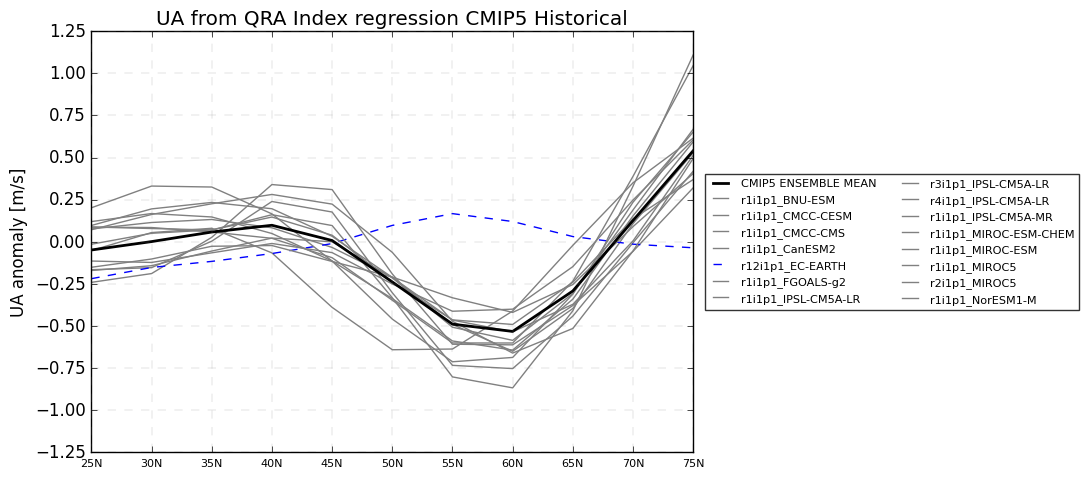
**

**Figure S26:** Projection of QRA index from Temperature JJA seasonal means onto zonal wind anomalies for CMIP5 Historical.

**
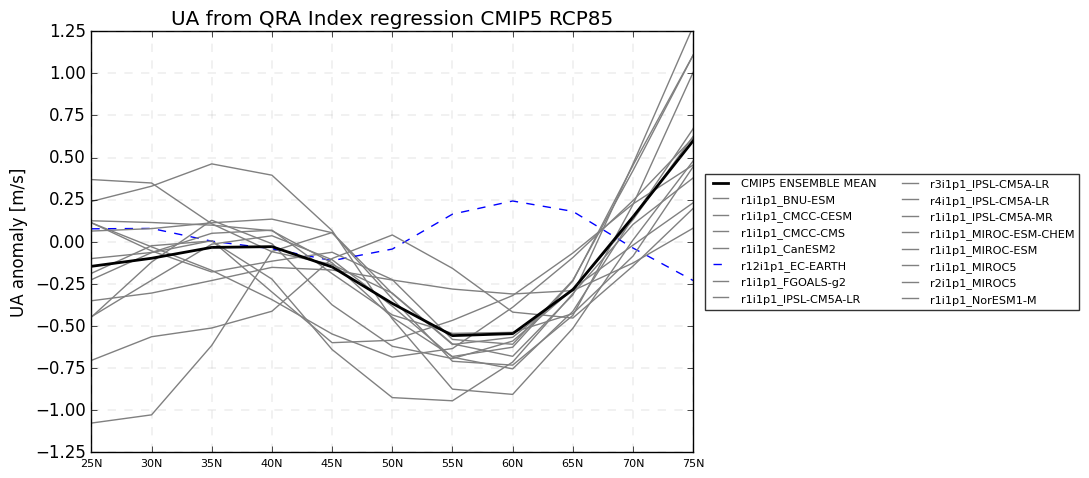
**

**Figure S27:** Projection of QRA index from Temperature JJA seasonal means onto zonal wind anomalies for CMIP5 RCP85.

**
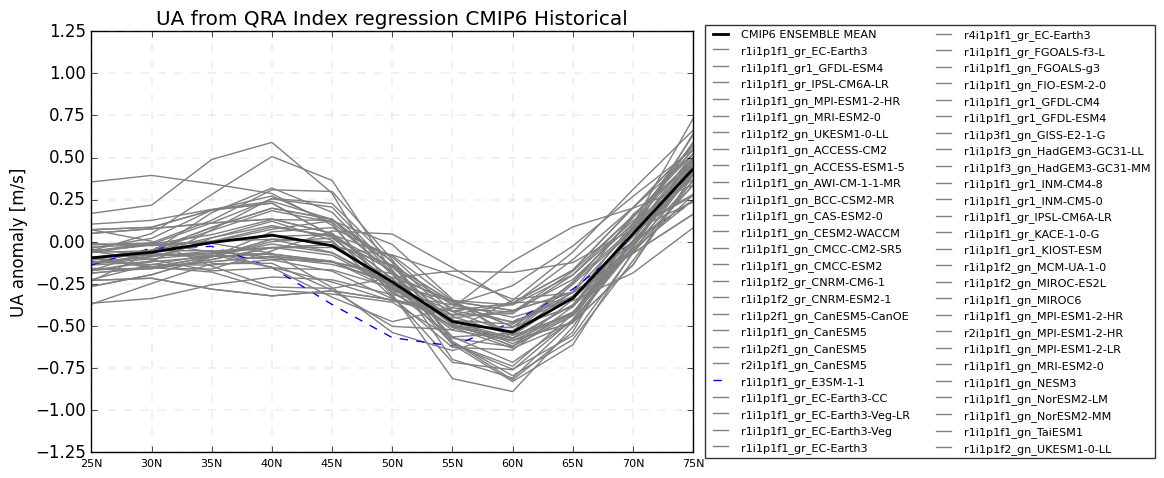
**

**Figure S28:** Projection of QRA index from Temperature JJA seasonal means onto zonal wind anomalies for CMIP6 Historical.

**
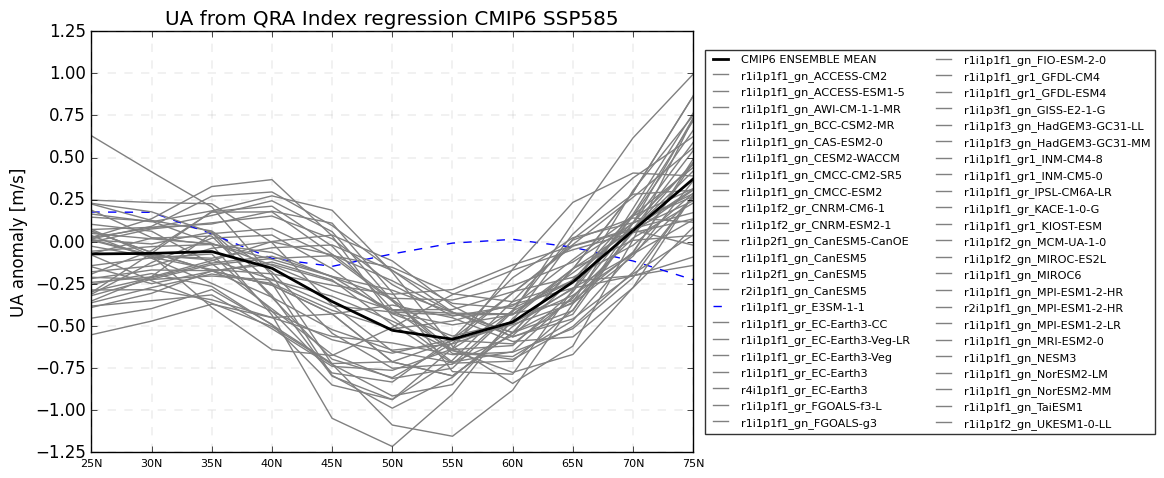
**

**Figure S29:** Projection of QRA index from Temperature JJA seasonal means onto zonal wind anomalies for CMIP6 SSP585.

**
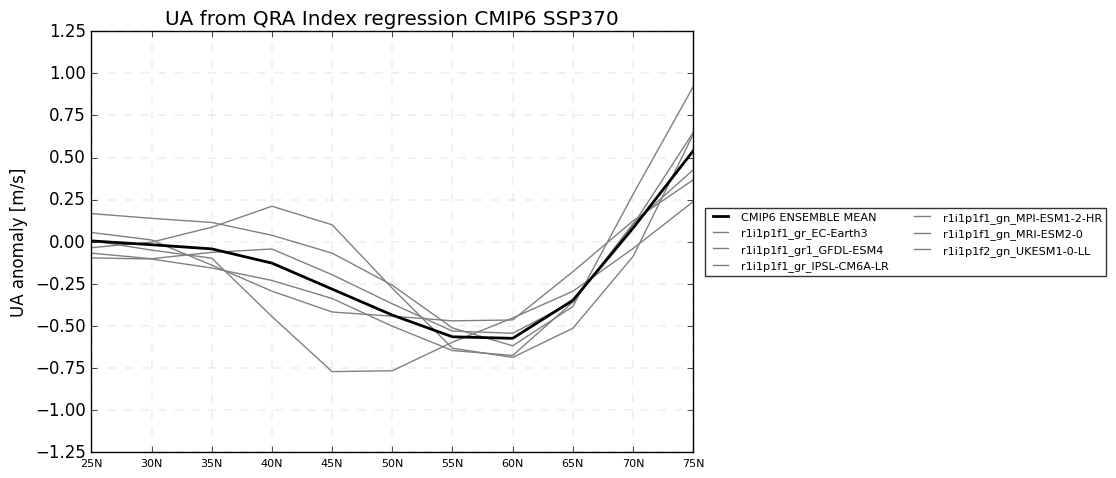
**

**Figure S30:** Projection of QRA index from Temperature JJA seasonal means onto zonal wind anomalies for CMIP6 SSP370.

| RMSE related to ERA-Interim | Mean error for  Ensemble Members | | Error for  Ensemble Mean | |
| --- | --- | --- | --- | --- |
|  | CMIP5 | CMIP6 | CMIP5 | CMIP6 |
| *T(φ)* | 0.5% (150) | 0.5% (66) | 0.3% (150) | 0.3% (66) |
| *dT(φ)/dφ* | -28.8% (150) | -26.4% (66) | -15.1% (150) | -13.6% (66) |
| *ū(φ)* | 28.0% (74) | 17.6% (66) | 19.1% (74) | 10.8% (66) |
| *dū(φ)/dφ* | 188.8% (74) | 169.3% (66) | 91.3% (74) | 118.0% (66) |
| *d²ū(φ)/dφ²* | -324.1% (74) | -271.5% (66) | -148.8% (74) | -194.6% (66) |

**Table S5:** Temperature *T(φ)* and zonal wind *ū(φ)* RMSE for CMIP5 (1979-2005) and CMIP6 (1979-2014) Historical multimodel ensemble over 25N-75N (2.5 degrees) JJA seasonal means compared to ERA-Interim (1979-2014).

| RMSE related to ERA5 | Mean error for  Ensemble Members | | Error for  Ensemble Mean | |
| --- | --- | --- | --- | --- |
|  | CMIP5 | CMIP6 | CMIP5 | CMIP6 |
| *T(φ)* | 0.4% (150) | 0.5% (66) | 0.2% (150) | 0.2% (66) |
| *dT(φ)/dφ* | -33.8% (150) | -30.6% (66) | -23.3% (150) | -20.6% (66) |
| *ū(φ)* | 28.0% (74) | 17.6% (66) | 19.0% (74) | 10.7% (66) |
| *dū(φ)/dφ* | 192.4% (74) | 172.8% (66) | 93.4% (74) | 120.8% (66) |
| *d²ū(φ)/dφ²* | -341.3% (74) | -285.3% (66) | -160.0% (74) | -205.2% (66) |

**Table S6:** Temperature *T(φ)* and zonal wind *ū(φ)* RMSE for CMIP5 (1979-2005) and CMIP6 (1979-2014) Historical multimodel ensemble over 25N-75N (2.5 degrees) JJA seasonal means compared to ERA5 (1979-2014).

**
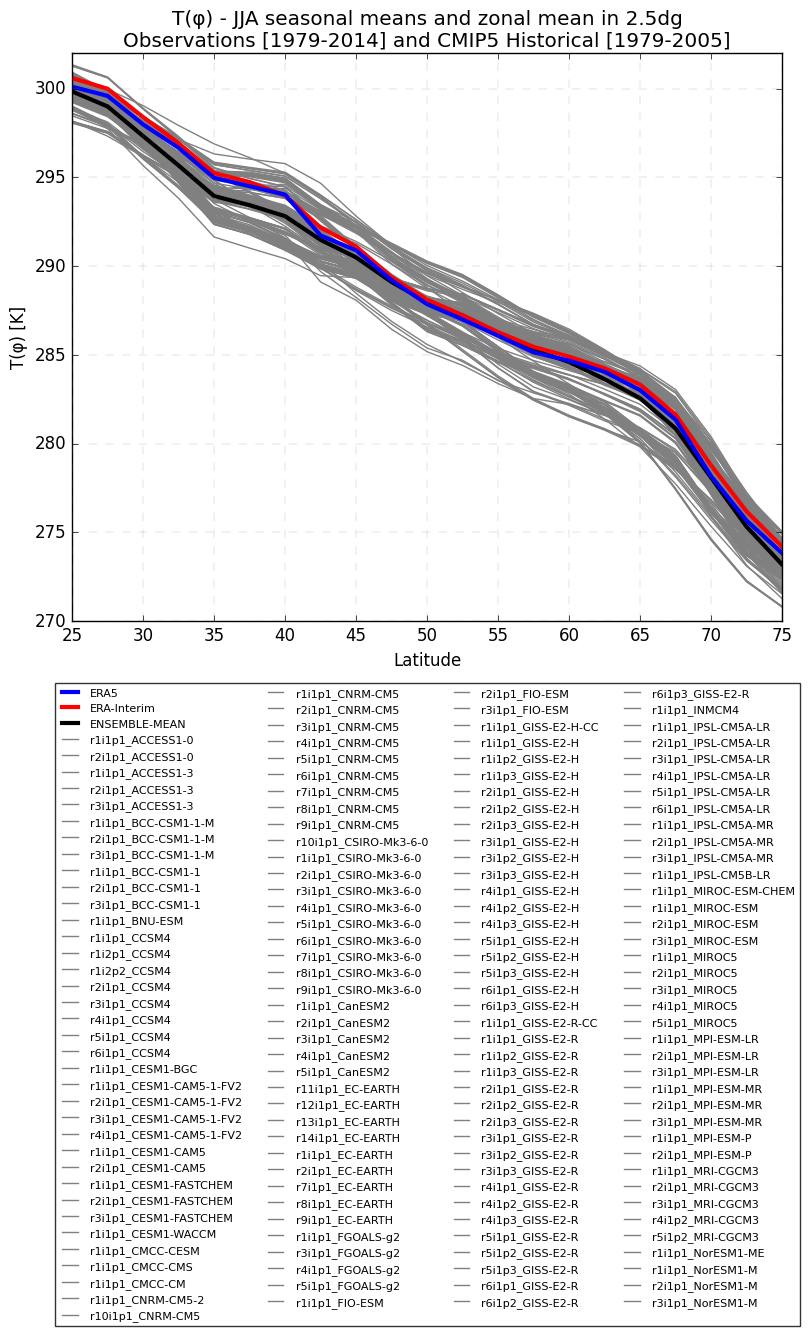
**

**Figure S31:** Temperature *T(φ)* for CMIP5 (1979-2005) Historical multimodel ensemble over 25N-75N (2.5 degrees) JJA seasonal means compared to observations.

**
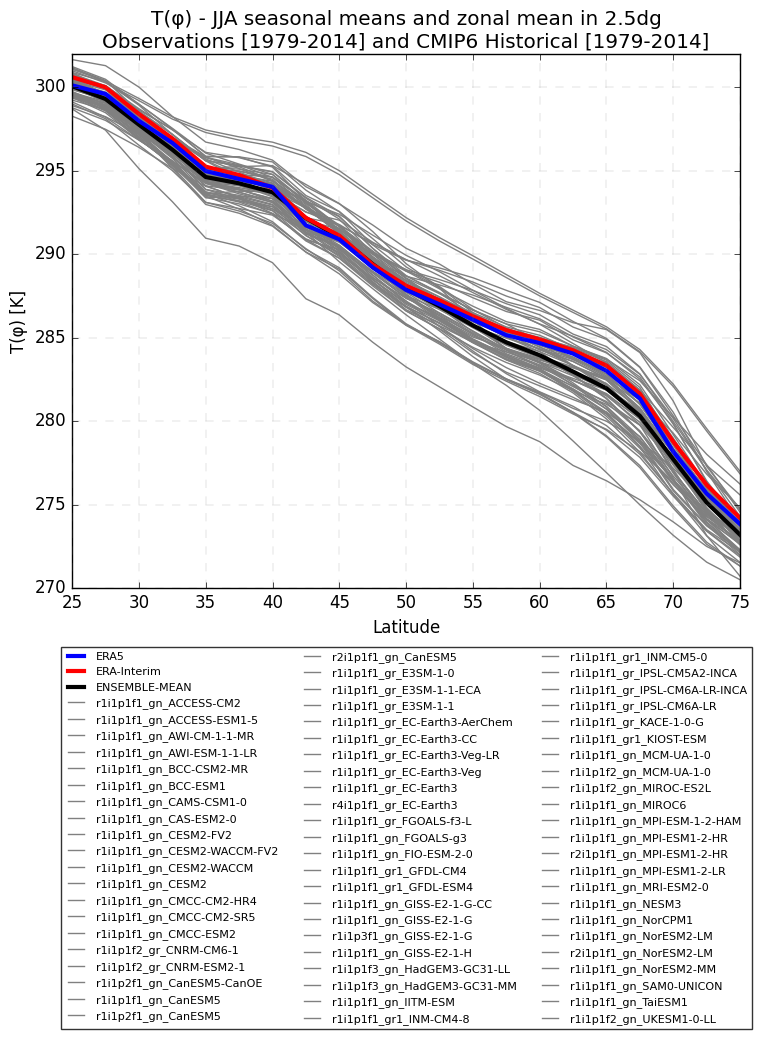
**

**Figure S32:** Temperature *T(φ)* for CMIP6 (1979-2014) Historical multimodel ensemble over 25N-75N (2.5 degrees) JJA seasonal means compared to observations.

**
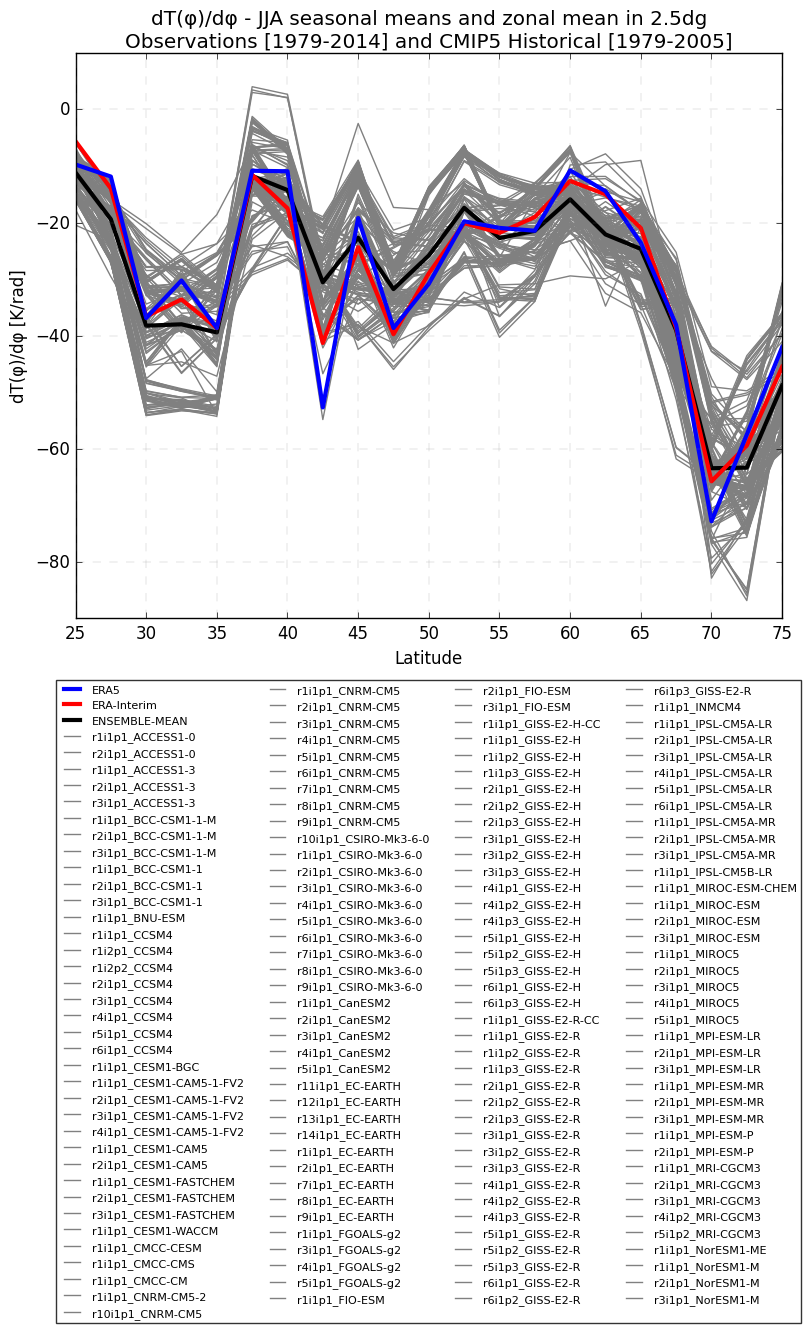
**

**Figure S33:** Temperature *dT(φ)/dφ* for CMIP5 (1979-2005) Historical multimodel ensemble over 25N-75N (2.5 degrees) JJA seasonal means compared to observations.

**
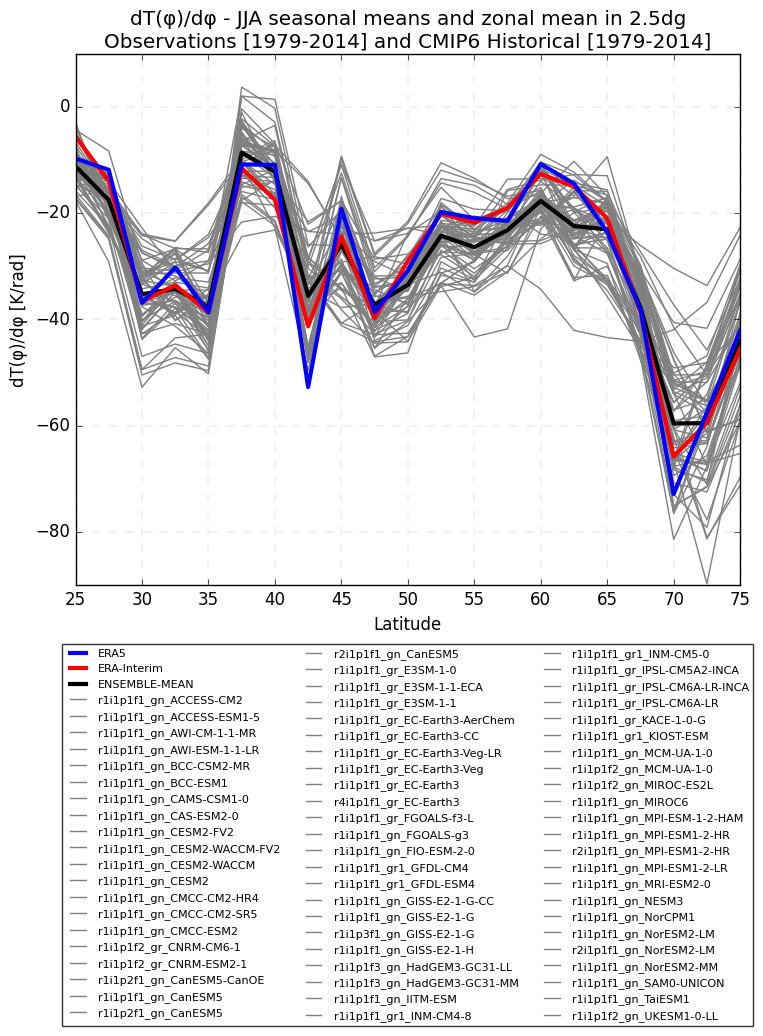
**

**Figure S34:** Temperature *dT(φ)/dφ* for CMIP6 (1979-2014) Historical multimodel ensemble over 25N-75N (2.5 degrees) JJA seasonal means compared to observations.

**
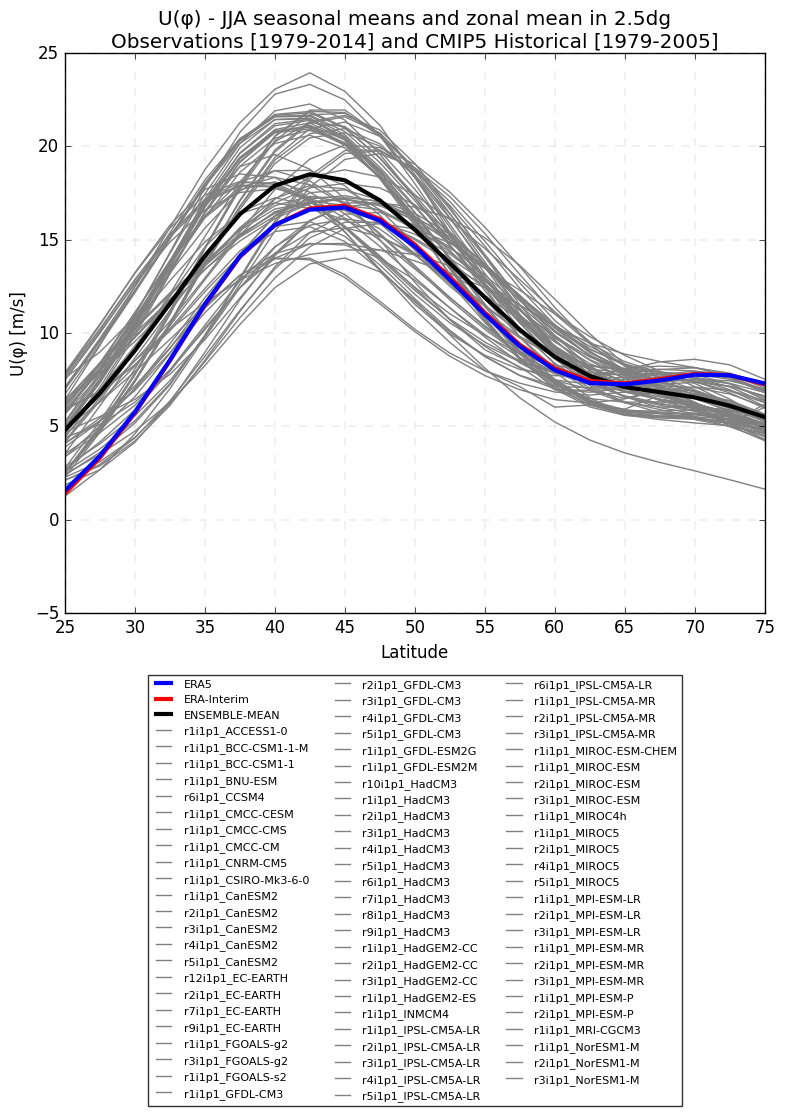
**

**Figure S35:** Zonal wind *ū(φ)* for CMIP5 (1979-2005) Historical multimodel ensemble over 25N-75N (2.5 degrees) JJA seasonal means compared to observations.

**
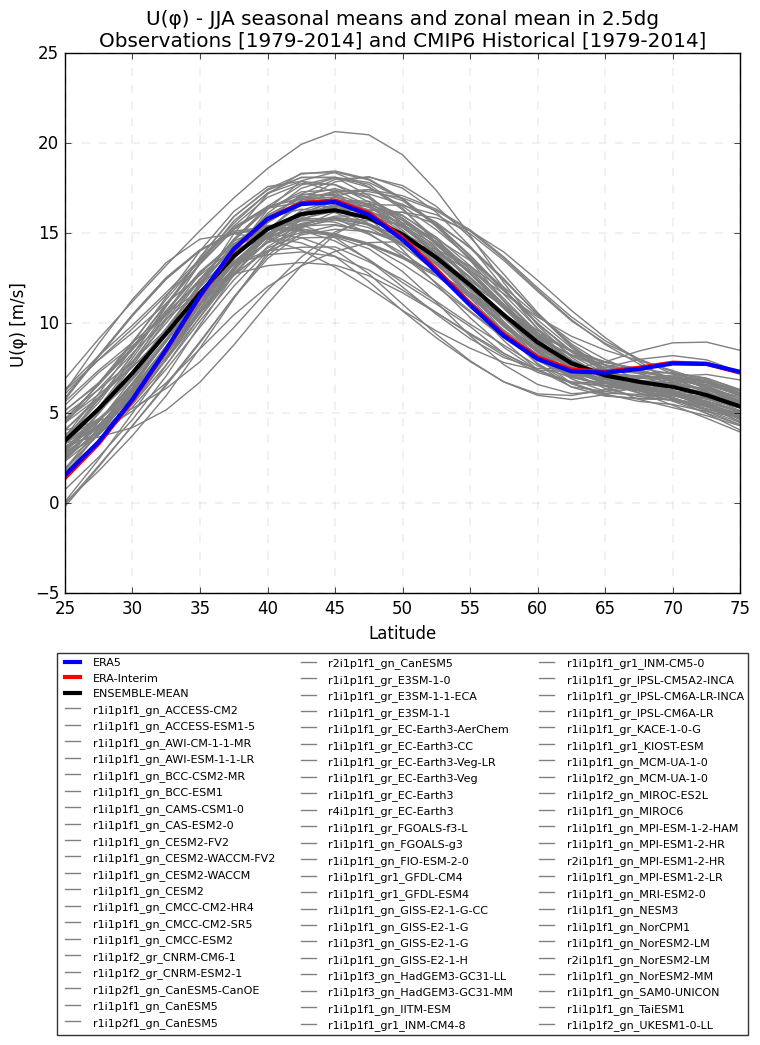
**

**Figure S36:** Zonal wind *ū(φ)* for CMIP6 (1979-2014) Historical multimodel ensemble over 25N-75N (2.5 degrees) JJA seasonal means compared to observations.

**
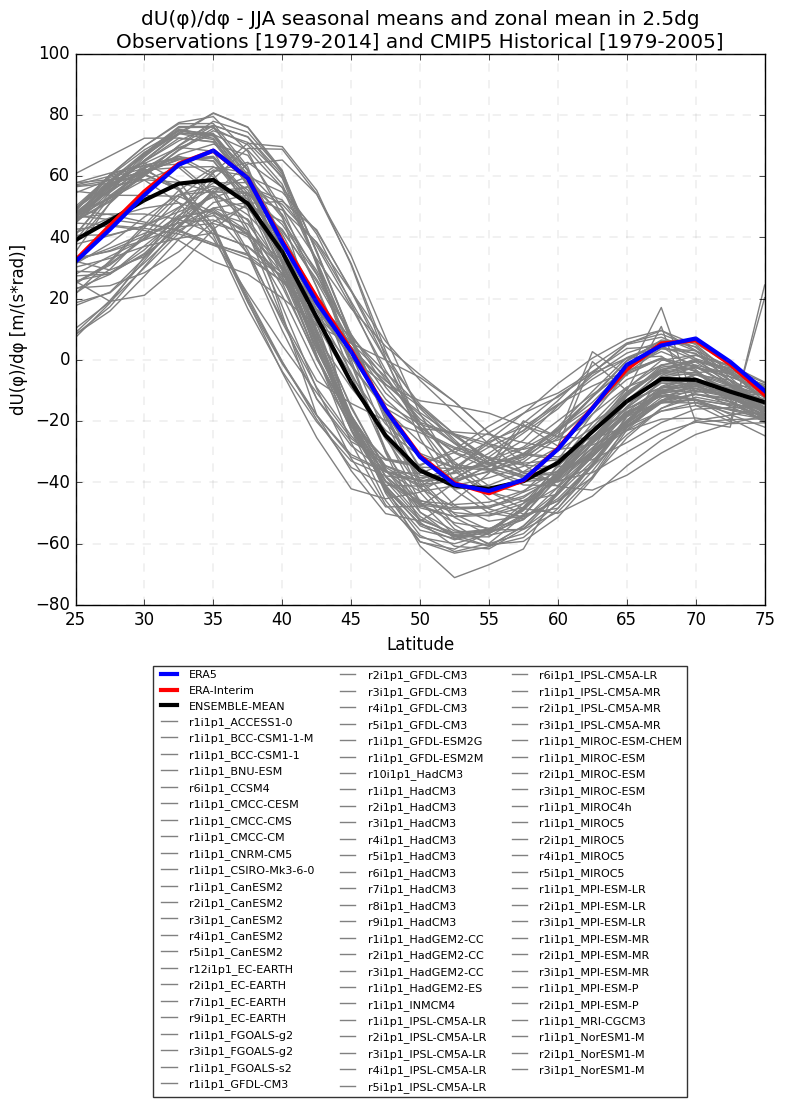
**

**Figure S37:** Zonal wind *dū(φ)/dφ* for CMIP5 (1979-2005) Historical multimodel ensemble over 25N-75N (2.5 degrees) JJA seasonal means compared to observations.

**
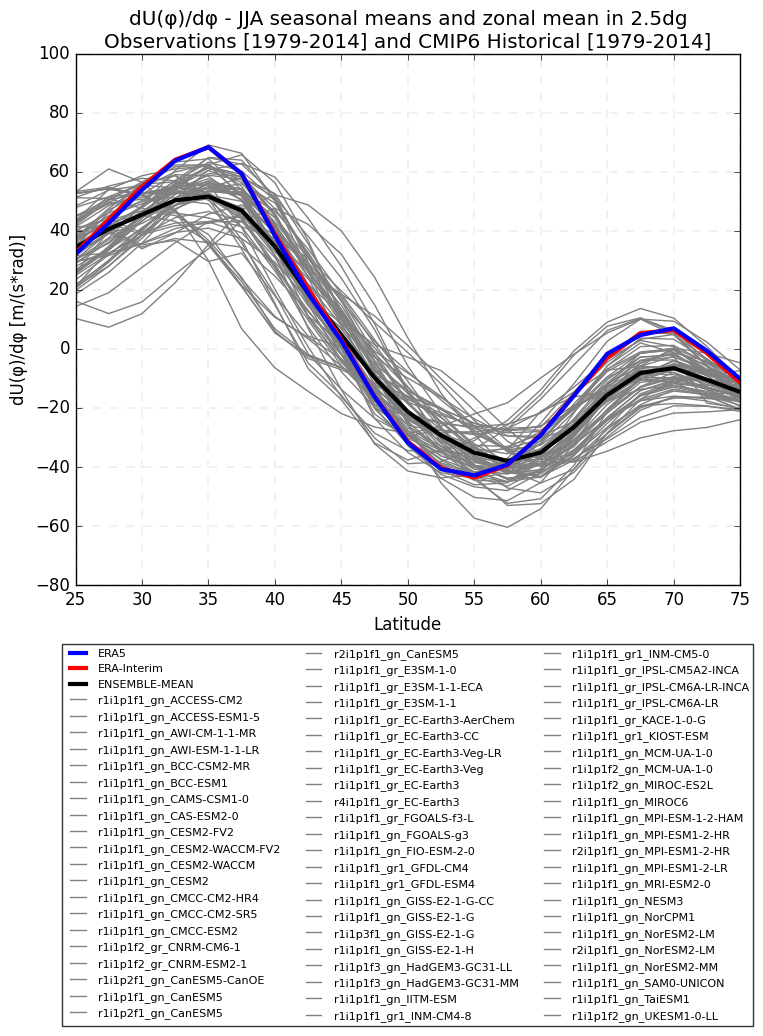
**

**Figure S38:** Zonal wind *dū(φ)/dφ* for CMIP6 (1979-2014) Historical multimodel ensemble over 25N-75N (2.5 degrees) JJA seasonal means compared to observations.

**
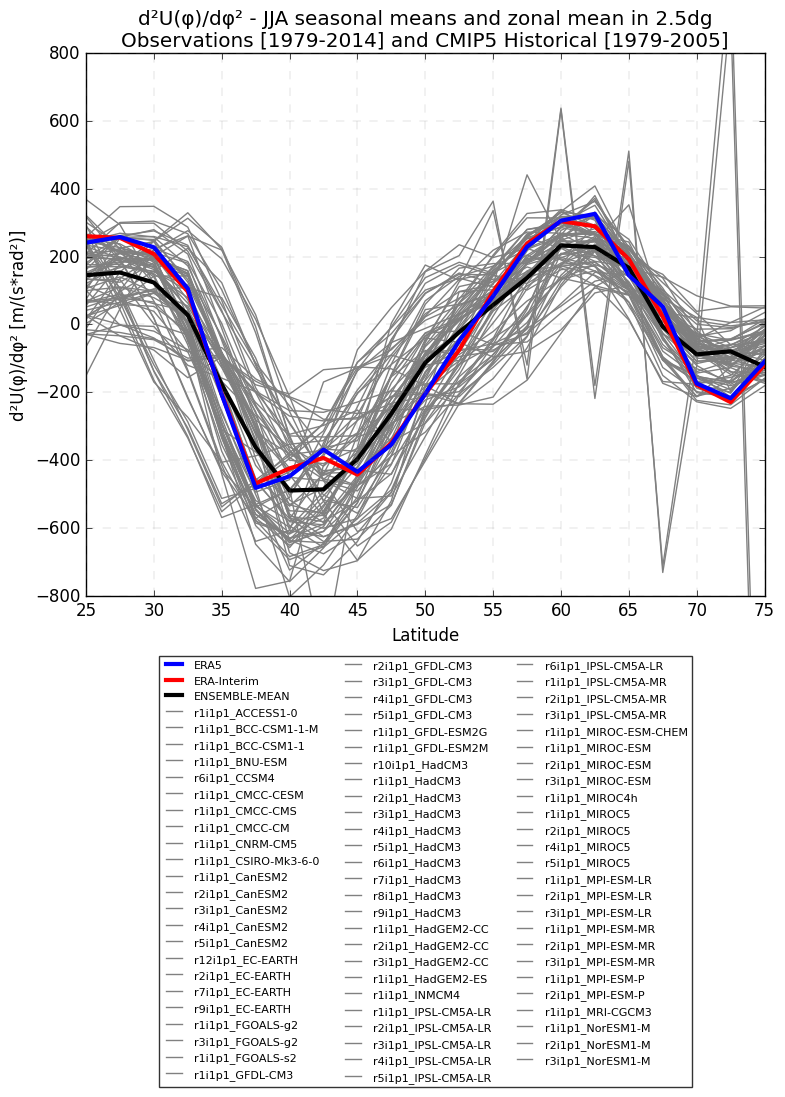
**

**Figure S39:** Zonal wind *d^2^ū(φ)/dφ^2^* for CMIP5 (1979-2005) Historical multimodel ensemble over 25N-75N (2.5 degrees) JJA seasonal means compared to observations.

**
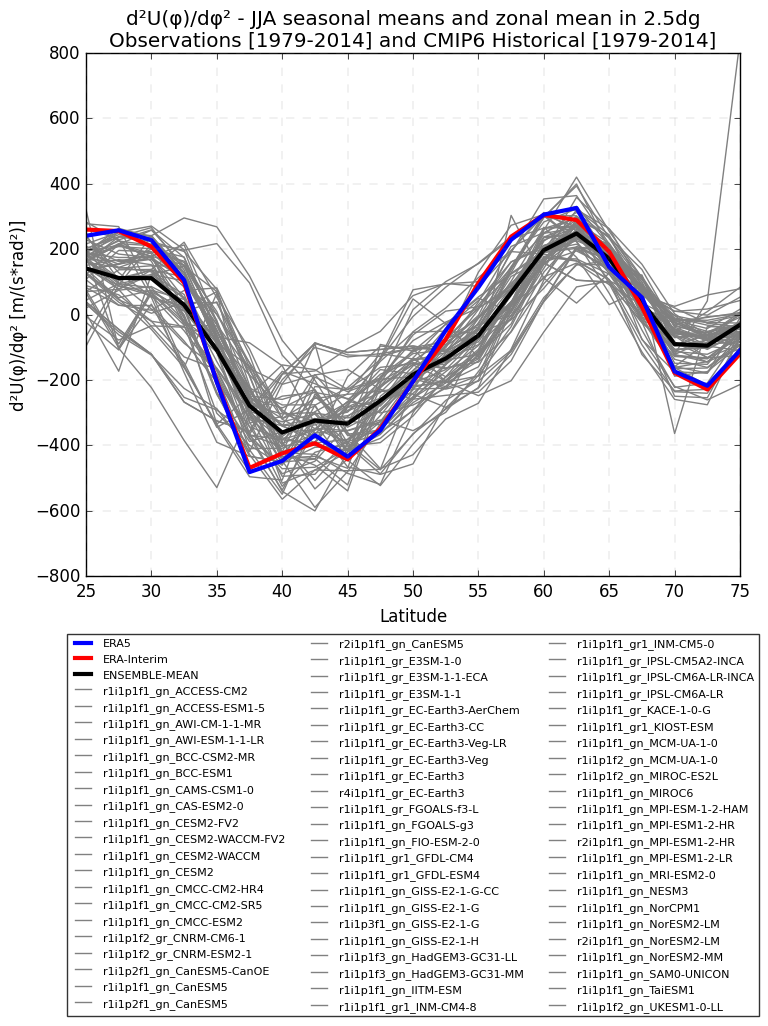
**

**Figure S40:** Zonal wind *d^2^ū(φ)/dφ^2^* for CMIP6 (1979-2014) Historical multimodel ensemble over 25N-75N (2.5 degrees) JJA seasonal means compared to observations.

**
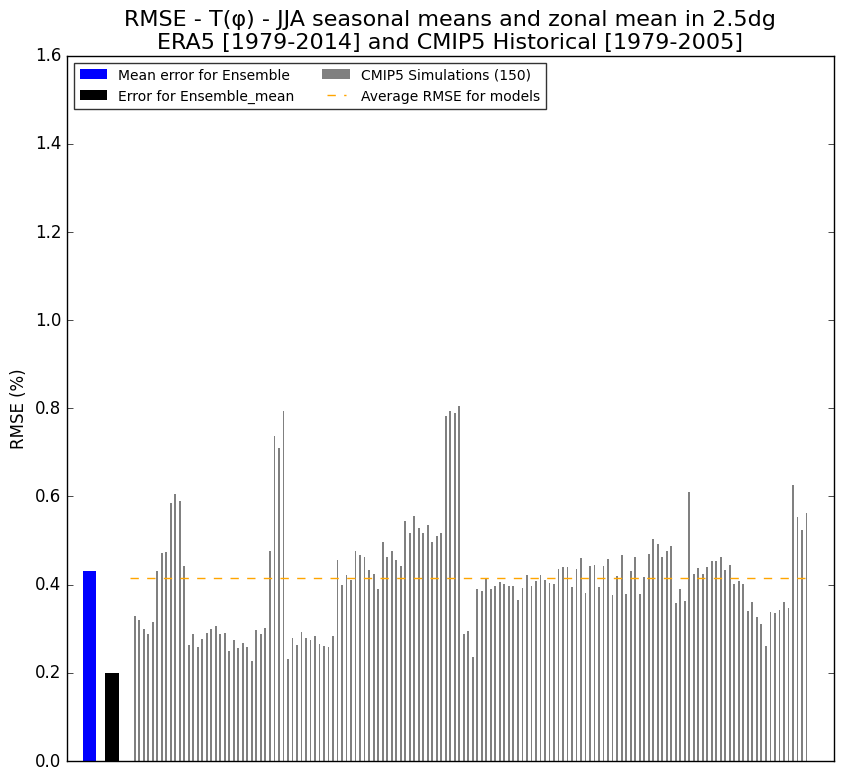

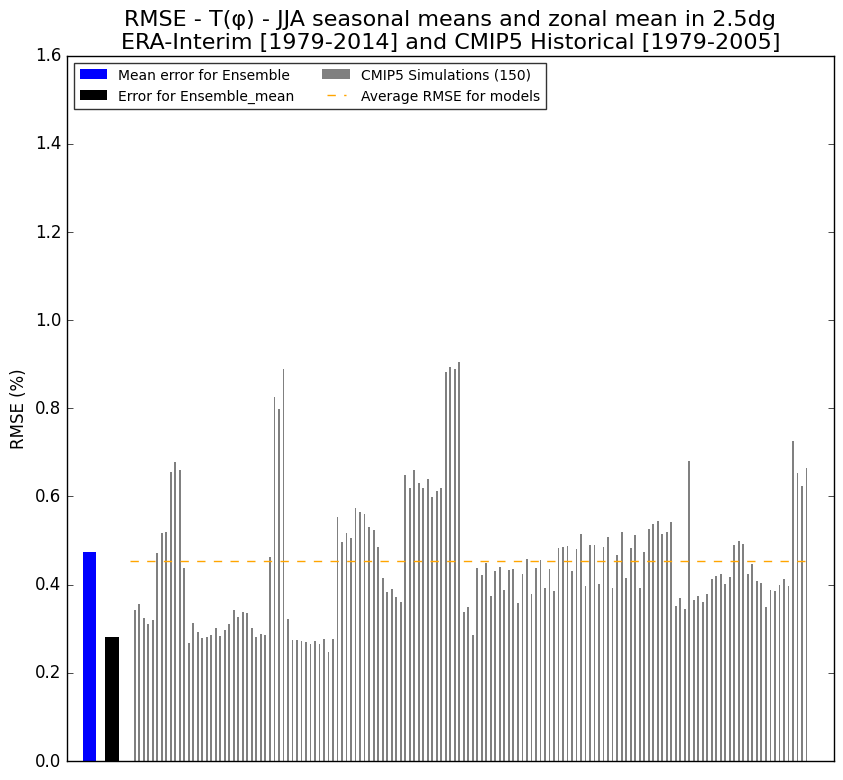
**

**Figure S41:** Temperature *T(φ)* RMSE for CMIP5 (1979-2005) Historical multimodel ensemble over 25N-75N (2.5 degrees) JJA seasonal means compared to ERA5 and ERA-Interim.

**
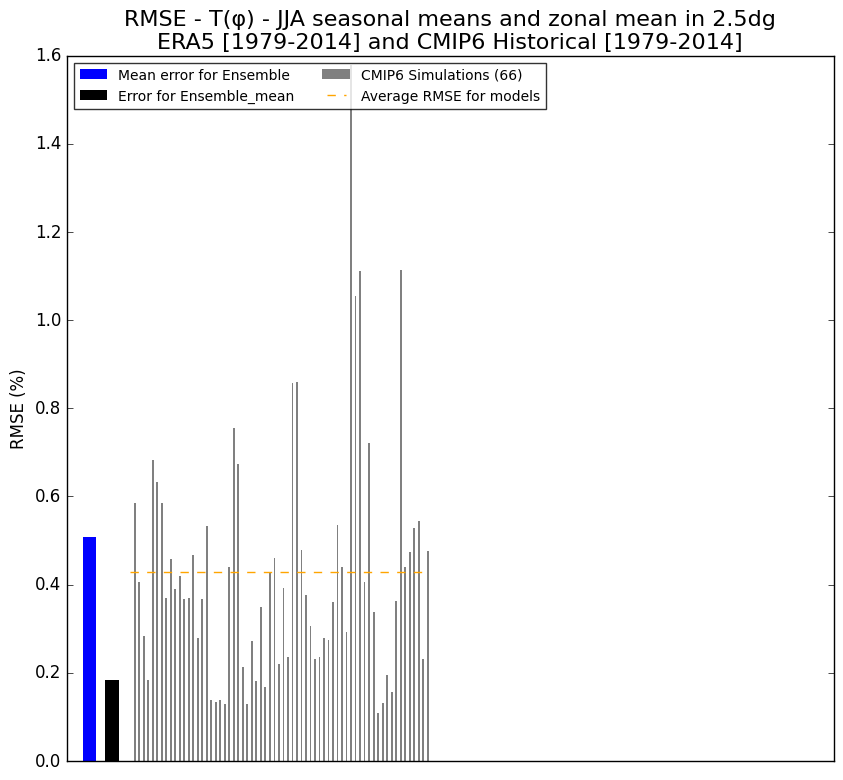

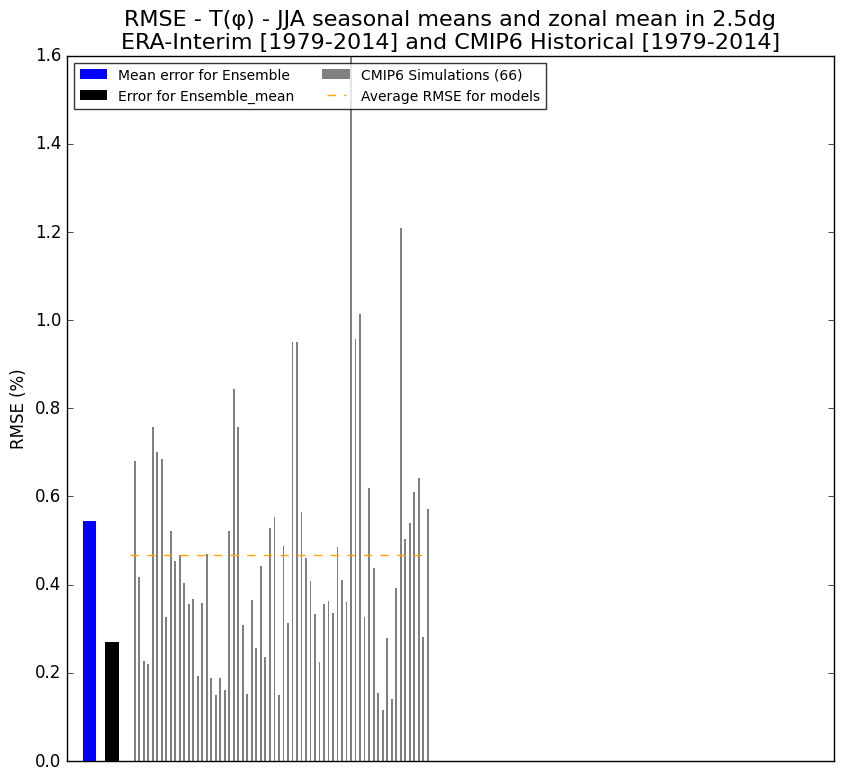
**

**Figure S42:** Temperature *T(φ)* RMSE for CMIP6 (1979-2014) Historical multimodel ensemble over 25N-75N (2.5 degrees) JJA seasonal means compared to ERA5 and ERA-Interim.

**
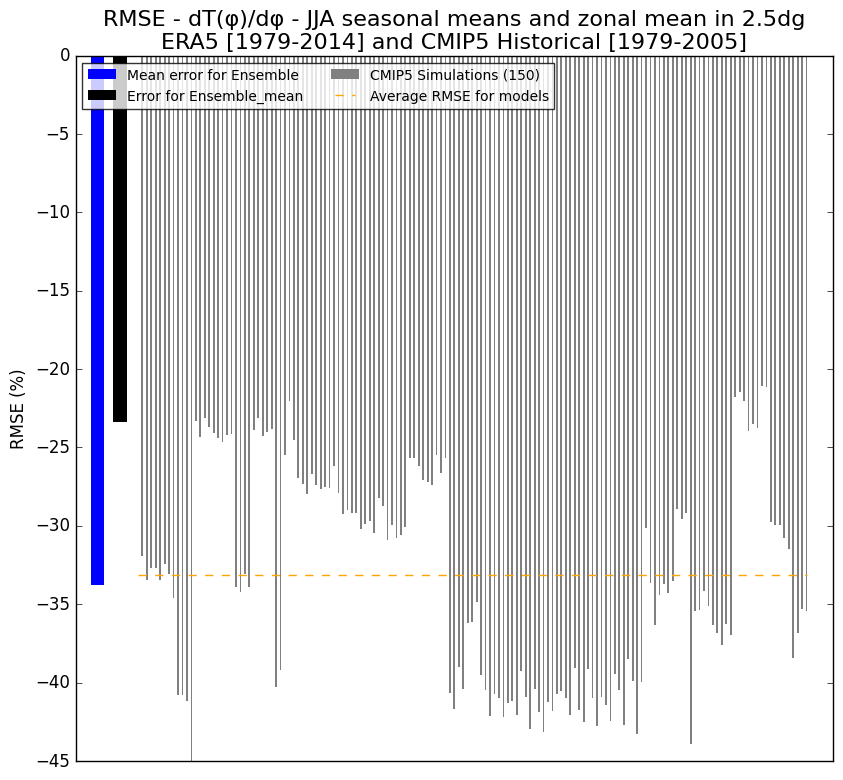

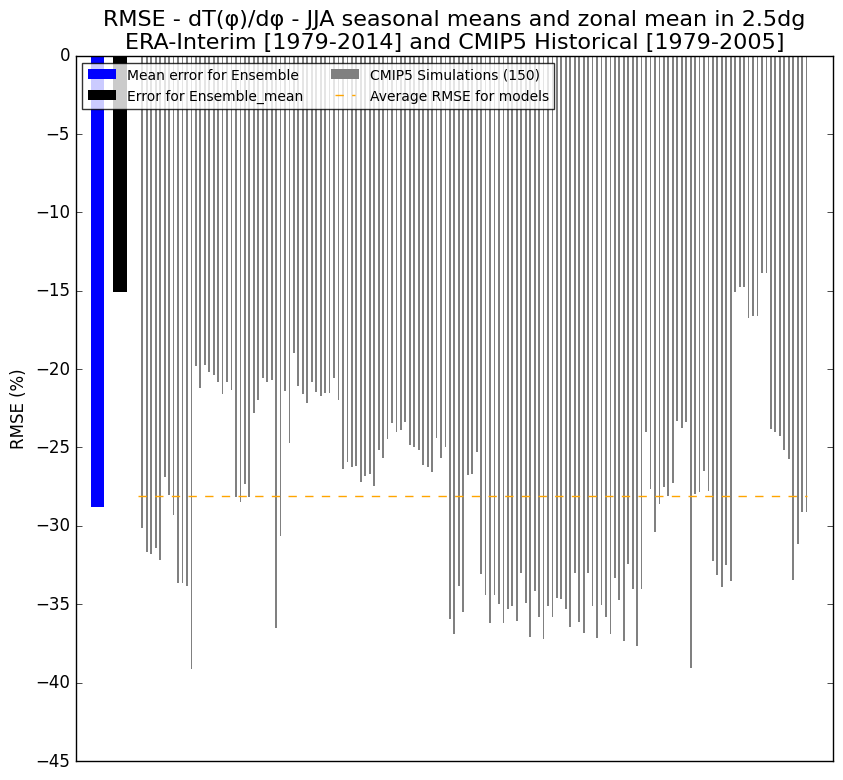
**

**Figure S43:** Temperature *dT(φ)/dφ* RMSE for CMIP5 (1979-2005) Historical multimodel ensemble over 25N-75N (2.5 degrees) JJA seasonal means compared to ERA5 and ERA-Interim.

**
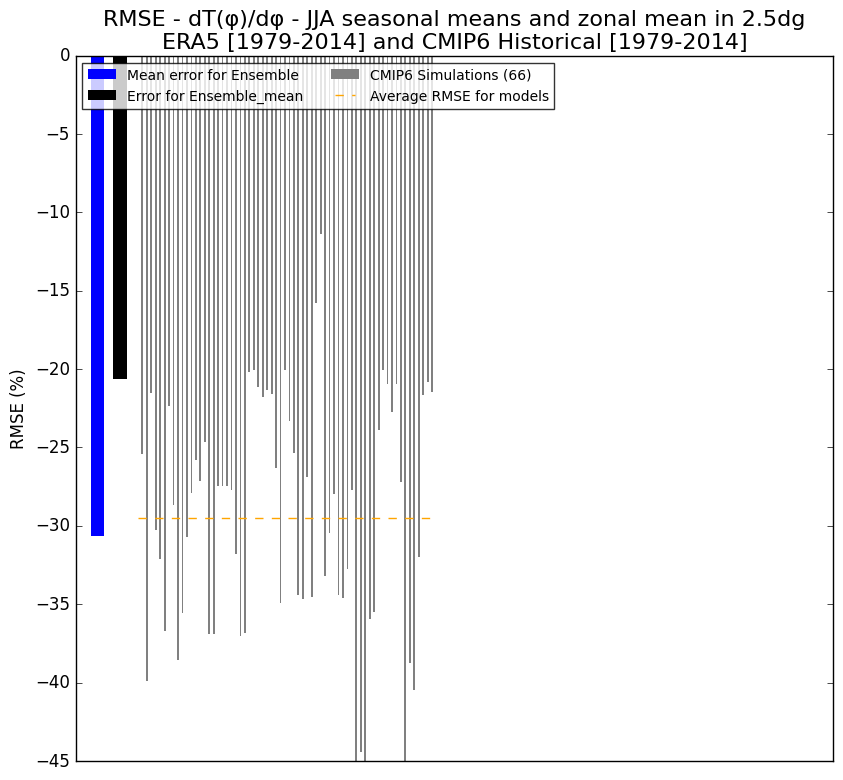

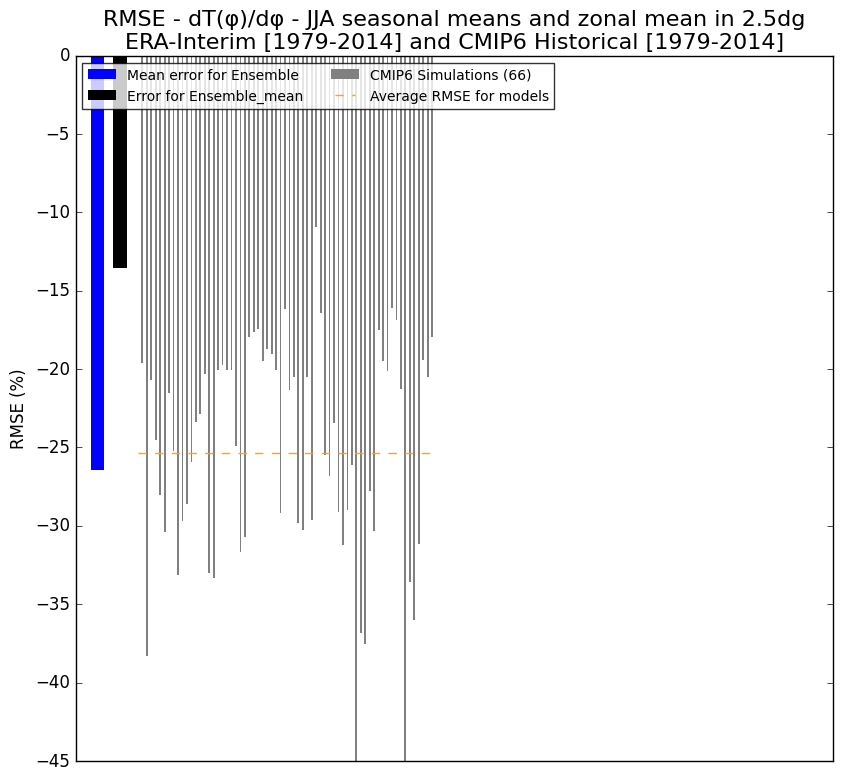
**

**Figure S44:** Temperature *dT(φ)/dφ* RMSE for CMIP6 (1979-2014) Historical multimodel ensemble over 25N-75N (2.5 degrees) JJA seasonal means compared to ERA5 and ERA-Interim.

**
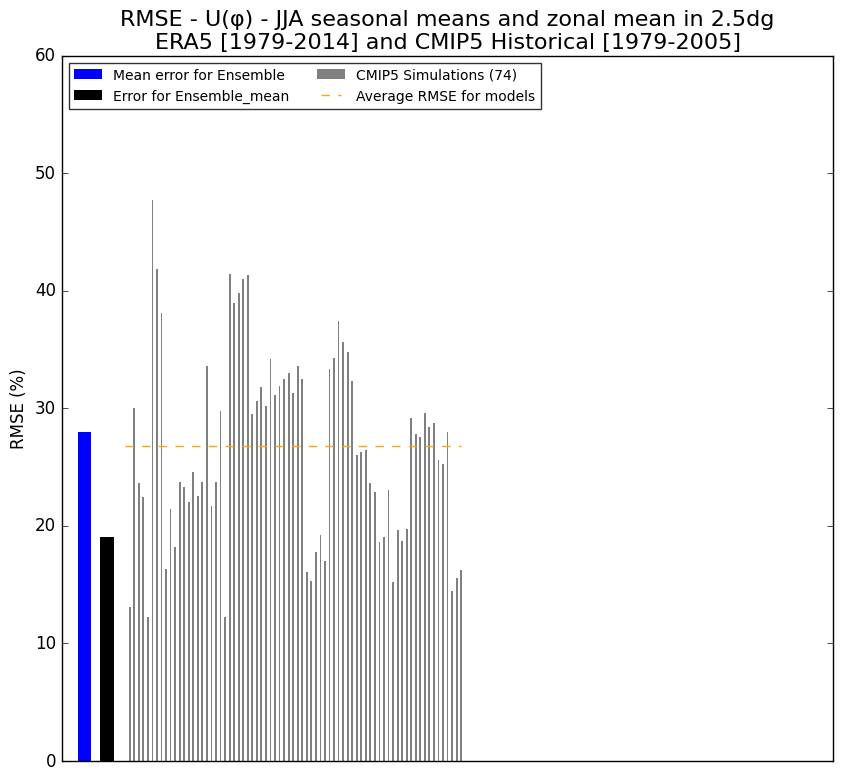

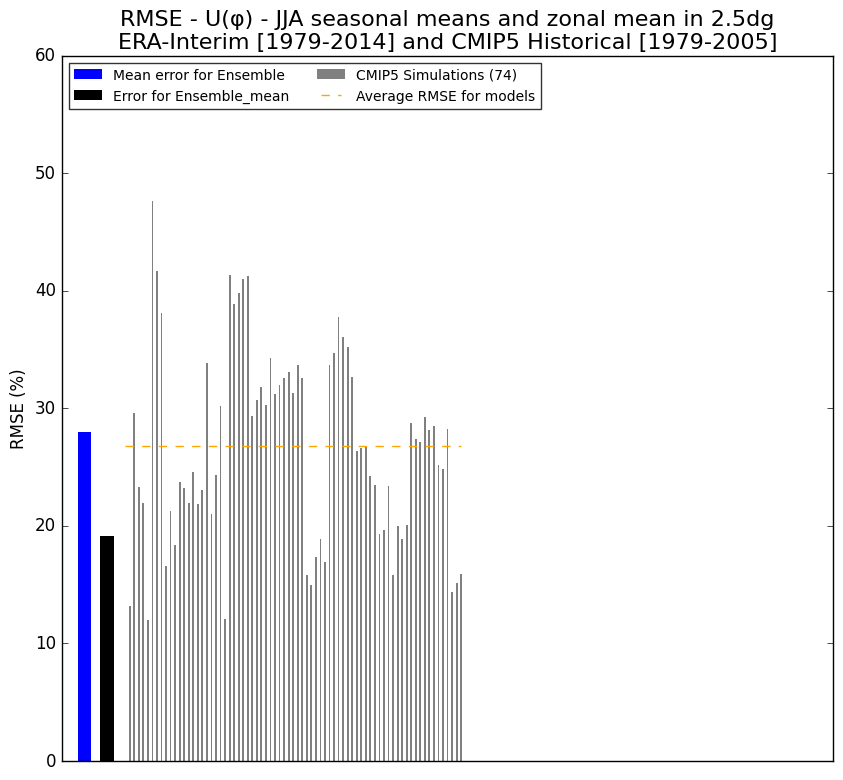
**

**Figure S45:** Zonal wind *ū(φ)* RMSE for CMIP5 (1979-2005) Historical multimodel ensemble over 25N-75N (2.5 degrees) JJA seasonal means compared to ERA5 and ERA-Interim.

**
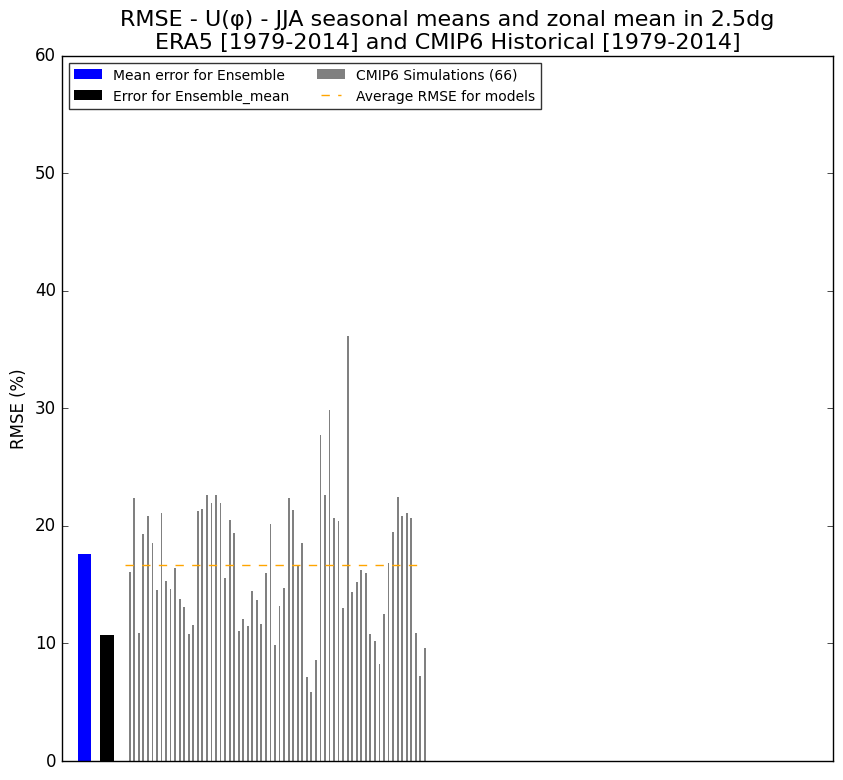

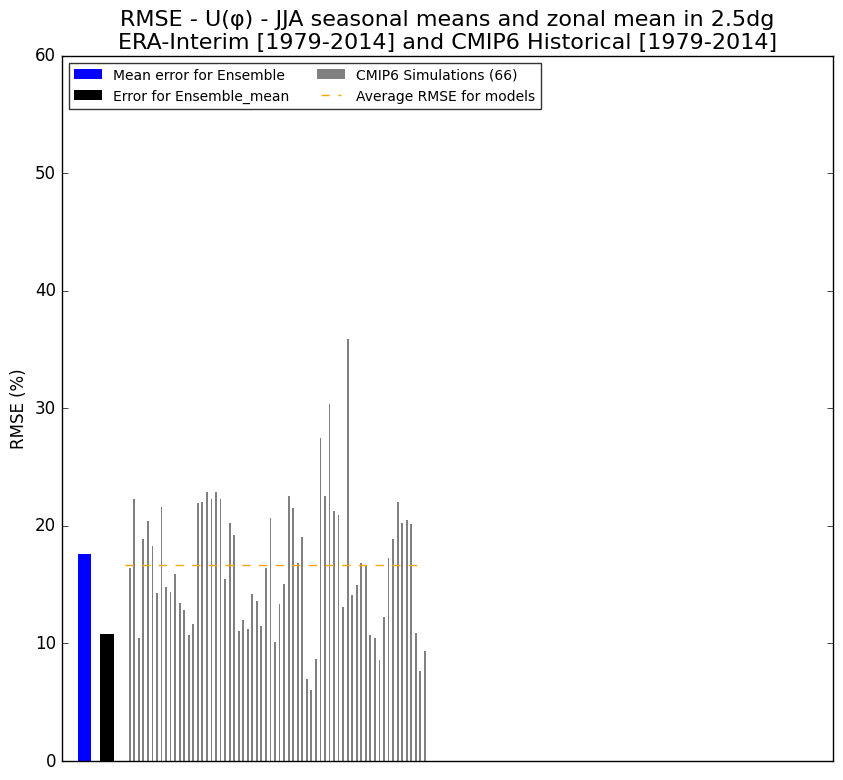
**

**Figure S46:** Zonal wind *ū(φ)* RMSE for CMIP6 (1979-2014) Historical multimodel ensemble over 25N-75N (2.5 degrees) JJA seasonal means compared to ERA5 and ERA-Interim.

**
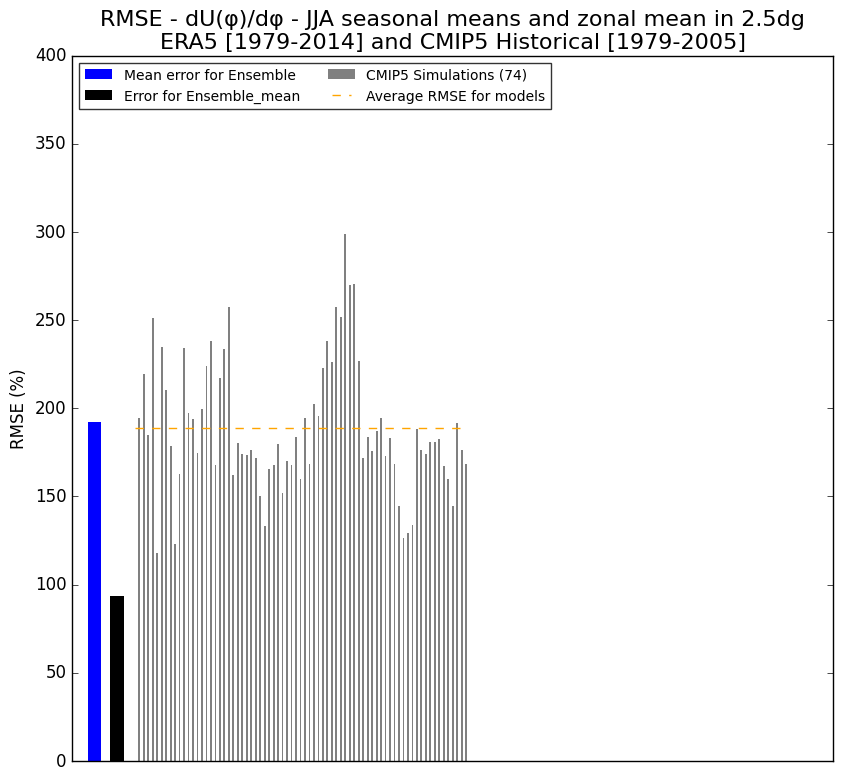

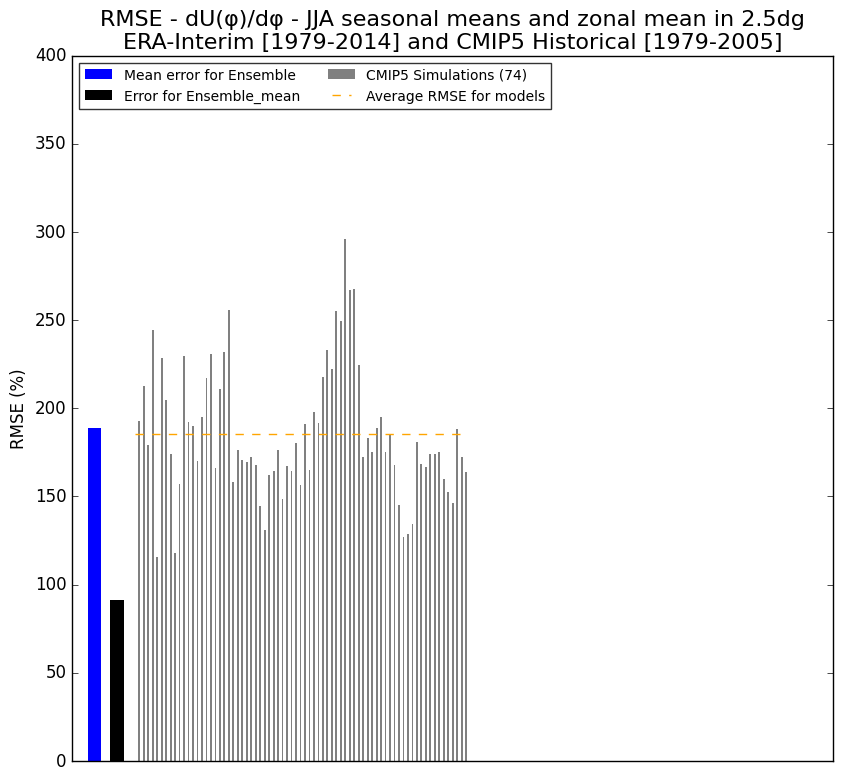
**

**Figure S47:** Zonal wind *dū(φ)/dφ* RMSE for CMIP5 (1979-2005) Historical multimodel ensemble over 25N-75N (2.5 degrees) JJA seasonal means compared to ERA5 and ERA-Interim.

**
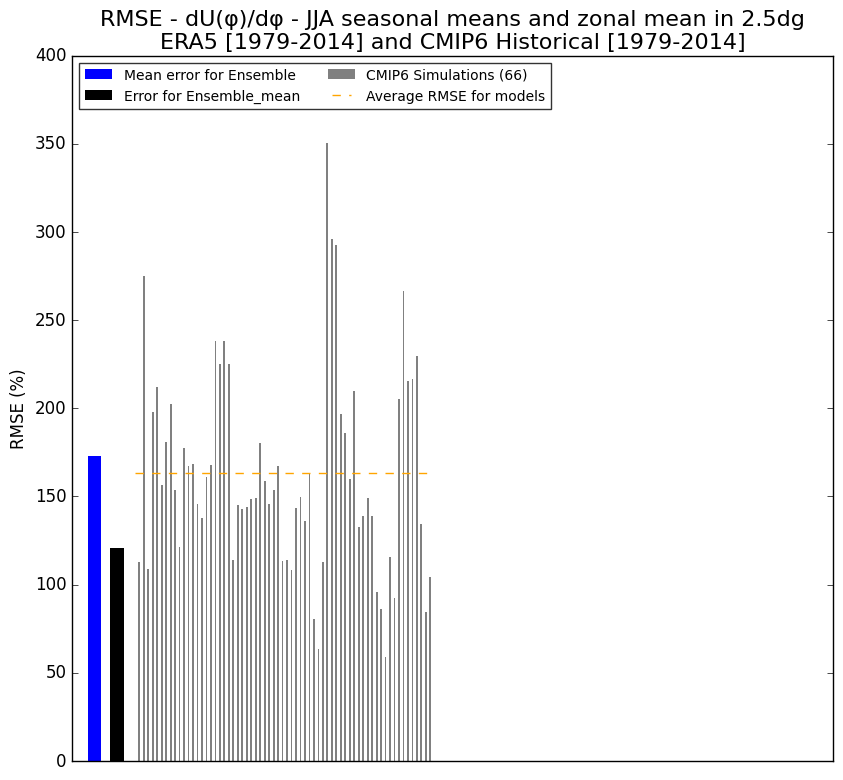

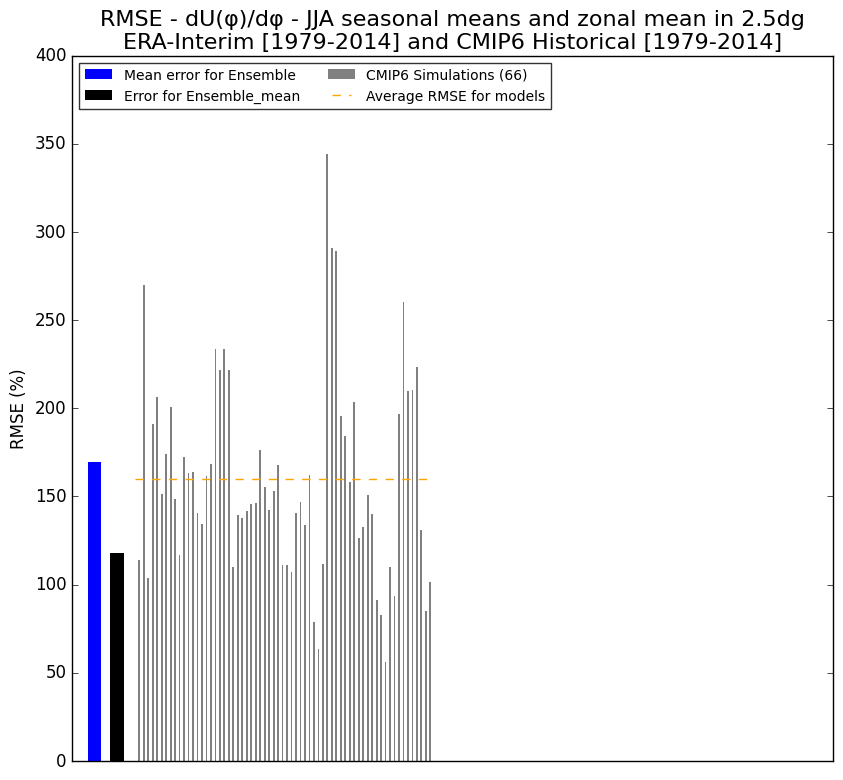
**

**Figure S48:** Zonal wind *dū(φ)/dφ* RMSE for CMIP6 (1979-2014) Historical multimodel ensemble over 25N-75N (2.5 degrees) JJA seasonal means compared to ERA5 and ERA-Interim.

**
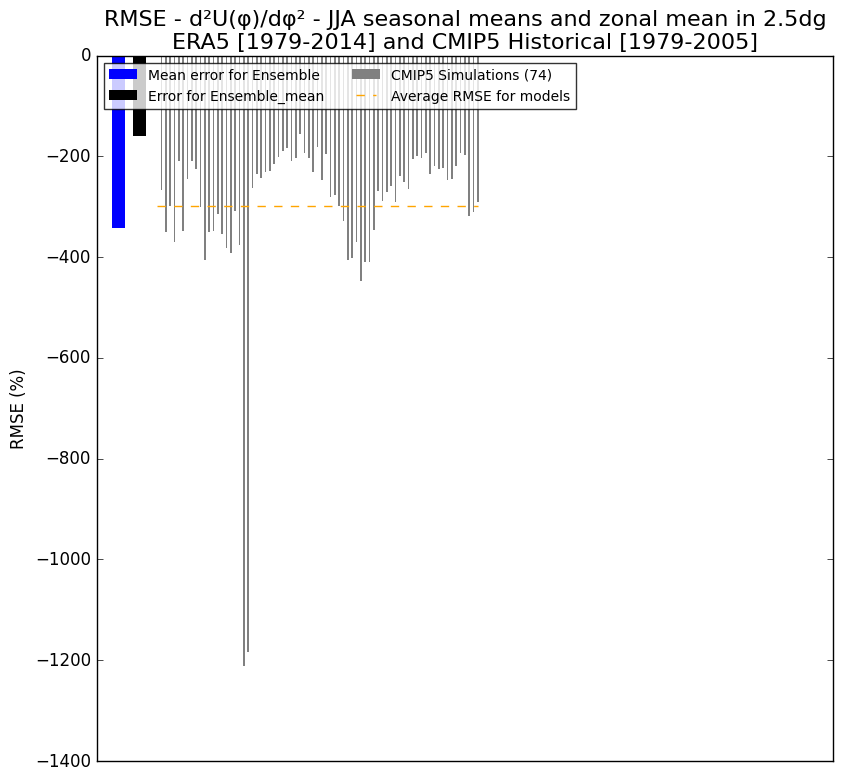

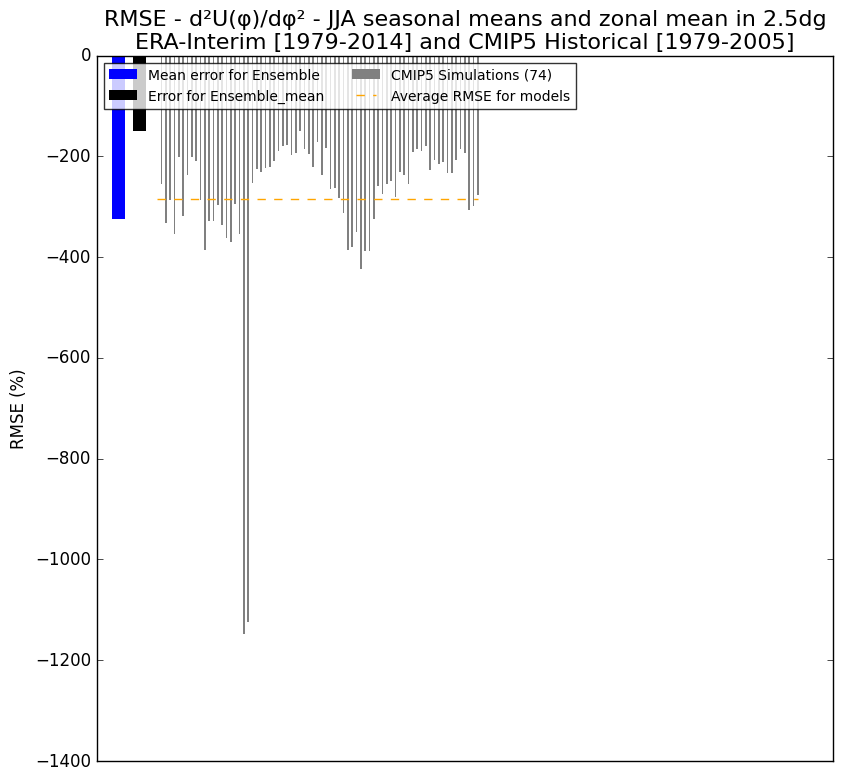
**

**Figure S49:** Zonal wind *d^2^ū(φ)/dφ^2^* RMSE for CMIP5 (1979-2005) Historical multimodel ensemble over 25N-75N (2.5 degrees) JJA seasonal means compared to ERA5 and ERA-Interim.

**
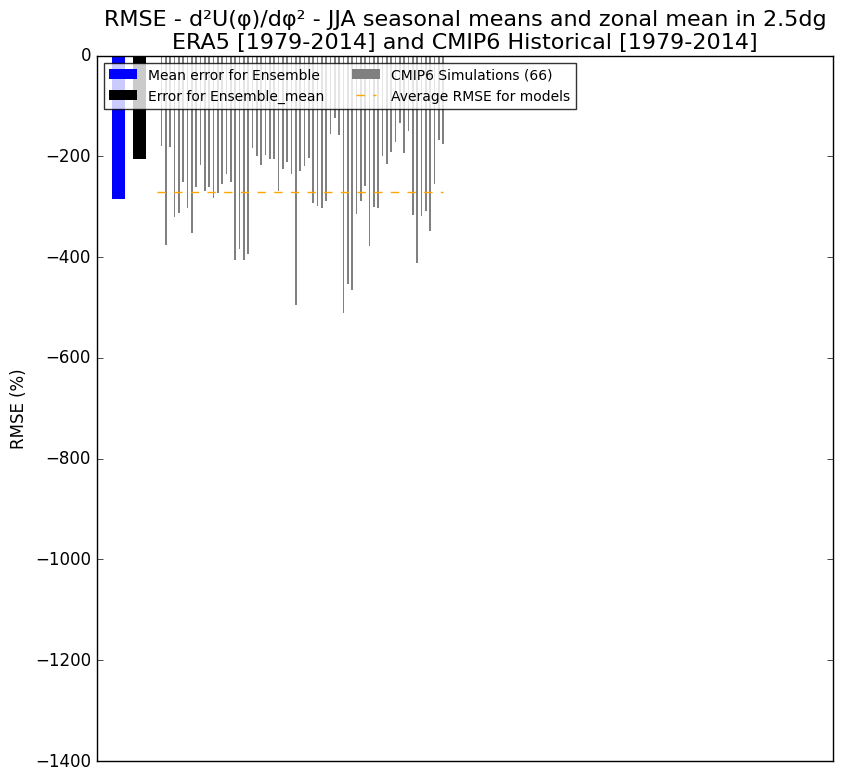

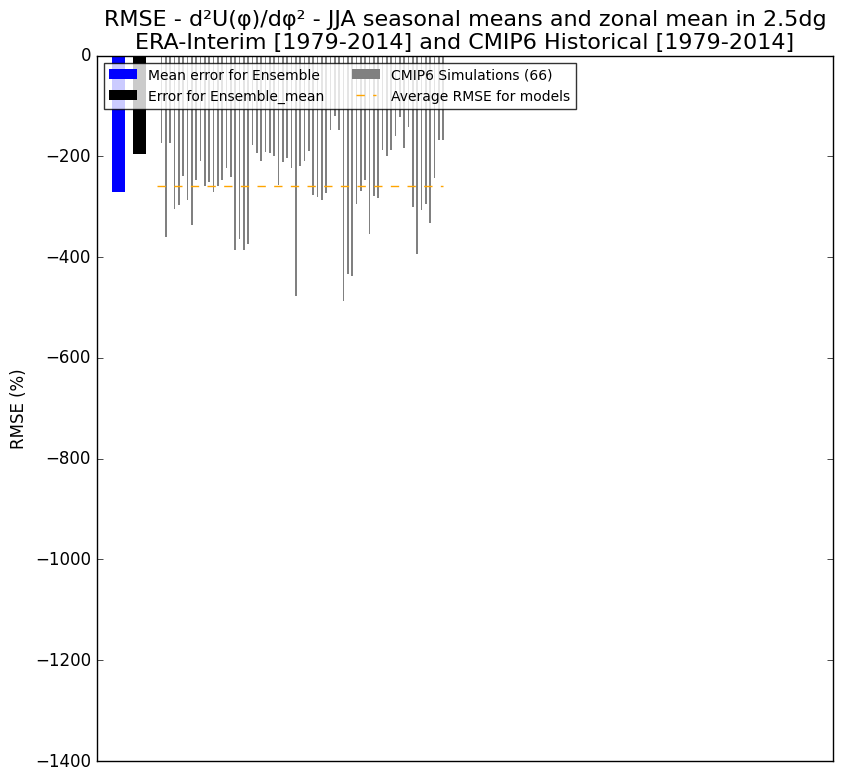
**

**Figure S50:** Zonal wind *d^2^ū(φ)/dφ^2^* RMSE for CMIP6 (1979-2014) Historical multimodel ensemble over 25N-75N (2.5 degrees) JJA seasonal means compared to ERA5 and ERA-Interim.

**References**

1. Hussain, M. M. *et al*. pyMannKendall: a python package for non parametric Mann Kendall family of trend tests. *Journal of Open Source Software*, **4**(39), 1556. DOI: 10.21105/joss.01556 (2019).

2. IPCC. *Annex II: Models* (eds. Gutiérrez, J M., A.-M. Tréguier). In *Climate Change 2021*: *The Physical Science Basis.* Contribution of Working Group I to the Sixth Assessment Report of the Intergovernmental Panel on Climate Change (eds. Masson-Delmotte, V. *et al*.). Cambridge University Press, 2087–2138, Table AII.5. DOI: 10.1017/9781009157896.016 (2021).

3. Flato, G. *et al*. *Evaluation of Climate Models.* In: *Climate Change 2013: The Physical Science Basis.* Contribution of Working Group I to the Fifth Assessment Report of the Intergovernmental Panel on Climate Change (eds. Stocker, T.F. *et al*.). Cambridge University Press, 741-882, Table 9.A.1, DOI: 10.1017/CBO9781107415324.020 (2013).

4. Mann, M. E. *et al*. Influence of Anthropogenic Climate Change on Planetary Wave Resonance and Extreme Weather Events. *Science Advances*, **7**, 45242. DOI: 10.1038/srep45242 (2017).
